# The Health Benefits of Probiotic *Lactiplantibacillus plantarum*: A Systematic Review and Meta-Analysis

**DOI:** 10.1007/s12602-024-10287-3

**Published:** 2024-05-31

**Authors:** Amal Aljohani, Noha Rashwan, Shruti Vasani, Ahmed Alkhawashki, Tong Tong Wu, Xingyi Lu, Daniel A. Castillo, Jin Xiao

**Affiliations:** 1 Eastman Institute for Oral Health, University of Rochester Medical Center, Rochester, NY USA; 2 Department of Biostatistics and Computational Biology, University of Rochester Medical Center, Rochester, USA; 3 Miner Library, University of Rochester Medical Center, Rochester, NY USA; 4 Pediatrics, King Fahd Medical City, Riyadh, Saudi Arabia

**Keywords:** *Lactiplantibacillus plantarum*, Health benefits, Oral health, Gastroenterology, Cardiovascular, Infectious diseases

## Abstract

To ensure effective administration of probiotics in clinical practice, it is crucial to comprehend the specific strains and their association with human health. Therefore, we conducted a systematic review and meta-analysis to evaluate the scientific evidence on the impact of *Lactiplantibacillus plantarum* probiotic consumption on human health. Out of 11,831 records, 135 studies were assessed qualitatively, and 18 studies were included in the meta-analysis. This systematic review demonstrated that probiotic supplementation with *L. plantarum*, either alone or in combination, can significantly improve outcomes for patients with specific medical conditions. Meta-analysis revealed notable benefits in periodontal health, evidenced by reduced pocket depth and bleeding on probing (*p* < 0.001); in gastroenterological health, marked by significant reductions in abdominal pain (*p* < 0.001); and in infectious disease, through a reduction in C-reactive protein levels (*p* < 0.001). Cardiovascular benefits included lowered total cholesterol and low-density lipoprotein cholesterol in the *L. plantarum* intervention group (*p* < 0.05). Our study’s clinical significance highlights the importance of considering probiotic strain and their application to specific diseases when planning future studies and clinical interventions, emphasizing the need for further research in this area.

**Supplementary Information:**

The online version contains supplementary material available at 10.1007/s12602-024-10287-3.

## Introduction

According to the World Health Organization, probiotics are “living microorganisms which, when administered in adequate amounts, confer a health benefit on the host” [1]. Probiotic consumption’s favorable effect on general health has been recognized and gained popularity among patients [1]. In 2017, a survey conducted among 965 patients at a tertiary medical center in California, USA, reported that the use of probiotics among patients was as high as 55%, including food products and supplements [2].

However, the results from recent research have supported the concept that probiotics are not equally effective [3]. Clinical trials have investigated the health benefits of many single or multi-strain probiotic mixtures to meet patients’ health needs [4]. Yet, due to the manufacturing processes, efficacy by different strains, quality control, and the differences in the probiotics' mechanism of action [5], it is challenging to reach a consensus on establishing recommendations on which probiotic strain-specific should be used for a particular disease condition [6, 7].

Although the health professions have begun to promote probiotics, particularly in children and high-risk populations, with the increased belief in the beneficial effects of probiotics added to food on human health, there is an international absence of consensus on the methodology to evaluate probiotics’ efficacy and safety [1]. In 2010, a genomic analysis reported the differences in strain-specific efficacy in more detail [8, 9]. Experts and international probiotic guidelines have started strongly recommending the use of strain designation in clinical trial outcome reporting. This approach facilitates the determination of strain-specific efficacy, which recent research has shown to be significant. Studies have investigated both strain-specificity and disease-specificity of probiotic efficacy, revealing strong evidence that the efficacy of probiotics varies depending on the strain and the disease being targeted [3]. Still, these recommendations were not followed regularly [6, 10–12].

Furthermore, from the regulatory standpoint, the US FDA and the European Food Safety (EFSA) did not attribute the administration of probiotics to preventing or treating any disease. They did not approve any health claims related to the administration of probiotics [13]. On the other hand, Health Canada has approved multi-strain probiotics such as *Lactiplantibacillus plantarum* (SD5209) and a single strain of *Bifidobacterium animalis* spp. *lactis* LAFTI B94 to be IBS symptom relief products [13].

Both *Bifidobacterium* and *Lactobacilli* are the most widespread probiotic bacterial strains used in dietary supplements or various functional foods [4, 5]. *Lactobacillus* spp. is considered the main lactic acid bacteria diverse group, isolated from various ecological niches, and many trials discussed their physiological and genetic differences [14]. Some species of *Lactobacillus* are only observed in limited ecological niches, such as *Lactobacillus debrueckii* or *Lactobacillus rhamnous* [15]*.* On the contrary, *L. plantarum* is found in many products such as dairy [14], meat [16], fermented foods [17], and vegetables [18]. Besides the food industry application*, L. plantarum* has many pharmaceutical uses with no side effects and a significant contribution to human medicine [19]. Recently, *L. plantarum* has been implemented in clinical fields to remedy various illnesses, including but not limited to Alzheimer’s disease, Parkinson’s disease, diabetes, obesity, cancer, hypertension, urinogenital complications, and liver disorders [20–24]. In particular, *L. plantarum* strains were reported to produce pro-inflammatory cytokines in the intestinal epithelial cells [25] and reduce kidney stones [26].

Assessing the latest scientific evidence is essential to better understand the beneficial effects and characteristics of strain-specific probiotics and their impact on one illness. Therefore, the systematic review aims to comprehensively assess the latest evidence from randomized clinical trials regarding the health benefits of *L. plantarum.*

## Methods

This systematic review followed the PRISMA guidelines [27], and the protocol was registered in the PROSPERO [CRD42023414184] (https://www.crd.york.ac.uk/prospero/).

### Search Methods

Database searches were conducted in March 2022 to identify published studies on the health benefits of *L. plantarum.* A medical reference librarian developed the search strategies and retrieved citations from the following databases: PubMed, Embase, Web of Science (all Databases), Cochrane Library-Central Register of Controlled Trials. A second librarian proofread the strategies. See Appendix 1 for a thorough description of the search strategies employed.

### Inclusion and Exclusion Criteria

This systematic review included randomized controlled trials that examined the health benefits outcomes of the intervention with *L. planatrum* in a population with a pre-existing medical health condition. Two trained independent reviewers completed the article selection following the inclusion/exclusion criteria. Disagreements were resolved by consensus between the two reviewers or by the third reviewer.

#### Inclusion Criteria

- Types of participants: individuals (children and adults) with pre-existing medical conditions.
- Types of intervention: consumption of *L. plantarum* probiotic
- Types of comparison:
  - Individuals (children and adults) who received *L. plantarum* probiotics.
  - Individuals (children and adults) who did not receive *L. plantarum* probiotic (placebo), with or without receiving the standard treatment care protocol for their health condition.
- Types of outcomes: health benefits (e.g., mental health, upper respiratory system, oral health, gastrointestinal health)
- Types of studies: randomized controlled trials.

#### Exclusion Criteria

In vitro studies, animal studies, papers with abstracts only, literature reviews, letters to the editor, editorials, patient handouts, case reports or case series, case–control studies, cross-sectional studies, retrospective cohort studies, and publication in a non-English language.

### Data Extraction

Descriptive data were obtained, including clinical and methodological factors such as country of origin, study design, clinical sample source, measurement interval, age of subjects, medical condition, outcome measures, and results from the statistical analysis.

#### Qualitative Assessment and Quantitative Analysis

The selected articles’ methodological validities were assessed by the Cochrane risk-of-bias tool for randomized trials (RoB 2) [28]. Articles were scaled for the following bias domains: randomization process, deviation from intended intervention, missing outcome data, measurement of the outcome, selection of the reported result, and overall bias. The articles selected for quantitative analysis have been conducted via meta-analysis using statistical software (OpenMeta[Analyst], developed by Brown University). All results for meta-analysis were subject to double data entry. Effect sizes expressed as weighted mean differences (for continuous data) and their 95% confidence intervals were calculated for each specific analysis. Heterogeneity was assessed statistically using the standard chi-square and explored using subgroup analyses based on this review's different quantitative study designs. Where statistical pooling is not possible, the findings are presented in narrative form, including tables and figures to aid in data presentation where appropriate.

## Results

**Fig. 1:**
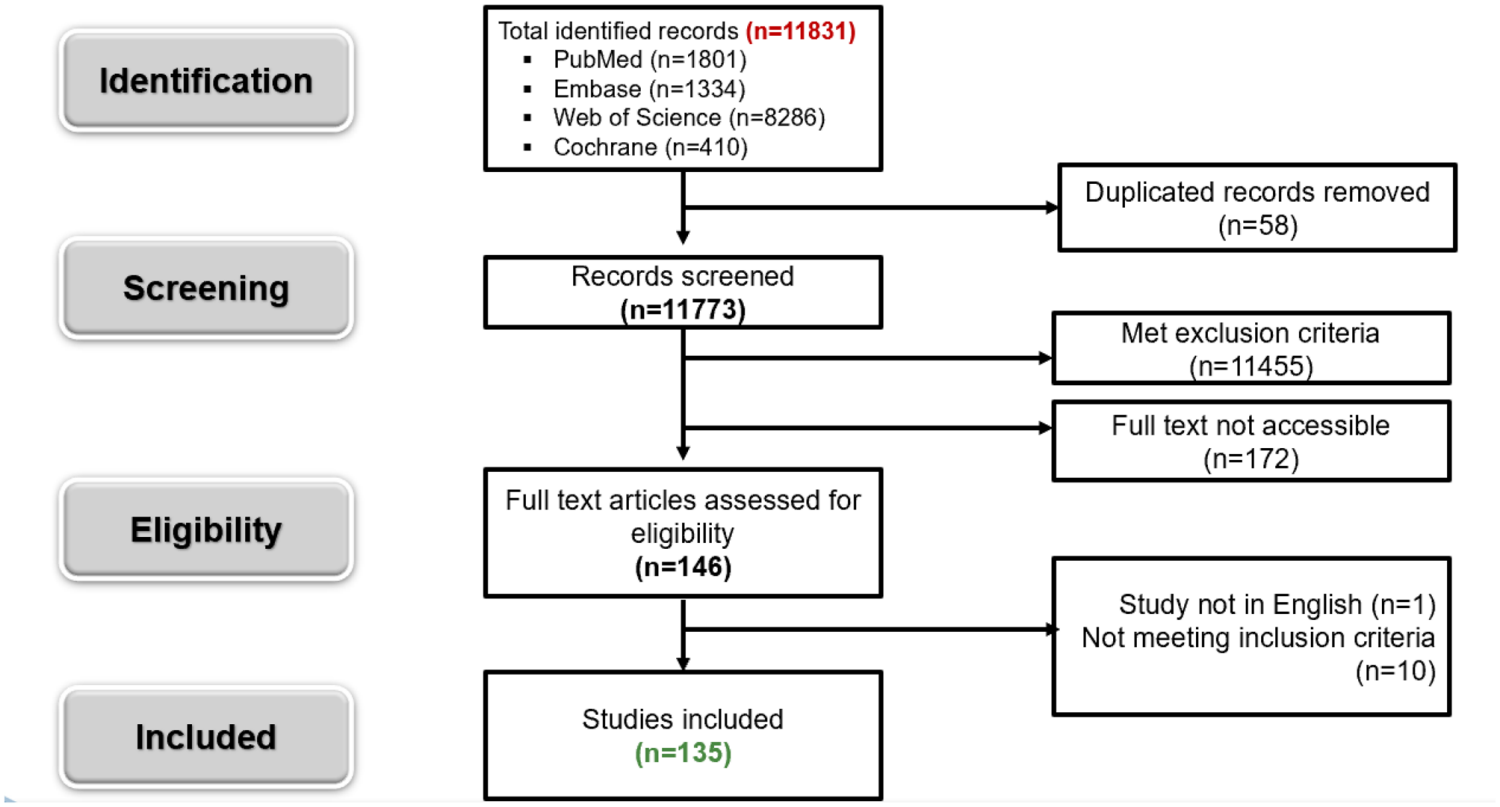
Flow diagram of study identification

The literature analysis identified a total of 11,831 records from the database search. A total of 58 duplicated references were removed. Of the remaining 11,775 records, 11,455 were excluded after the title and abstract screening, and an additional 172 were excluded since the full text was not accessible. The remaining 148 studies proceeded to the full-text review; 11 studies were further eliminated based on exclusion criteria, and 135 articles were chosen for qualitative assessment, see Fig. 1 for study flow.

The 4-phase Preferred Reporting Items for Systematic Reviews and Meta-Analyses (PRISMA) flow diagram was used to determine the number of studies identified, screened, eligible, and included in the systematic review and meta-analysis (http://www.prisma-statement.org).

### Study Characteristics

The characteristics of studies included in the qualitative review are summarized in Supplemental Tables S1–S16. A total of 135 studies are categorized into the following subgroups: Oral health (7 studies in Table S1), Endocrinology (13 studies in Table S2), Cardiovascular and Critical care (5 studies in Table S3), Dermatology (6 studies in Table S4), Gastroenterology (47 studies in Table S5), Obstetrics and Gynecology (3 studies in Table S6), Hematology (2 studies in Table S7), Allergy (1 study in Table S8), Neonatology (1 study in Table S9), Pulmonary (1 study in Table S10), Nephrology (2 studies in Table S11), Mental health and behavioral disorder (16 studies in Table S12), Obesity (9 studies in Table S13); Oncology and Surgery (12 studies in Table S14), Infectious disease (8 studies in Table S15), and Hepatology (2 studies in Table S16).

### Impact of Consuming Probiotics Including *L. Plantarum* on Systemic Diseases

#### Oral Health Benefits

Oral health benefits were seen among subjects who received *L. plantarum* probiotics compared to those who did not. Two studies indicated that the application of *L. plantarum* was associated with improved periodontal status reflected by reduced pocket depth and bleeding on probing [29, 30]*.* Similarly, *L. plantarum* intervention was associated with pain reduction after third molar extraction [31]. Whether *L. plantarum* could reduce *Streptococcus mutans* oral carriage was inconclusive [32, 33].

#### Endocrine System

Intriguingly, consumption of *L. plantarum* had a substantial impact on diabetic patients by lowering HbA1c values [34, 35], as well as reducing diabetic patients’ systolic and diastolic blood pressure [36–38]. Several studies show that the supplementation of *L. plantarum* to type 2 diabetic patients with nephropathy improved urinary albumin-creatinine ratio, serum creatinine, and glomerular function [39–41]. However, *L. plantarum* did not appear to impact fasting blood glucose levels [42, 43].

#### Cardiovascular System and Critical Care

In most studies reported, *L. plantarum* intervention reduced total cholesterol (TC), low-density lipoprotein-cholesterol (LDL-C), oxidized LDL-cholesterol (OX-LDL), and triglyceride (TG) levels while raising high-density lipoprotein cholesterol (HDL-C) levels in patients with hypercholesterolemia. *L. plantarum* intervention improved serum lipid profile measures [44–46]. Although *L. plantarum* administration to critically ill patients is safe, it does not improve patient outcomes [47].

#### Dermatology

Three studies suggested that *L. plantarum* supplementation may reduce the clinical symptoms of atopic dermatitis [48–50]. Kim et al. showed that administering *L. plantarum* could decrease the clinical severity of acne vulgaris [51]. *L. plantarum* showed an excellent anti-aging effect by increasing skin moisture and reducing facial wrinkles [52]. Yang et al. found no significant differences in the clinical severity of atopic dermatitis between the *L. plantarum* and control group [53].

#### Gastroenterology

Probiotic supplementation with *L. plantarum* could potentially improve outcomes in patients with irritable bowel syndrome (IBS) symptoms. Fewer studies reported a significant reduction in pathogenic gut microbiota and an increase in the relative abundance of *Bifidobacterium* and butyric acid-producing bacterial genera [54–56]. However, two articles observed unfavorable effects of *L. plantarum* administration in patients with IBS symptoms [57, 58], and two studies showed no significant difference between the intervention and control groups [59, 60]. All studies reported more practical benefits of adjunctive *probiotic L. plantarum* supplementation combined with standard *H. pylori* eradication therapy by increasing eradication rates and reducing antibiotic side effects [61–63], except one [64]. *L. plantarum* supplementation also enhanced immune response in patients with gastrointestinal disorders such as acute infections, ulcerative colitis, infectious diarrhea, and celiac disease, as reported in some studies [54, 65–67].

#### Obstetrics and Gynecology

Two studies indicated that using *L. plantarum* improved bone health markers in menopausal women [68, 69]. Tomusiak, et al. reported that it was safe to use probiotics with *L. plantarum* to reestablish a healthy vaginal microbiome gradually based on the analysis of types and occurrence of adverse events [63].

#### Hematology

The available data suggested that administering *L. plantarum* and iron to female adults would improve iron status more quickly than administering iron alone, even though the results did not achieve statistical significance [70]. While *L. plantarum* administration did not improve treatment outcomes in children, it was equally effective as ferrous alone in addressing iron insufficiency when combined with low-dose ferrous [71].

#### Allergy

Although only one study was included, consuming *L. plantarum* had positive clinical effects on seasonal allergy illness symptoms [72].

#### Neonatology

The combined use of probiotics, including *L. plantarum* with oligosaccharides and lactoferrin, was associated with decreased IL-10 levels, but no change was observed in the other cytokines, such as interleukin IL-5 and IL-17A [73].

#### Pulmonology

Only one study in this systematic review met the inclusion criteria, which indicated that in a population with chronic obstructive pulmonary disease (COPD), short-term consumption of a multispecies probiotic that included *L. plantarum* had little impact on the intestinal microbiota. Administration of *L. plantarum* did not restore the microbiota imbalance, and no decrease in diarrhea-like bowel movements was observed as a result [74].

#### Nephrology

Kidney diseases are categorized as either chronic or acute. Regardless of the initial cause, inflammation, and immune system activation are a common underlying mechanism in both chronic and acute renal disorders. Probiotic administration of *L. plantarum* affected certain inflammatory markers, such as reducing serum levels of tumor necrosis factor-alpha TNF-α and increasing serum levels of anti-inflammatory cytokines IL-10 compared to controls [75, 76].

#### Mental Health and Neurology

*L. plantarum* Intervention was associated with more remarkable improvement in cognitive function in patients with cognitive impairment [77–81]. Administering *L. plantarum* in patients with autism spectrum disorder (ASD) may improve behavioral or social communication traits and body and object use [82, 83]. Few studies have indicated that consuming *L. plantarum* may decrease fatigue, improve sleep quality, and reduce migraine severity [77, 84]. Most studies suggested that daily administration of *L. plantarum* may decrease anxiety symptoms [78, 80, 85, 86]. Most studies’ findings favor that *L. plantarum* administration could decrease depression symptoms [77–79, 85, 87]. Some studies point to the ability of *L. plantarum* to help in reducing stress [79, 85].

#### Obesity

Most studies showed that probiotics with *L. plantarum* reduced BMI, body weight, and waist-to-hip ratio (WHR) in overweight/obese adults [42, 88–90], and suggested that intervention of probiotics with *L. plantarum* could reduce total serum cholesterol in overweight subjects [88, 91–93]. Furthermore, only one study showed a significant increase in HDL cholesterol after administering *L. plantarum* [90]. One study showed that *L. plantarum* had preventive potential against CMD (cardiometabolic diseases) in overweight patients [91].

#### Oncology and Surgery

Whether *L. plantarum* intervention could reduce the incidence of postoperative complications is inconclusive from eligible studies [94, 95]. Furthermore, it was indecisive whether the intervention shortened the length of stay at the hospital [96, 97]. All studies showed no differences in the quality of life between intervention and control groups [98, 99].

#### Infectious Disease

No significant differences in serum CD4 + count were found between the control and intervention groups [100]. As for the C-reactive protein (CRP), two studies showed no significant differences between the control and the intervention [101, 102]. The other two significantly reduced CRP in the intervention group [103, 104]. All studies showed improved/no new infiltrations on subsequent chest X-rays [105, 106]. ICU stays were found to be shorter in both studies with the intervention [103, 104]. Both studies have shown significantly lowered IL-6 levels in the intervention group compared to the control [101, 104]. It is inconclusive whether the intervention with *L. plantarum* could improve the mortality rate caused by an infectious disease [101, 104].

#### Hepatology

Alanine transaminase (ALT) levels were not affected by the intervention of probiotics with *L. plantarum* in two studies included [107, 108]

### Quality and Risks

**Fig. 2:**
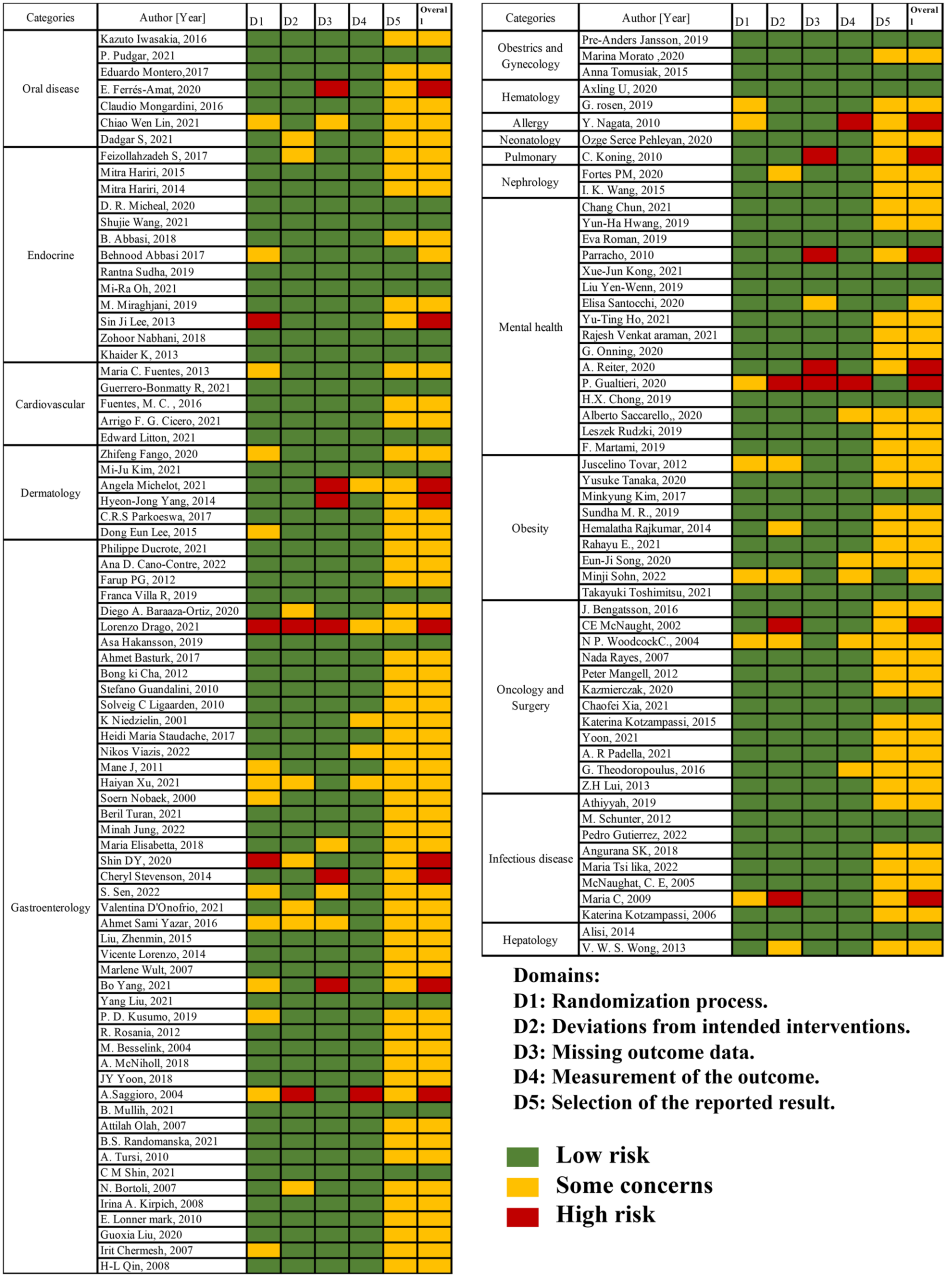
Summary of quality and risk of bias assessment using the Cochrane Collaboration’s tool for assessing risk of bias in randomized trials. The quality of the selected articles was assessed using the Cochrane risk-of-bias tool for randomized trials (RoB 2) [28]

Quality and risk of bias for the randomized controlled trials were assessed and shown in Fig. 2.

### Meta-Analysis

Studies that had the same comparison and outcome measures were included in the meta-analysis. The outcomes that were assessed between groups who received the *L. planatrum* and the control groups included (a) Pocket Depth (PD) and Bleeding on Probing (BOP) reduction; (b) abdominal pain score reduction; (c) C-reactive protein level reduction; (d) body mass index (BMI) reduction; (e) low-density lipoprotein-cholesterol (LDL-C) level reduction; and (f) total cholesterol (TC) level reduction.

**Fig. 3:**
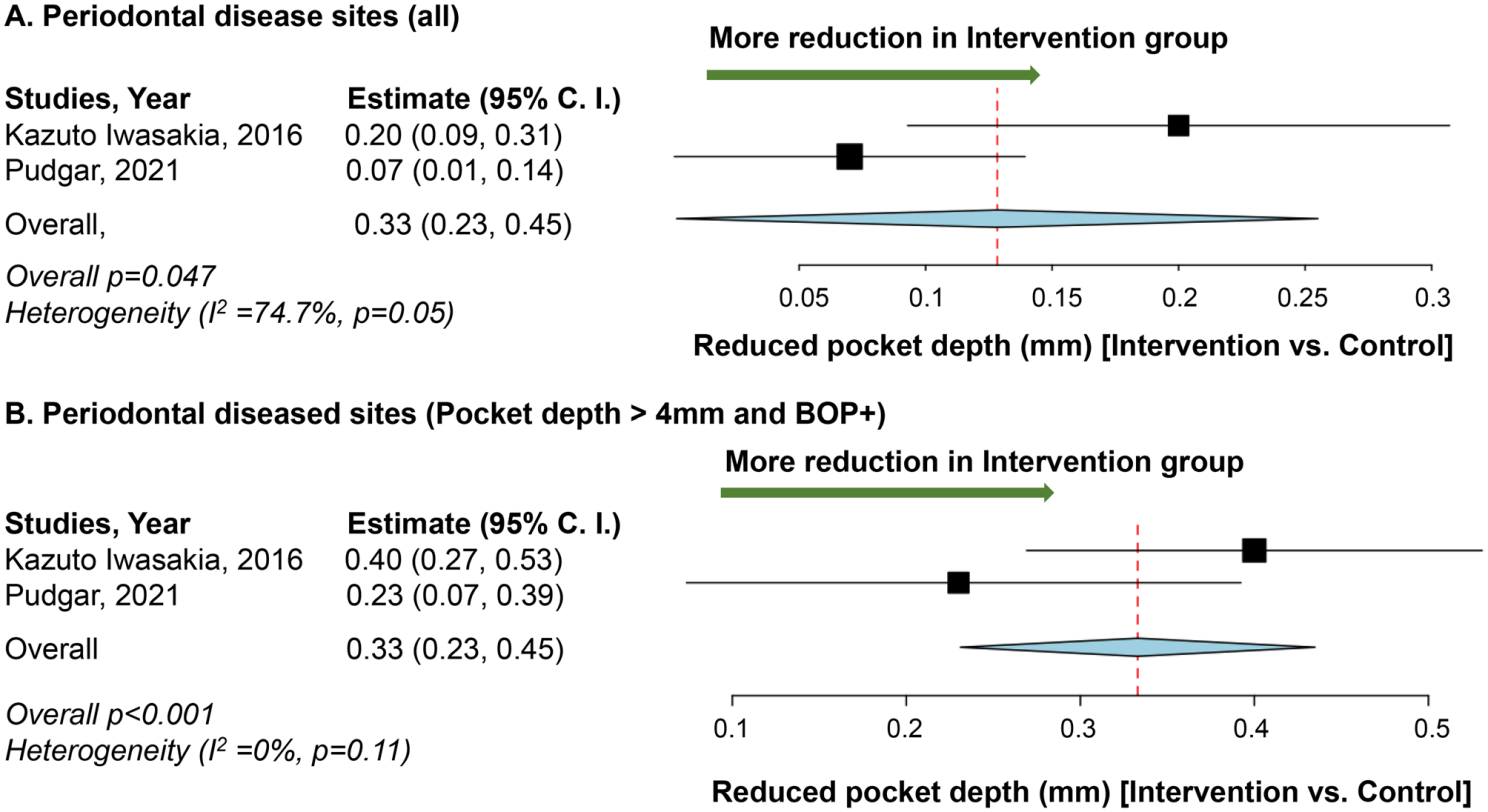
Impact of *L. plantarum* on periodontal health. **A** Mean difference in reduction of pocket depth between the *L. plantarum* intervention and the control groups among all periodontal disease sites. **B** Mean difference of reduction of pocket depth between the *L. plantarum* intervention and the control groups among periodontal disease sites that had a pocket depth of more than 4 mm and with bleeding on probing. Study heterogeneity (*I*^2^) and the related *p* value were calculated using the continuous random effect methods. The mean difference, 95% confidence interval (C.I.) of each study included in the meta-analyses and forest plots of comparisons

Two studies indicated that the application of *L. plantarum* was associated with improved periodontal status measured by PD and BOP [29, 30]. Meta-analysis indicated that the intervention with *L. plantarum* had a higher reduction of PD compared to the control group in the analysis of all sites (*p* = 0.047) and periodontally diseased sites only (*p* < 0.001) (Fig. 3). The group with *L. plantarum* consumption showed a higher PD reduction than the control group, with statistical significance for all teeth sites. The higher reduction of PD is 0.128 mm. When only periodontally diseased sites were included, these sites had a pocket depth of more than 4mm. With BOP, the group with *L. plantarum* consumption had a higher PD reduction than the control group with statistical significance. The reduction of PD that was more in the intervention group was 0.33 mm.

**Fig. 4:**
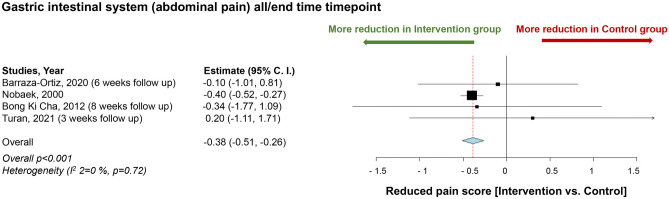
Impact of *L. plantarum* on gastrointestinal health. Mean difference of reduction of abdominal pain score between the *L. plantarum* intervention and the control groups at all time points. Study heterogeneity (*I*^2^) and the related *p* value were calculated using the continuous random effect methods. The mean difference, 95% confidence interval (C.I.) of each study included in the meta-analyses and forest plots of comparisons

Several studies have reported a significant improvement in abdominal pain symptoms with the administration of *L. plantarum* compared to control groups. A meta-analysis was conducted, including four studies [109–112], which showed that intervention with *L. plantarum* resulted in a statistically significant improvement in the VAS scale for abdominal pain compared to the control groups (*p* < 0.001) (Fig. 4).

**Fig. 5:**
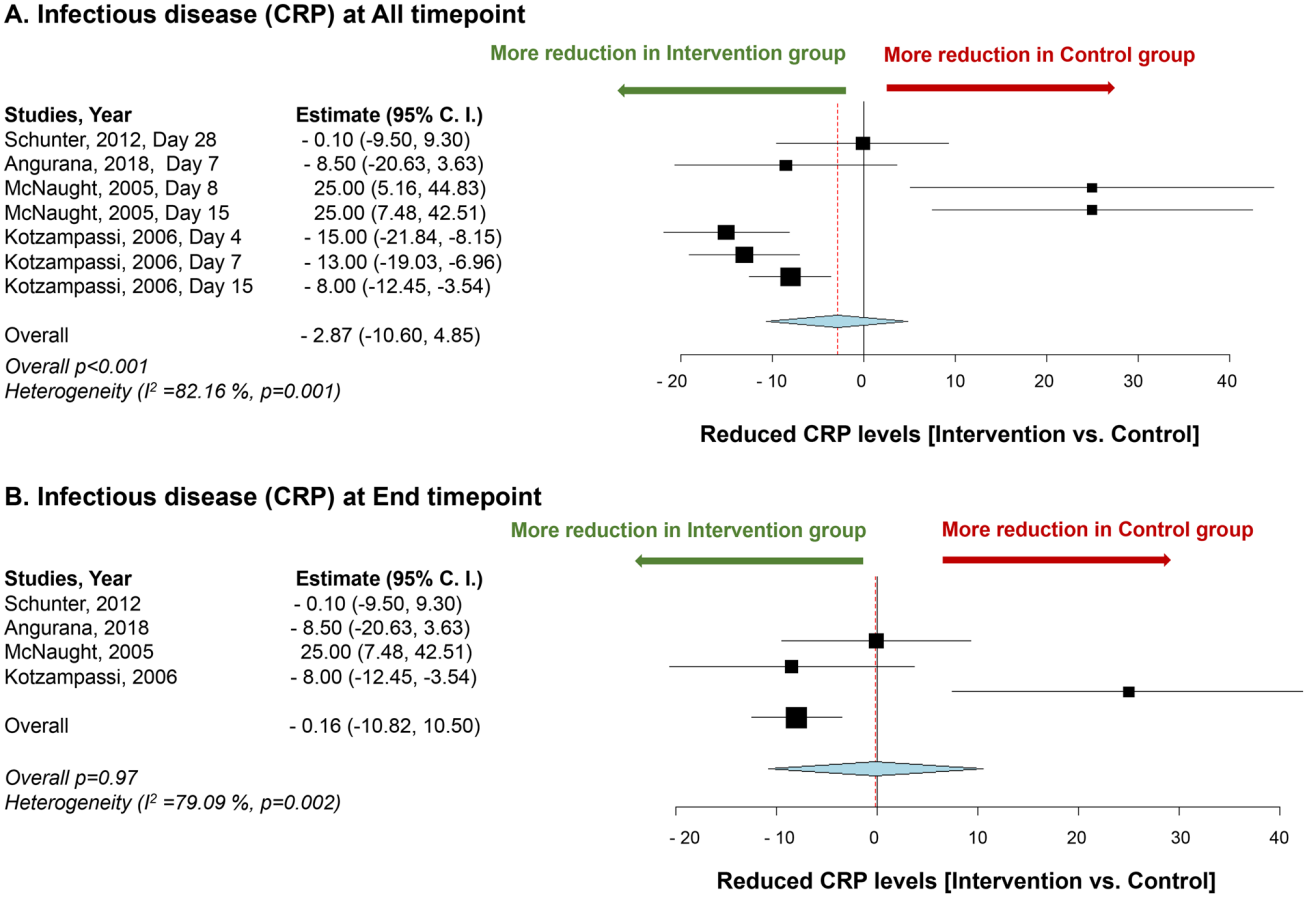
Impact of *L. plantarum* on Infectious disease. **A** Mean difference of reduction of C-reactive protein between the *L. plantarum* intervention and the control groups at all time points. **B** Mean difference of reduction of C-reactive protein between the *L. plantarum* intervention and the control groups at end time point. Study heterogeneity (*I*^2^) and the related *p* value were calculated using the continuous random effect methods. The mean difference, 95% confidence interval (C.I.) of each study included in the meta-analyses and forest plots of comparisons

In two studies, the application of *L. plantarum* showed a significant reduction in C-reactive protein (CRP) levels compared to the control group [103, 104], while the other two studies did not show a significant difference between the control and intervention groups [101, 102]. However, meta-analysis showed no significant difference in CRP levels between the groups (*p* = 0.97) (Fig. 5).

**Fig. 6:**
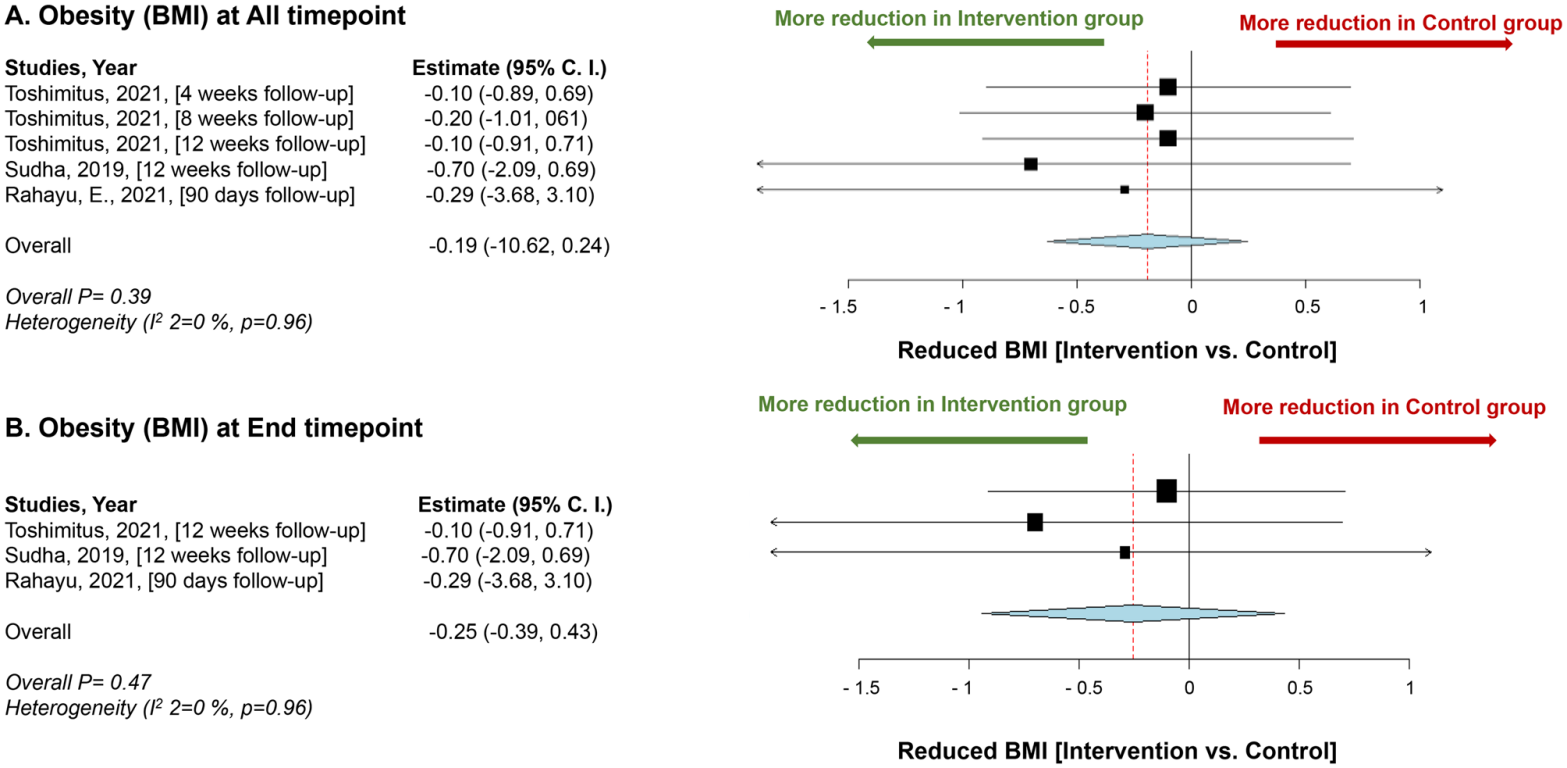
Impact of *L. plantarum* on obesity (body mass index). **A** Mean difference of reduction of Body Mass Index between the *L. plantarum* intervention and the control groups at all time points. **B** Mean of body mass index reduction between the *L. plantarum* intervention and the control groups at the end time point. Study heterogeneity (*I*^2^) and the related *p* value were calculated using the continuous random effect methods. The mean difference, 95% confidence interval (C.I.) of each study included in the meta-analyses and forest plots of comparisons

Although many studies have reported a significant reduction in BMI with the use of probiotics, our meta-analysis, which included three studies [89, 42, 90], did not show significant results (*p* = 0.96) (Fig. 6).

**Fig. 7:**
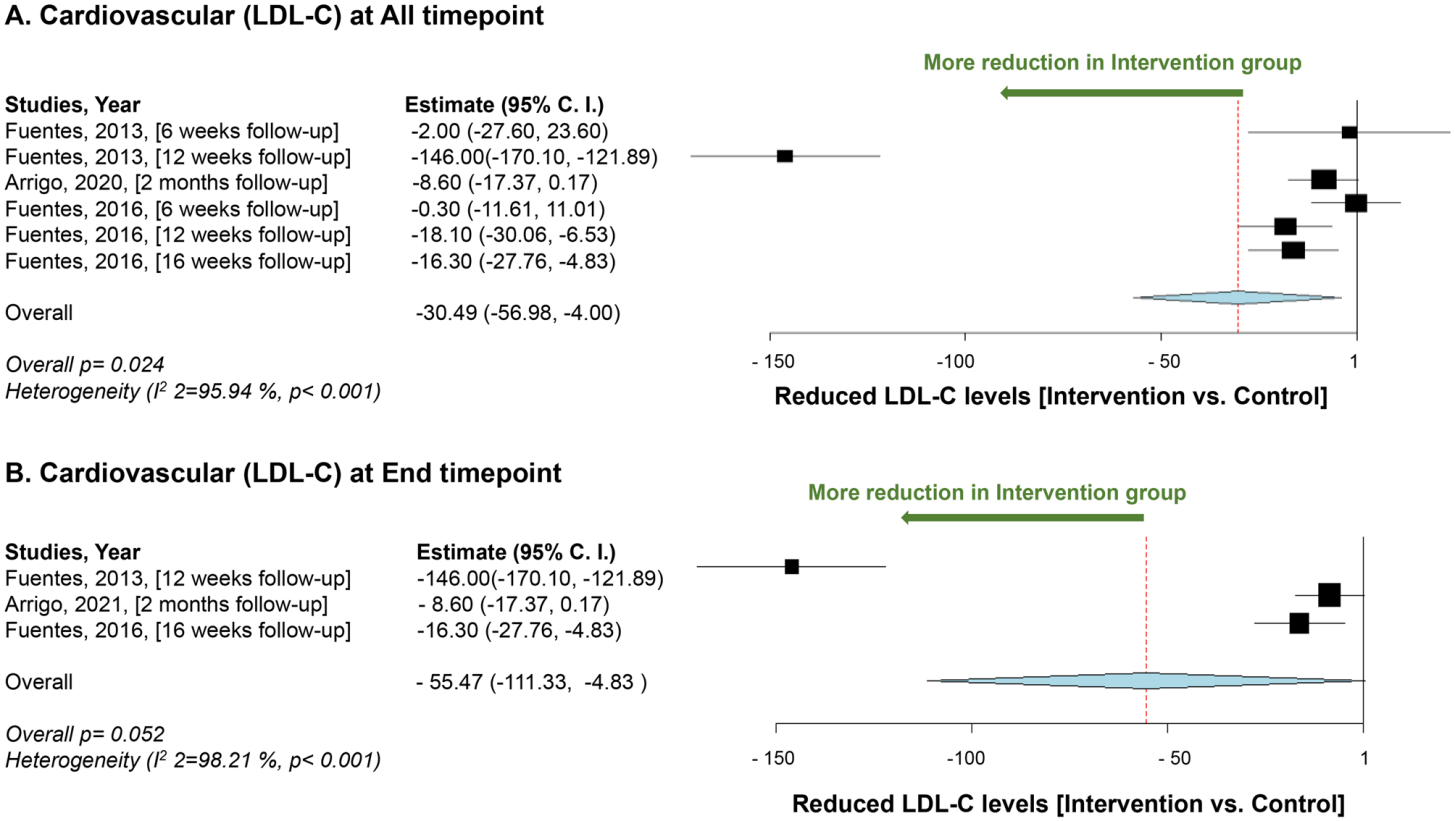
Meta-analysis results for cardiovascular (low-density lipoprotein-cholesterol). **A** Mean difference in reduction of low-density lipoprotein-cholesterol levels between the *L. plantarum* intervention and the control groups at all time points. **B** Mean of low-density lipoprotein-cholesterol levels reduction between the *L. plantarum* intervention and the control groups at the end timepoint. Study heterogeneity (*I*^2^) and the related *p* value were calculated using the continuous random effect methods. The mean difference, 95% confidence interval (C.I.) of each study included in the meta-analyses and forest plots of comparisons

**Fig. 8:**
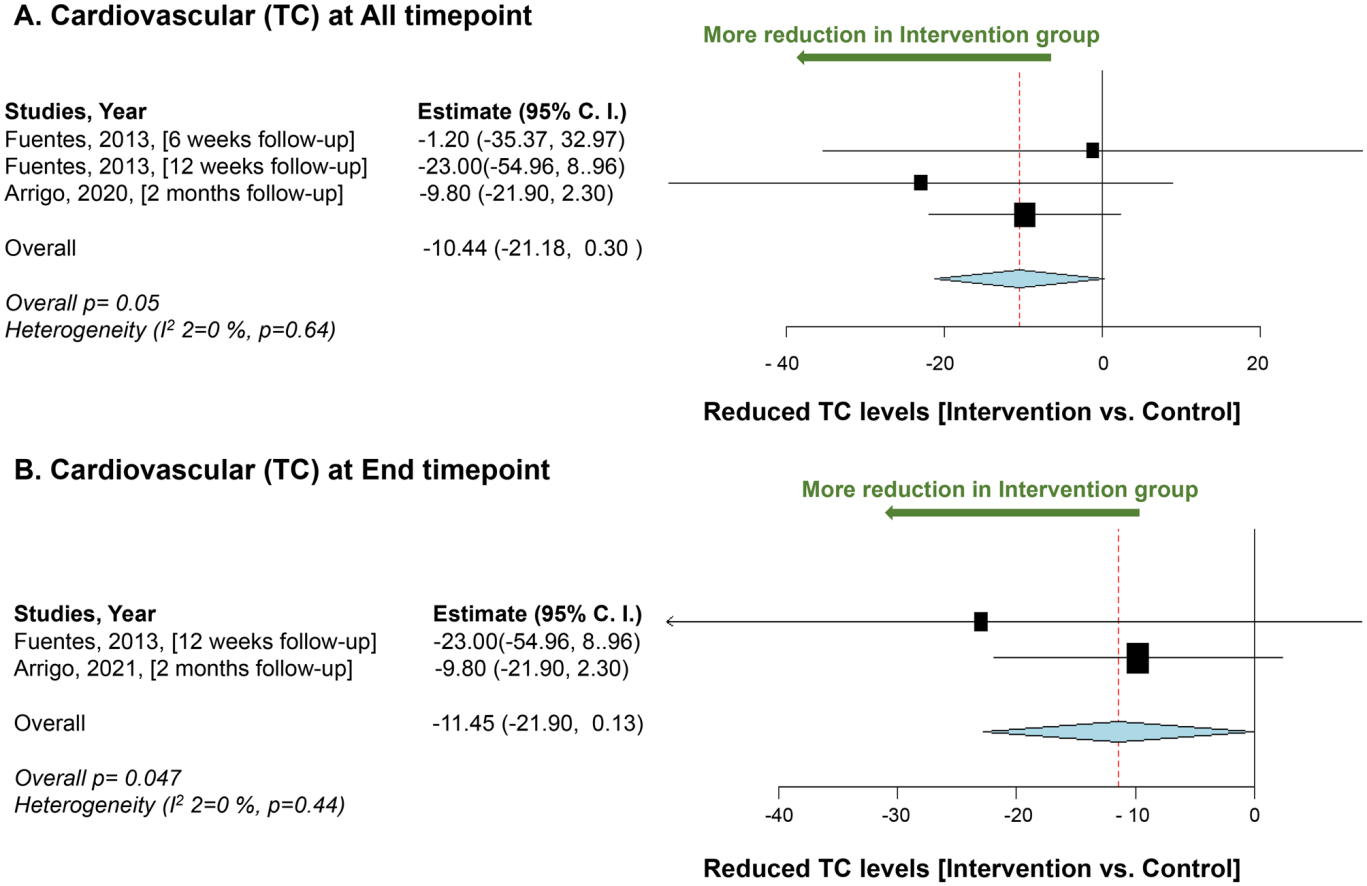
Meta-analysis results for cardiovascular (total cholesterol levels). **A** Mean difference in reduction of total cholesterol (TC) levels between the *L. plantarum* intervention and the control groups at all time points. **B** Mean of Total cholesterol levels reduction between the *L. plantarum* intervention and the control groups at the end time point. Study heterogeneity (*I*^2^) and the related* p* value were calculated using the continuous random effect methods. The mean difference, 95% confidence interval (C.I.) of each study included in the meta-analyses and forest plots of comparisons

Our meta-analysis of the cardiovascular system includes five studies, of which three studies analyze the data for LDL-C levels and two studies for TC levels. LDL-C levels showed marginal significance (*p* = 0.05) in favor of intervention [44, 46, 113](Fig. 7). While TC level showed significant reduction in the intervention groups (*p* = 0.04) (Fig. 8) [44, 46].

## Discussions

Despite international standards, many published meta-analyses and reviews erroneously group probiotic strains or species together and fail to disclose strain identities [114]. This review focuses on the *L. plantarum* strain specifically and provides a summary of its health benefits by comparing its efficacy as a treatment or adjunctive treatment in individuals with pre-existing health conditions to that of a control group. In addition, the review assessed the effectiveness of *L. plantarum* on each health condition separately to determine disease-specificity. Experts believe that certain probiotic strains may effectively treat certain diseases and demonstrate clinical efficacy for that disease while not being effective for a different disorder. The results of this study provide support for the idea that each probiotic strain has a specific disease-specific therapeutic benefit [3].

Furthermore, supports the concept that *L. plantarum* specious may demonstrate a superior outcome in treating some disease [115] or as adjunctive therapy with the standard treatment [87]. Our study indicates that *L. plantarum* can be safely used with most therapeutic interventions and drugs. These results align with other studies that indicated the safety uses of *L. plantarum* [19, 116].

It is noteworthy that only two trials using the same probiotic strain *L. plantarum* MF1298 in our study reported direct adverse effects on gastrointestinal symptoms [57, 58], while another trial reported a potential indirect effect in neonates by reducing IL-10 levels, which could increase the incidence of NEC [73]. These findings highlight the importance of carefully monitoring for potential adverse effects when using probiotics, particularly in vulnerable populations such as neonates, and suggest the need for further investigation into the safety and efficacy of probiotics in these populations.

### *L. Plantarum* Consumption: Benefits or Limitations on Oral Health

Our study examined the existing literature on the use of *L. plantarum* in oral health interventions. Numerous studies have reported promising benefits of using *L. plantarum* in various aspects of oral health, including pain reduction after third molar extraction and improved periodontal status. One study demonstrated a significant impact of *L. plantarum* in reducing *S. mutans* carriage, which is consistent with recent findings [115]. However, another study failed to show significant results on the ability of *L. plantarum* to reduce *S. mutans* carriage. Despite these conflicting results, our meta-analysis confirmed a higher reduction in pocket depth with the use of *L. plantarum* compared to other parameters.

### *L. Plantarum* Consumption: Benefits or Limitations on Endocrine System

The expert panel of the American Diabetes Association has predicted that up to 70% of prediabetic individuals will eventually be diagnosed with type 2 diabetes [117]. Therefore, it is crucial to encourage individuals with diabetes and prediabetes to adopt healthy lifestyles to prevent or delay the onset of diabetes-related complications. In recent clinical trials, the administration of several multi-strain probiotic formulations over 12-week periods led to significant decreases in HbA1c levels among individuals with type 2 diabetes [118, 119]. A study using multi-strain probiotics including *L. plantarum* supports these findings [34]. Conversely, in two prior clinical trials using single-strain probiotics (*Lacticaseibacillus casei* or *Limosilactobacillus reuteri*), those strains were unable to reduce HbA1c levels [120, 121].

Interestingly, our study findings suggested that *L. plantarum* as a single strain could reduce HbA1c levels alone. Moreover, they found no variations in the diversity or taxonomic composition of the human fecal microbiome. These results might suggest that microbial products, instead of differences in microbiota composition, regulate glycemic control in humans [35].

### *L. Plantarum* Consumption: Benefits or Limitations on Cardiovascular System and Critical Care

Being the leading cause of death worldwide, cardiovascular diseases (CVDs) account for 31% of all fatalities, according to the World Health Organization. Convincing data indicate that high levels of low-density lipoprotein cholesterol (LDL-C) are a critical modifiable risk factor for CVD [122], making LDL-C a significant target for risk reduction [123]. Our study results support the findings of a meta-analysis that demonstrated the significant impact of some species of *L. plantarum* and *L. reuteri* on blood cholesterol levels, such as LDL-C and total cholesterol (TC) [124].

The findings of our meta-analysis showed a marginal significance in reducing LDL-C levels, possibly due to the high heterogeneity among the included studies (*I*^2^ = 98.21%, *p* < 0.001). This highlights the need for guidelines for future clinical trials to ensure more homogeneous studies, which would yield more reliable results for drawing conclusive conclusions. Additionally, although the meta-analysis included only two studies, the results showed a significant reduction in TC levels in the intervention group. It is worth noting that these two studies were heterogeneous, which adds to the reliability of the data. However, more clinical trials are still needed to obtain conclusive results and draw evidence-based conclusions.

### *L. Plantarum* Consumption: Benefits or Limitations on Dermatology

Atopic dermatitis (AD) is a chronic, multifactorial inflammatory skin condition affecting adults and children at high rates globally [125]. There is ongoing scientific debate about the relationship between skin and gut bacteria [126, 127]. Our study observed a statistically significant decrease in the AD index (SCORAD score) in three studies involving individuals with mild-to-moderate AD who received probiotics containing *L. plantarum*. This reduction in SCORAD score is consistent with previous research suggesting that probiotics may have a beneficial effect in reducing AD symptoms [127–129].

### *L. Plantarum* Consumption: Benefits or Limitations on Gastroenterology

Probiotics have been demonstrated to affect IBS in several trials [117] *L. plantarum* 299v has been found to alleviate abdominal pain in IBS patients [110]. However, it is important to consider the strain-specific efficacy of probiotics, as different strains may have varying outcomes. Among 22 strains of lactobacilli isolated from fermented food products, *L. plantarum* MF1298 was found to have the best in vitro probiotic characteristics [130, 131], suggesting it is a potential probiotic strain. However, contrary to these advantageous characteristics, our study found that in randomized placebo-controlled crossover research, *L. plantarum* MF1298 had a negative impact on symptoms of IBS patients [57, 58], which is surprising as most studies report their findings as either significant or non-significant. It is worth noting that none of the other studies included in our analysis reported direct adverse effects, except for those specifically examining *L. plantarum* MF1298. The standard IBS score increased slightly but statistically significantly due to this adverse effect, as determined by factors such as “abdominal pain/discomfort,” “urgency,” and “stool frequency/consistency” based on patients’ subjective perception of GI function during a therapy vs. a control period. Overall, administration of *L. plantarum* may be considered a safe and well-tolerated adjunctive approach in alleviating IBS symptoms and certain GI symptoms, while improving IBS-QoL compared to a routine regimen alone. However, further investigation is needed to determine its effects on fecal microbiome alteration, bacterial diversity, and serum cytokines.

The meta-analysis showed promising results for the use of *L. plantarum* in reducing abdominal pain in patients with IBS symptoms. And with a low heterogeneity, we can comfortably say that these results could be conclusive.

### *L. Plantarum* Consumption: Benefits or Limitations on Obstetrics and Gynecology

Fragility fractures resulting from osteoporosis pose a significant clinical burden and are associated with increased mortality [132]. Women, in particular, are at higher risk for fractures due to lower bone mass and accelerated bone loss during and after menopause [133]. Our study findings suggest that the administration of probiotics containing *L. plantarum* to menopausal women improves their bone health markers. These results are consistent with previous experimental studies that have shown a bone-protective effect of probiotic therapies in animal models of ovariectomy-induced bone loss [134–136]. Further long-term trials are warranted to investigate if the bone-protective benefits of *L. plantarum* intensify with continued administration.

### *L. Plantarum* Consumption: Benefits or Limitations on Hematologic diseases

Iron deficiency anemia (IDA) is a widespread condition affecting millions of individuals, including children, pregnant women, and non-pregnant women [137]. In a study by Axling et al., both groups, one receiving Lp299v plus 20 mg of iron and the other receiving only 20 mg of iron, showed improvement in their iron status over the 12-week study period [70]. However, the difference between the groups did not reach statistical significance, despite a higher increase in plasma ferritin (70%) in the Lp299v group compared to the control group (42%). Nevertheless, after the initial four weeks, the LpFe group (intervention group) tended to have higher ferritin levels than the control group, which is consistent with the findings of another recent systematic review and meta-analysis that demonstrated a positive impact of Lp299v on iron absorption [138]. Further studies are warranted to investigate the potential impact of *L. plantarum* on iron status and absorption in high-risk groups such as pregnant women.

### *L. Plantarum* Consumption: Benefits or Limitations on Allergy

This study included only one clinical trial that evaluated the effects of *L. plantarum* on allergies, showing a positive clinical impact on the signs and symptoms of seasonal allergic illness. Further research is needed to elucidate the underlying mechanism of the direct impact of *L. plantarum* on the human systemic immune system. Nonetheless, the findings of this study suggest that long-term consumption of food containing *L. plantarum* may have potential benefits in alleviating allergy symptoms.

### *L. Plantarum* Consumption: Benefits or Limitations on Neonates

In contrast to IFN-γ and IL-17A, the combination of *L. plantarum* and Bifidobacterium strains with oligosaccharides and lactoferrin was found to reduce IL-5 levels in the study. The observed alterations in IL-10 levels may have a significant impact on the emergence of necrotizing enterocolitis (NEC). Further research should focus on elucidating the biological significance of probiotics' interactions with other antimicrobial substances and with each other.

### *L. Plantarum* Consumption: Benefits or Limitations on Pulmonary Disease

In a population of COPD patients with a history of frequent antibiotic use, consumption of a multispecies probiotic containing *L. plantarum* did not significantly influence the composition of the dominant fecal microbiota, in contrast to earlier findings in healthy volunteers. However, both the probiotic and placebo groups showed a significant but minor antibiotic impact, with increased yeasts and decreased *Bifidobacterium.* Comparison between the probiotic and placebo groups did not reveal any significant differences. Nonetheless, the probiotic group did show changes in certain bacterial groups over time.

### *L. Plantarum* Consumption: Benefits or Limitations on Nephrology

The initial findings from this study suggest that the administration of *L. plantarum* probiotic may have a favorable immunomodulatory effect in individuals with dyslipidemic nephrotic syndrome, as indicated by a tendency towards decreased serum levels of TNF-α and increased IL-10. Despite the lack of conclusive results, the significant clinical impact observed in favor of probiotic use in nephrotic disease has generated increased interest in releasing these preliminary findings as a basis for future clinical trials, building upon a previous study in which these results were published. Further research in this area is warranted to validate and expand upon these encouraging findings. [139].

### *L. Plantarum* Consumption: Benefits or Limitations on Mental Health and Behavioral Disorders

Acute and chronic stress are known to increase the risk of various medical illnesses, such as depression, post-traumatic stress disorder, cardiovascular conditions, asthma, peptic ulcers, ulcerative colitis, diabetes, and cancer [140, 141]. Probiotics, including *L. plantarum*, have been recognized for their role in the bidirectional communication between the gut and the brain, known as the gut-brain axis. This involves interactions between the central nervous system, neuroendocrine system, neuroimmune system, and gut microbiota [142]. The results of our study are promising, but further research is needed to fully understand the potential positive effects of probiotic interventions in individuals with mental health illnesses. Probiotics, including *L. plantarum*, may serve as a beneficial supplement for managing various physiological disorders.

### *L. Plantarum* Consumption: Benefits or Limitations on Obesity

Complications arising from obesity and overweight have a significant negative impact on patients' health status. The gut microbiota has emerged as a focus for therapeutic interventions in the treatment of obesity and overweight due to its direct connection with these medical conditions and their complications. Our study findings are in line with a previously published systematic review [143] which reported that combining *L. plantarum* and *L. rhamnosus* with a hypocaloric diet can promote weight loss. Our results suggest that *L. plantarum* itself may contribute to weight loss and reduction in fat mass in overweight individuals when combined with calorie restriction, leading to positive impacts on weight management. However, obesity is a multifactorial disease that can be influenced by various factors such as lifestyle, exercise, nutrition, and genetics. Therefore, it is unlikely that probiotics alone can be an effective method for reducing body mass index (BMI).

Meta-analysis did not show any significant results (*p* = 0.96). However, it should be noted that the studies included in our analysis were heterogeneous in terms of study design, population, and intervention protocols, which may have impacted the accuracy and reliability of our results.

### *L. Plantarum* Consumption: Benefits or Limitations on Oncology and Surgery

A recently published systematic review [144] suggested that probiotic supplementation may reduce the risk of postoperative infection complications in patients who underwent colorectal cancer surgery, although the results were promising. However, our study did not find any significant difference between the *L. plantarum* group and the control group in preventing postoperative infection complications. In contrast, another trial using a multi-strain probiotic formulation, which included *L. plantarum*, showed a reduced incidence of postoperative complications. This highlights the importance of reporting strain specificity of probiotics, as they are believed to be both strain-specific and disease-specific. Further research is needed to better understand the potential benefits of *L. plantarum* in preventing postoperative complications and to identify optimal strains, dosages, and treatment durations. [3].

### *L. Plantarum* Consumption: Benefits or Limitations on Infectious Disease

Our findings indicate that the supplementation of *L. plantarum* probiotics could potentially provide benefits to patients with infectious diseases, as evidenced by the reduction in IL-6 levels and improvement in chest X-rays, which may lead to a shorter stay in the ICU. This suggests that *L. plantarum* may have a positive impact on patients with certain infectious diseases. Based on the findings of our meta-analysis, we were unable to draw a definitive conclusion on the effectiveness of the intervention. The results were inconclusive, as there was no significant improvement observed in either group. It should be noted that one of the limitations of this meta-analysis is the high level of heterogeneity among the studies, as they differed significantly from one another. Future studies with more standardized methodologies and homogeneous populations are needed to obtain more reliable results.

### *L. Plantarum* Consumption: Benefits or Limitations on Hepatology

Our study did not yield statistically significant results with the intervention of *L. plantarum*. Further clinical trials specifically focusing on the *L. plantarum* strain are warranted to obtain conclusive findings.

## Limitation and Future Direction

We recognize several limitations in this systematic review, including (1) heterogeneity among included studies: substantial heterogeneity was observed among the studies included in the meta-analysis. The diverse study designs, populations, and intervention protocols may introduce variability that could impact the interpretation of the pooled results. (2) Geographical bias in study selection: most clinical trials were conducted in Asia (58 studies, 42.9%) and Europe (66 studies, 48.8%), which could limit the generalizability of our findings to a more global context. Future research should aim for more diverse participant populations worldwide. (3) Limited number of studies for certain conditions. Some health conditions under investigation may suffer from a limited number of included studies. This limitation affects the depth of our analysis for these specific conditions, emphasizing the need for additional research to validate the study conclusions. (4) Strain-specific focus and strain designation. Our decision to focus specifically on *L. plantarum* is a strength in providing a targeted analysis. However, the broader issue in the field of probiotic research—neglecting strain designation—remains a challenge. Strain-specific effects are crucial, and future reviews should emphasize reporting results with strain designations. (5) Potential publication bias. An inherent limitation in our review process is the potential for publication bias. Studies with positive outcomes are more likely to be published, possibly influencing the overall assessment of *L. plantarum’s* effectiveness in various health conditions. (6) Methodological heterogeneity across studies. Variability in methodologies, including differences in intervention protocols, outcome measures, and study durations, contributes to the observed heterogeneity. This diversity poses challenges in conducting a uniform analysis and drawing definitive conclusions. We acknowledge the need for standardized methodologies in future studies. Homogeneous populations and consistent protocols would enhance the accuracy and reliability of results, allowing for more meaningful comparisons across studies. (7) Furthermore, another critical limitation is the lack of mechanistic insights into how *L. plantarum* exerts its effects across various health conditions. Future research should prioritize mechanistic studies to provide a deeper understanding of the underlying actions of *L. plantarum*.

Recognizing these limitations, it is important to interpret our findings with caution. The identified constraints underscore the need for further research to address these shortcomings and contribute to a more comprehensive understanding of the role of *L. plantarum* in improving human health.

## Conclusion

In summary, this systematic review provided evidence that probiotic supplementation, either in combination with *L. plantarum* or *L. plantarum* alone, can significantly benefit patients with specific pre-existing medical conditions by improving test results or alleviating symptoms, in the field of periodontal, gastroenterological, mental, cardiovascular, endocrine, and dermatological health. The clinical significance of these findings underscores the importance of considering the specific probiotic strain and the type of disease in future research. Therefore, it is recommended that future studies adhere to reporting results with strain designations in line with best practices and recommendations in the field of probiotic research.

## Supplementary Information

Below is the link to the electronic supplementary material.

Supplementary file1 (XLSX 20 KB)

Supplementary file2 (DOCX 164 KB)

## Data Availability

No datasets were generated or analysed during the current study.
