# Supplementary material for: The Health Benefits of Probiotic Lactiplantibacillus plantarum: A Systematic Review and Meta-Analysis
Source: Probiotics Antimicrob Proteins. 2024 May 31;17(5):3358–77. doi: 10.1007/s12602-024-10287-3 (PMC12532744; doi:10.1007/s12602-024-10287-3)
Supplement: Supplementary file 2 — Supplementary file2 (DOCX 164 KB) [file 12602_2024_10287_MOESM2_ESM.docx]

**Table S1: Effect of *Lactiplantibacillus plantarum* in treating Oral diseases.**

| Author [Year] | Country | Disease/Oral health condition | Participants | Intervention | Intervention Duration | Follow ups | Outcome measurement | Study Findings | Quality Assessment |
| --- | --- | --- | --- | --- | --- | --- | --- | --- | --- |
| Kazuto Iwasakia  [2016] | Japan, | Chronic periodontitis | 36   - 17 [control] - 19 [intervention] - 66.2 years old (Mean) | - 10 mg *L. plantarum* HK L-137 capsule - Oral route - Once/day | 12 weeks | Week 4, 8 and 12 | - Pocket depth (PD) - Plaque index (PI) - Bleeding on probing (BOP) - Gingival index (GI) | - Pocket depth (PD) reduction (p < 0.05) in teeth with an initial PD ≥ 4 mm in the experimental group than in the control group at week 12 - BOP and the number of teeth or sites with PD ≥ 4 mm were significantly reduced in both groups by a successive supportive periodontal therapy - There were no differences in any of the clinical parameters between the control and intervention | Some concerns |
| P. Pudgar  [2021] | Slovenia | Periodontitis | 40   - 20 [control] - 20 [intervention]   25-80 years old | - *L. brevis,*   *L. plantarum*   - Topical gel and oral lozenges - One daily | 3 months | Month 3 | - Gingival bleeding index (GBI) - Plaque index (PII) - Pocket depth (PD) - Bleeding on Probing (BOP) - Gingival recession (REC) - CAL - Bacterial colonies CFU | - Both intervention and control groups showed substantial but equivalent improvements in periodontal parameters (DS, PII, GBI, PD, CAL, BOP) - The probiotic group had more reduced of BOP (p=0.048), but less healed disease sites (PD>4mm+BOP) (p<0.0051). | Low risk |
| Eduardo Montero [2017] | Spain | Gingivitis | 59   - 30 [placebo] - 29 [intervention] - 18-55 years old | - *L. plantarum, L. brevis, Pediococcus acidilactici* - Two tablets per day | 6 weeks | Week 6 | - Gingival index (GI) - Plaque index (PII) - Angulated bleeding score (AngBS) - Carriage of *Aggregatibacter actinomycetemcomitans, Porphyromonas gingivalis, Tannerella forsythia, Fusobacterium spp., Campylobacter rectus* | - - Both intervention and control groups significantly improved mean GI (p < .0001). However, there were no differences between the intervention and control for any clinical index.   - Treatment group with probiotics had a significant reduction of *T. forsythia* (p < .008). | Some concerns |
| E. Ferrés-Amat [2020] | Spain | Post-operative condition after surgical removal of third mola with osteotomy | 38   - 17 [control] - 21 [intervention] - 14-25 years old | - *L. brevis CECT7481; L. plantarum CECT7480* - Oral tablet - Twice a day for a week | 4 weeks | Week 1 and 4 | - Pain score via visual analogue scale (VAS) - Fever (y/n) - Alveolar osteitis (y/n) - Self-reported swelling, eating difficulties. - Plaque index (PI) - Gingival index (GI) | - No statistically significant difference in the infection rate between the control and intervention. - Intervention group had a significantly higher reduction in pain and eating difficulties scores at 5-, 6- and 7-days post-surgery (p=0.016, p=0.017 and p=0.031, respectively). - Swelling was not significantly different between groups at any time point. | High risk |
| Claudio Mongardini [2016] | Italy | Peri-implant mucositis | 40   - 20 [control] - 20 [intervention] - ≥18 years old | - 5ml of probiotics mixture (*L. plantarum; L. brevis)* delivered to peri-implant sulcus - Both intervention and control groups also consumed 1 probiotics tablet daily for 14 days. | 112 days | Week 2 and 6 | - Modified plaque index (mPII) - Bleeding on probing (BOP) | - No significant differences in clinical outcomes were observed between the intervention and control groups. | Some concern |
| Chiao Wen Lin  [2021] | Taiwan | healthy adults with 10⁵ S. mutans | 50   - Groups are not specified   10-40 years old | - *L. Salivarius subs. salicinius AP-32; L. paracasei ET-66; L. plantarum LPL28.* - Oral lozenges - 3 times per day | 4 weeks | Week 0, 2, and 4 | - IgA (human IgA ELISA kit) - Carriage of *S. mutans*, *p. gingivalis*, *f. nucleatum* (CFU) - Symptoms of oral health | - IgA was significantly increased in the intervention group - Probiotic reduced the occurrence of mouth rupture and pustules (p<0.01) - Significant reduction of *S. mutans* carriage in the mouth by 45% at week 2 and 75% at week 4 - Intervention group significantly reduced the total bacterial (p< .05) | Some concern |
| Dadgar S.  [2021] | Iran | Orthodontic treatment | 38   - 12 [fluoride] - 13 [probiotic] - 12 [placebo]   12-20 years old | - *L. plantarum* mixed with malt and dextrin - Mouthwash - Twice a day after lunch and before bedtime | 2 weeks | Week 2 | - Carriage of *S. mutans* (CFU) | - The CFU of *S. mutans* increased after 2 weeks in placebo (p=0.005) - The CFU of *S. mutans* reduced after 2 weeks in fluoride group (p=0.025) - The CFU of *S. mutans* did not change after 2 weeks in probiotic group (p=0.158). | Some concern |

**Note: Quantitative polymerase chain reaction (qPCR); Colony forming unit (CFU)**

**Table S2. Effect of *Lactiplantibacillus plantarum* in treating endocrinology diseases**

| Author [Year] | Country | Disease/health condition | Participants | Intervention | Intervention Duration | Follow-ups | Outcome measurement | Study Findings | Quality Assessment |
| --- | --- | --- | --- | --- | --- | --- | --- | --- | --- |
| Feizollahzadeh S  [2017] | Iran | Type 2 diabetes  (T2D) | 48   - 24 [control] - 24 [intervention]   35–68 years | - *L. gastro* A7 - 200 ml soy milk/day supplemented with *L. plantarum* A7 | 8 weeks | every 2 weeks | - Enzyme-linked immune   sorbent assay ELISA   - Standard enzymatic and Colorimetric method - International physical activity questionnaires (IPAQ) - 24 h diet   recall interview | - Intervention showed significant differences in Low-density cholesterol and high-density cholesterol changed significantly (p = 0.023, p= 0.017, respectively) - A significant difference was found between the two groups for dietary intakes of energy and carbohydrate. - Adiponectin, serum TNF-α, C reactive protein, and Fasting blood glucose did not show significant changes after probiotic treatment compared to placebo. | Some concern |
| Mitra Hariri  [2015] | Iran | Type 2 diabetes  T2D | 40   - 20[ control] - 20 [intervention]   32–68 years | - *L. plantarum* A7 - 200 ml soy milk/day supplemented with *L. plantarum* A7 | 8 weeks | every 2 weeks | - International physical activity questionnaires (IPAQ) - 24-h diet recall interview - Enzyme-linked immune   sorbent assay (ELISA)   - Real-time polymerase | - A statistically significant difference was found between the two groups for dietary intakes of energy and carbohydrate - Probiotic treatment determined a significant increase in superoxide dismutase (SOD) activity, and significant reductions in plasm concentration of 8-hydroxy-2'-deoxyguanosine (8-OHdG) compared to a placebo group. - There were no significant changes from baseline in the promoter methylation of MSH2 within either group | Some concern |
| Mitra Hariri  [2014] | Iran | Type 2 diabetes  T2D | 40   - 20 [control] - 20 [intervention]   25–65 years | - *L. plantarum* A7 - 200 ml soy milk/day supplemented with *L .plantarum* A7 | 8 weeks | every 2 weeks | - International physical activity questionnaires (IPAQ) | - A statistically significant difference was found between the two groups for dietary intake of energy and carbohydrate - Probiotic treatment determined a reduction in systolic and diastolic BP (Blood pressure) significantly. - There were not any significant differences in terms of BMI (body mass index) and waist-to-hip ratio | Some concern |
| D. R. Michael  [2020] | Bulgaria | obesity | 220   - 110 [control] - 110 [intervention]n   30–65 years | - (Lab4P) comprised L. *acidophilus, L. plantarum B. bifidum, B. animalis subsp. lactis* on a base of microcrystalline cellulose - One capsule daily | 9 months | 3 and 6 months | - Body weight, BMI (body mass index), waist circumference (WC), waist-to-height ratio (WtHR), blood pressure and plasma lipids - Plasma C-reactive protein (CRP), - Faecal microbiota - Quality of life (QoL) assessments - The incidence of upper respiratory tract infection (URTI). | - Probiotic treatment determined reduction in body weight (1.3 kg, p<0.0001), BMI (0.045 kg/m2, p< 0.0001), WC (0.94 cm, p<0.0001), and WtHR (0.006, p<0.0001) compared to the placebo group. - Reduction in small dense LDL-cholesterol (0.2 mmol/L, p=0.0241) in intervention group - Probiotic treatment showed improvements in QoL and the incidence rate ratio of URTI (0.60, p<0.0001) - Decreases in systolic blood pressure (SBP) were observed at 6 months in both groups with no significant between-group differences. | Low risk |
| Shujie Wang  [2021] | China | Newly diagnosed T2D | 183   - 91 [control] - 92 [intervention]   20–70 years | - The multi-strain probiotic products containing (*B. longum*, *B. breve*, *L. gasseri, L. rhamnosus, L salivarius, L. crispatus*,  *L. plantarum, L. fermentum, L. casei* - *4 g per 2 strips of powder, once daily at bedtime)* | 3-month | 3-month | - multiple ELISA - blood metabolomics measurement - Real-time quantitative RT-PCR, - liquid chromatography-mass spectrometry | - Neither the berberine group alone nor the probiotic group alone showed significant changes in postprandial cholesterols. - The berberine and probiotic treatment (Prob+BBR) had a greater reduction in PTC (postprandial total cholesterol) and low-density lipoprotein cholesterol (pLDLc) from baseline to week 13 than those in the placebo group. | Low risk |
| B. Abbasi  [2018] | Iran | T2D with nephropathy | 40   - 20 [control] - 20 [intervention]   25 and older | - *L. plantarum* A7 - 200 ml soy milk/day supplemented with *L. plantarum* A7 | 8 weeks | pre- and post-treatment  8weeks | - In sera (Biosystems analyzer A-15   Spain)   - TR-FIA method (Labmaster, Finland) | - Intervention resulted in significantly an increase in serum genistein (17.6±15.3 vs.4.5±2.3, p=0.002) and eGFR (15.9±10.8 vs. 3.2±8.4, p<0.001) - Intervention determined reduction of LDL-cholesterol (-9.2±10.4 vs. -2.2±5.2, p=0.01), total cholesterol (-12.4±4.8 vs. -4.87±14.7, p=0.04), non-HDL cholesterol (-15.3±4.5 vs. -5.9±14.7, p=0.01) and serum TG (-14.6±12.5 vs. -3.9±9.3, p=0.007) - No significant effect of intervention on serum HDL-cholesterol (1.11±3.38 vs. 0.90±2.7, p=0.8) and serum phosphorus (-0.14±0.10 vs. 0.05±0.5, p=0.1). | Some concern |
| Behnood Abbasi  [2017] | Iran | T2D with nephropathy | 40   - 20 [control] - 20 [intervention]   25 and older | - *L. plantarum* A7 - 200 ml soy milk/day supplemented with *L. plantarum* A7 | 8 weeks | every 2 weeks | - Time-resolved fluorescence immunoassay   method | - Intervention resulted in a significant reduction in albuminuria, serum creatinine (-0.17 ± 0.11 mg/dL versus -0.03 ± 0.08, P < .001), serum Interleukin-18   (-49.18 ± 48.22 mg/dL versus -9.03 ± 18.65 mg/dL, P = .002), and serum sialic acid (-17.4 ± 11.43 mg/dL versus -4.37 ± 9.91 mg/ dL, P = .001) compared with the control group.   - And a significant improvement in estimated GFR (15.9 ± 10.8 mL/min versus 3.2 ± 8.4 mL/ min, P < .001), and a marked increment in serum genistein (17.6 ± 15.3 mg/dL versus 4.5 ± 2.3 mg/ dL, P = .003) compared with the control group - No significant differences were detected in the other   dietary intake or physical activity, body  weight, body mass index, or waist-hip ratio during  the study | Some concern |
| Ratna Sudha  [2019] | India | Type 2 diabetes mellitu s  T2DM | 74   - 37 [control] - 37 [intervention]   Age18-65 years | - multi-strain probiotic UB0316-(*L. salivarius, L. casei, L. plantarum* UBLP40, *L. acidophilus, B. breve, and B. coagulans*, 30 billion CFU and fructo-oligosaccharide, 100 mg) - 2 capsules daily | 12 weeks | pre- and post-treatment (12 weeks) | - , HOMA-IR (homeostatic model assessment of insulin resistance) | - Intervention resulted in reduction in HbA1c (7.70 ± 0.79%; p = 0.0023) and weight (67.00 ± 8.82 kg; p < 0.001) as compared to placebo (HbA1c: 8.30 ± 1.35%; weight: 67.60 ± 9.46 kg) - No significant differences between the two groups in fasting blood glucose (FBG), HOMA-IR ,insulin, TC, TG, HDL, and LDL levels | Low risk |
| Mi-Ra Oh  [2021] | Korea | Isolated impaired glucose tolerance | 37   - 17 [control] - 20 [intervention]   Age19-70 years | - *L. plantarum* HAC01 - One capsule/day after meal each capsule contained 4x10⁹ CFU of LP HAC01 | 8 weeks | pre- and post-treatment and 3 additional visits | - Homeostasis model assessment [HOMA-IR - Quantitative insulin sensitivity check index (QUICKI) | - Intervention resulted in a significant reduction in 2h-PPG   (2 h postprandial glucose) and HbA1c levels compared to the placebo group ((p=0.045 & p=0.013 respectively)   - No significant change in fasting blood glucose & insulin, HOMA-IR, QUICKI, microbiota composition, and fecal SCFAs (fecal short-chain fatty acids) | Low risk |
| M. Miraghajani  [2019] | Iran | T2DM with nephropathy | 40   - 20 [control] - 20 [intervention]   Age32-68 years | - *L. plantarum* A7 - Oral route 200ml/day of soy milk with probiotic bacteria 2x10⁷cfu/ml of LP A7 | 8 weeks | baseline & after 8 weeks | - An inflammatory adipokine—Progranulin (PGRN), a cytokine receptor-soluble tumor necrosis factor receptor 1 (sTNFR1), and serum levels of Neutrophil gelatinase-associated lipocalin (NGAL) and cystatin C (Cys-C) | - Intervention resulted in a significant reduction in the Cys-C and PGRN levels compared with the placebo (P = 0.01) - A marginally significant in the NGAL level was seen between the two groups (P = 0.05) - There were no significant differences on the sTNFR1concenteration between two groups (P = 0.06) | Some concern |
| Sin Ji Lee [2013] | Korea | Obesity | 36   - 19 [control] - 17 [intervention]   Age19-65 years | - probiotic DUOLAC7 contains 5 billion viable cells of - strep. thermophiles, *L plantarum, L. acidophilus, L. rhamnosus, B. lactis, B. longum, B. breve* - Oral route, 1 capsule Twice daily | 8 weeks | At baseline and at week 8 | - Bioelectrical impedance analysis (BIA) - Inbody 3.0 (Biospace, Seoul, Korea) - Survey using the Korean version of the Obesity-related Quality of Life scale | - Both groups showed a significant reduction in weight & waist circumference (p =0.000) - No significant differences in body composition and metabolic markers were observed. | High risk |
| [Zohoor Nabhani](https://pubmed.ncbi.nlm.nih.gov/?term=Nabhani+Z&cauthor_id=29432772)  [2018] | Iran | Gestational diabetes mellitus  (GDM) | 90   - 45 [ control] - 45 [intervention]   Age18-40 years | - synbiotic capsule - consisting *of L. acidophilus, L. plantarum, L. fermentum, L. gasseri* (1.5-7.0 × 109-10 CFU/g) - with *fructooligosaccharide* (38.5 mg) - One capsule /day for 6 weeks | 6 weeks | At baseline and at week 6 | - Fasting plasma glucose (FPG) - Insulin, homeostasis model assessment-insulin resistance (HOMA-IR) - Quantitative insulin sensitivity check index (QUICKI) - High- low-density lipoprotein cholesterol (HDL-C, LDL-C) - Total cholesterol (TC), triglycerides (TG) - Total antioxidant capacity (TAC) - Systolic and diastolic blood pressure (SBP, DSP) | - Intervention resulted in a significant increase in HDL-C & TAC levels compared to placebo (p<0.05) - And a significant decrease in SBP & DBP compared to placebo (p<0.05) - There were no significant differences between the two groups regarding FPG, insulin resistance/sensitivity, lipid profiles, and TAC indices. | Low risk |
| Khaider K Sharafedtino  [2013] | Russia | Metabolic syndrome | 40   - 15 [control] - 25 [intervention]   Age 30-69 years | - *L. plantarum* TENSIA   1.5x10¹¹ CFU/g | 3  weeks | At baseline and the end of the study | - Blood pressure (BP) - Anthropometric characteristics - Markers of liver and kidney function, - Metabolic indices (plasma glucose, lipids, and cholesterol - Urine polyamines | - Intervention showed a significant reduction in Body mass index (BMI) (p=0.031) compared to the control group - Intervention showed a significant decrease in total water content (p=0.001) compared to the control group - In patients simultaneously treated with BP-lowering drugs, similar reductions in BP were observed in both groups - Intervention showed a positive association with a reduction in the morning diastolic blood pressure - No significant difference was in uric acid, total bilirubin, AST or ALT & blood glucose in probiotic vs controls - Significant difference was seen in urinary putrescine in probiotic versus control group - In the control group significant reduction in urinary polyamines was noted | Low risk |

**Table 3. Effect of *Lactiplantibacillus plantarum*in treating cardiovascular disease and Critical care patients**

| Author [Year] | Country | Disease/health condition | Participants | Intervention | Intervention Duration | Follow ups | Outcome measurement | Study Findings | Quality Assessment |
| --- | --- | --- | --- | --- | --- | --- | --- | --- | --- |
| Mari C. Fuentes  [2013] | Spain | hypercholesterolemia | 60   - 30 [control] - 30 [intervention]   Age 18 – 65 years | - mixture of three strains of *L. plantarum* (CECT 7527, CECT 7528 and CECT 7529) - one capsule orally daily. | 12 weeks | Week 6 and 12 | - Serum lipid profile (TC, LDL-C, OX-LDL, HDL-C)   Performed using Dimension RxL biochemistry analyzer.   - TAG, creatinine, aspartate transaminase, - alanine transaminase - g-glutamyl transpeptidase) - Anthropometric and safety parameters | - The intervention group showed a significantly lower TC values compared to the control group after 12 weeks. - After 12 weeks the values of TC, LDL-C, LDL-C:HDL-C ratio and OX-LDL in the Intervention group showed a significant reduction compared to baseline (13.6, 14.7, 19.7 and 13.6 %, respectively), and these reductions were higher than the ones observed in the placebo group (4.2, 5.8, 6.8 and 1.8 %, respectively) - no significant differences between the treatments in relation to weight, BMI, fat-free fat mass and fat mass after 12 weeks of consumption of the probiotic. - No significant differences were detected in lipid profile variables between the treatments. - No changes in anthropometric parameters and biochemical markers of safety were considered to be a result of treatment | Some concern |
| Guerrero-Bonmatty R  [2021] | Spain | hypercholesterolemia | 39   - 18 [control] - 21 [intervention]   Age 18 – 70 years | - *L. plantarumstrains* CECT7527 (KABP011™),   CECT7528 (KABP012™), and CECT7529 (KABP013™) + Monacolin K   - one capsule orally daily | 12 weeks | Week 6 and 12 | - Serum lipid profile (TC, LDL-C, OX-LDL, HDL-C, TG) - adverse effects - glycemia - hemoglobin | - Changes in LDL cholesterol and TC became significant compared to placebo (mean difference between groups and standard error of the mean = 23.6 *±* 1.5 mg/dL, p=0.023 and 31.4 ± 1.9 mg/dL, p=0.011, respectively) upon adjusting for the baseline imbalance in hypercholesterolemia treatment. - No adverse effects were noted during the study. - Intervention group showed significantly increase in HDL-C p=0.004 but not in the placebo. | Low risk |
| Fuentes, M. C. [2016] | Spain | hypercholesterolemia | 60   - 30 [control] - 30 [intervention]   Age 51.8 years | - Three strains of *L. plantarum: CECT 7527, CECT 7528, and CECT 7529.* - One capsule orally | 16 weeks | Week 6, 12 and 16 | - Serum lipid profile (LDL-C, HDL-C   OX LDL, TC, TG) | - At 12 weeks, compared to placebo, the intervention group had significantly (P<0.001) larger reduction in LDL-C (24.4 vs 9.8 mg/dL), total-C (33.7 vs 10.6 mg/dL), LDL-C//HDL-C ratio (0.8 vs 0.3), oxidized LDL (7.5 vs 1.0 U/L) and TG (29.1 vs 4.1 mg/dL) - HDL-C was also significantly (p<0.001) increased in intervention group vs placebo (2.9 vs 0.4 mg/dL) | Some concern |
| Arrigo F. G. Cicero  [2021] | Italy | MetS syndrome | 60   - 30 [control] - 30 [intervention]   Age 65-80 years | - *L. plantarum* PBS067, *L. acidophilus* PBS066 and *L. reuteri* PBS072 - Orally/ liquid vial daily | 60 days | Day 60 | - Serum lipid profile (TC, TG, HDL-C   LDL-C)   - FPG - (FPI) - mini nutritional assessment (MNA) examination - a short version of the global physical activity questionnaire (GPAQ) - EuroQol-5 Dimension (EQ-5D) questionnaire - ELISA, Endocheck | - The intervention group experienced a statistically significant improvement in waist circumference and in fasting plasma insulin, total cholesterol, high-density lipoprotein cholesterol, non-HDL-C, triglycerides (TG), LDL-C, HDL-C protein and tumor necrosis factor alpha serum levels, compared both to the baseline and the control group. - Visceral adiposity index improvement in the intervention group was significantly greater than in placebo group. - No significant change was observed in the other considered parameters. | Some concern |
| Edward Litton [2021] | Australia | Critical illness | 218   - 108 [control] - 110 [intervention] | - *L. plantarum 299v* - Oral capsule | 60 days | Day 60 | - Quality of life was assessed using the five-level EQ-5D (EQ-5D-5L) questionnaire - Visual analogue scale (EQ VAS) - Days alive and out of hospital to Day 60 (DAOH_60_). | - A post hoc exploratory analysis suggested significant increase in DAOH60 and antibiotic-free days in the subgroup of 24 participants with septic shock at baseline - There were no significant between-group differences observed in the components of the primary outcome (DAOH_60_), or when analysis was limited to participants with ≥ 80% compliance. - overall quality of life at Day 60, as assessed by median EQ-5D-5L VAS scores was similar in the probiotic and placebo groups | Low risk |

high-density lipoprotein cholesterol (HDL-C). triglycerides (TG). Total Cholesterol (TC). Low -density lipoprotein-cholesterol (LDL-C). oxidized LDL-cholesterol (OX-LDL). plasma triglyceride (TG). Fasting plasma glucose (FPG). fasting plasma insulin (FPI)

**Table S4. Effect of *Lactiplantibacillus plantarum* in treating Dermatology**

| Author [Year] | Country | Disease/health condition | Participants | Intervention | Intervention Duration | Follow-ups | Outcome measurement | Study Findings | Quality Assessment |
| --- | --- | --- | --- | --- | --- | --- | --- | --- | --- |
| Zhifeng Fang  [2020] | China | Atopic dermatitis (AD) | 69   - 26 [control] - 43 [intervention]   age: between 49.19 and 53.57 | - *L. plantarum* CCFM8610 - 109 CFU daily of lyophilized powder | 8 weeks | after 8 weeks, Serum samples were also collected  at two time points | - SCORAD index, - DLQI (Dermatology Life Quality  Index) questionnaire - Serological indicators, such as the levels of IgE, interleukin 4 (IL-4), IL-13, and IL-10 | - CCFM8610 group had a significantly improved SCORAD index compared with their baseline values and compared to other groups. - Improvement in (DLQI) but no significant difference between groups - CCFM8610 group showed a significant increase in the expression levels of IL-10 - CCFM8610 intervention showed a significant functional gene difference in the gut microbiota. | Some concern |
| Mi-Ju Kim [2021] | Korea | Acne  vulgaris | 28   - 14 [control] - 14 [intervention]   Age 19–39 years | - *L. plantarum* CJLP55 - 1.0 ×1010 CFU daily for 12 weeks | 12 weeks | at 0  week (baseline) and 12 weeks | - Criteria scale of Investigator’s   Global Assessment (IGA)   - Photographs - Sebumeter (SM815) - Corneometer   (CM825)   - skin pH-meter (PH905) - High-performance thin-layer chromatography (HPTLC) - Polymerase chain reaction (PCR) | - Intervention showed a significant decrease in ILC, TLC, and acne grade for all subjects (male and female combined) at 12 weeks. - The percent change of ILC for male and female subjects between intervention and placebo groups was not significantly different, but it was significantly decreased by 42.09% (95% confidence interval (CI), −80.57 to −3.61; p=0.033) for all subjects in the intervention group compared with the placebo group - Intervention group showed a significant decrease in TLC by 41.38% (95% CI, −73.67 to −9.10; p=0.017) for males, 45.51% (95% CI, −94.67 to 3.65; p=0.031) for female, and 49.67% (95% CI, −82.76 to −16.59; p=0.002) for all subjects - Intervention group showed a significant decrease in acne grade by 29.33% (95% CI, −51.36 to −7.29 to; p=0.030) for males, 29.89% (95% CI, −54.04 to −5.74; p=0.012) for female, and 28.99% (95% CI, −44.23 to −13.75; p=0.009) for all subjects, compared with the placebo group. - No change in skin pH and hydration in both groups | Low risk |
| Angela Michelotti  [2021] | Italy | Atopic dermatitis | 80   - 40 [control] - 40 [intervention]   Age13-50 years | - a mixture of lactobacilli *(L. plantarum PBS067, L. reuteri PBS072 and L. rhamnosus LRH020*) - one capsule/day | 56 days | at T(0d), T(28d) and T(56d), and then at T(84d) (after a one-month wash-out) | - SCORAD index)   SCORing Atopic Dermatitis   - Skin moisturization was measured according to the Corneometer® method, using Corneometer® CM 825 (Courage + Khazaka, electronic GmbH). - Trans epidermal water loss (TEWL) was measured indirectly using a Tewameter® TM 300 (Courage + Khazaka, electronic GmbH). - Skin reactions, both physical (erythema, edema, dryness, and desquamation) and functional (tightness, itching, and burning), were scored according to a clinical 5-point scale (0 = none; 1 = very mild; 2 = mild; 3 = moderate; 4 = severe) | - Intervention showed a statistically significant and progressive decrease in SCORAD index throughout the administration period (from 20.9±0.5 at T0d to 16.9±0.5 at T28d and to 13.7±0.6 at T56d), moreover, improvement in SCORAD index remained favorable after one month of discontinuation of the product (14.8±0.6 at T84d) - No differences between the two groups were recorded for erythema and edema | High risk |
| Hyeon-Jong Yang  [2014] | Korea | Atopic dermatitis | 100   - 50 [control] - 50 [intervention]   Age 2-9 years | - (*L. casei, L. rhamnosus, L. plantarum, and B. lactis*)   1×10^5^ of each  bacterial strain (Powder) | 6 weeks | Weeks 0 and 6 | - Eczema area severity index (EASI) - Visual analogue scale for pruritus (VASP) | - Both groups showed a significant improvement in the clinical severity - the cytokine levels between the 2 groups were not significantly different in week 6 (IL-4, p=0.50; IL-10, p=0.58; TNF-α, p=0.82) | High risk |
| C.R.S. Prakoeswa  [2017] | Indonesia | Atopic dermatitis | 22   - 10 [control] - 12 [intervention]   Age 0-14years | - *L. plantarum* IS-10506 - 2 capsules daily | 12 weeks | Weeks2,8, 12 | - Scoring Atopic Dermatitis Index (SCORAD) - Serum immunoglobulin E (IgE), interleukin (IL)-4, interferon gamma (IFN-γ), forkhead box P3 (Foxp3+)/IL-10, and IL-17 levels | - Intervention showed a significant decrease in SCORAD index and levels of IL-4, IFN-γ, and IL-17 - Intervention showed significant increase in FOXp3+/IL-10 ratio compared to placebo - No significant changes in IgE in both groups | Some concern |
| Dong Eun Lee  [2015] | Korea | Dry skin | 110   - 49 [control] - 61 [intervention]   Age 41-59 years | - *L. plantarum* HY7714 - 2 g daily of a powder containing   HY7714 | 12 weeks | Baseline and at 4, 8, and 12 weeks | - acorneometer (CM825 - Courage and Khazaka Electronic GmbH - Transepidermal water loss from the face, forearm, and hand was measured using a vapometer - 3D skin imaging system - Glossmeter - Cutometer | - Intervention showed significant increases in the skin water content in the face (p<0.01) and hands (p<0.05) at week 12 - Transepidermal water loss decreased significantly in both groups at weeks 4, 8, and 12 (p<0.001 compared with baseline) - Intervention showed significant improvement in skin gross by week 12 - Intervention showed a significant reduction in wrinkle depth at week 12 - Skin elasticity in the intervention group improved by 13.17% (p<0.05 vs. controls) after 4 weeks and by 21.73% (p<0.01 vs. controls) after 12 weeks | Some concern |

**Table 5. Effect of *Lactiplantibacillus plantarum* in treating Gastroenterology**

| Author [Year] | Country | Disease/Oral health condition | Participants | Intervention | Intervention Duration | Follow ups | Outcome measurement | Study Findings | Quality Assessment |
| --- | --- | --- | --- | --- | --- | --- | --- | --- | --- |
| Philippe Ducrotte  [2012] | India | Irritable bowel syndrome  [IBS] | 214   - 108 [intervention] - 106 [placebo] - 18-70yrs old | - *L. plantarum* 299v (DSM 9843) 10 billion CFU/ capsule - Oral - Once daily | 4 weeks | Weeks  2,3 and 4 | - VAS scale (1-10) Abdominal pain episode - Severity & frequency of abdominal bloating | - Pain severity (0.68 + 0.53 vs 0.92 + 0.57, P < 0.05) & frequency (1.01 + 0.77 vs 1.71 + 0.93, P < 0.05) lower in intervention grp compared to placebo - Greater reduction in stool frequency, bloating in intervention grp compared to placebo - Treatment efficacy (good/excellent) was higher in intervention grp than placebo reported both by patients and investigators | Some concern |
| Ana D Cano-Contreras  [2022] | Mexico | Lactose intolerance (self-reported) | 48 (2:1 randomization)   - 33[intervention] - 15[placebo] - 18-64yrs old | - i3.1 (3 probiotic strains)   *P. acidilactici* strain CECT7483 (KABP-021) and *L. plantarum* strains CECT7484 (KABP-022) and CECT7485 (KABP-023  [3×10^9^ colony-forming unit CFU/dose]   - Oral - Once daily | 8 weeks | Baseline & week 8  (phone calls – week2,4,6) | - Change in total symptom score using validated questionnaire for lactose malabsorption screening - Change in symptom subscore & in area under AUC curve of lactose hydrogen breath test post lactose challenge (25g) - Safety and tolerability profile of the probiotic | - Total symptom score reduced significantly from baseline to wk 8 after lactose challenge in intervention grp compared to placebo (-5.11 vs. -1.00; P<0.001) - Except vomiting scores all symptom subscores decreased from baseline to wk 8 in the intervention grp compared to placebo - Higher reduction in abdominal pain (p=0.045) & flatulence (p=0.004) was seen in probiotic grp - The AUC of LHBT significantly reduced in probiotic grp compared to placebo (p=0,019). No correlation between change in AUC and total symptom scores or subscores. - Between grp AUC differences were non-significant (p>0.05) | Some concern |
| Farup PG  [2012] | Norway | Irritable bowel syndrome  [IBS] | 16   - Crossover trial same patients received intervention & placebo - 18-75 yrs old | - *L. plantarum* MF 1298 10(10) CFU/capsule - Oral - Once daily | 1-wk run in period, followed by 2 (3-wk) treatment phase separated by a 4-wk washout | End of run-in period, wash-out period and 2 treatment periods | - IBS symptom score (scale 0-15) - Composition of fecal microbiota reported as relative proportion of B*acteroides*, F*aecalibacterium*, *Lachnospiraceae* - Simpson’s D and Shannon’s H diversity was calculated for 3 patients as alternative | - Significant ‘treatment effect’ on IBS symptom score was noted. Active t/t induced increase in IBS symptom score of 1.57 compared to placebo (p=0.004) - Correlation b/w changes in IBS symptom scores & changes in fecal microbiota and Simpson’s & Shannon H diversity indices was nonsignificant - Treatment was not predictive of changes in fecal microbiota, or Simpson’s D diversity index - LP MF1298 had unfavorable effects on symptoms most likely direct effect of bacterium on gut wall and not an indirect through change in gut microbiota | Some concern |
| Francavilla R  [2019] | Italy | Celiac disease with IBS type symptoms | 109   - 54[intervention] - 55[placebo] - >18yrs of age | - 5 strains of lactic acid bacteria & *bifidobacterium*   *Lactobacillus casei* LMG 101/37 P-17504, *L. plantarum* CECT 4528 *B. animalis subsp. lactis Bi1* LMG P-17502 *B. breve* Bbr8 LMG P-17501, *B. breve* Bl10 LMG P-17500   - Oral - 1 sachet/ day | 14 weeks  [6 wk t/t period preceded by 2 wk run-in period followed by 6 wk follow-up] | Questionnaire at 2-wk interval,Fecal samples at wk 2, 8 & 14 | - Improvement in GI symptoms [IBS-SSS] - T/t success [decrease in 50% of symptom scores] - improve GSRS - changes in stool parameters [BSFS] - improve IBS-QoL - modification of gut microbiota and metabolomic fecal profile | - Baseline IBS-SSS, GSRS & BSFS scores differed significantly between probiotic & placebo grps. At the end of treatment significant decrease was noted (p<0.05) - When expressed a variation from pre-treatment values IBS-SSS, GSRS & BSFS scores in probiotics was significantly reduced compared to placebo (p<0.05). At the end of follow-up non-significant change was noted - After t/t, IBS-QoL scores did not differ in pts receiving placebo or probiotic either as absolute values or as variation from pre-t/t values (p=NS) - t/t success was higher in pts receiving probiotic compared to placebo (p<0.04) - At 6-wk total anaerobes increased compared to baseline in pts receiving probiotic (p=0.018) - Presumptive lactic acid bacteria, Staphylococcus and Bifidobacterium, increased in patients receiving probiotic treatment | Low risk |
| Diego A. Barraza-Ortiz  [2021] | Mexico | IBS-D predominant or mixed type | 55 (3 groups)   - 18 [i3.1 probiotic only] - 19 [i3.1 probiotic plus alverine and simethicone] - 18 [placebo] - 18-59 yrs | - 3 lactic acid bacteria: - *L. plantarum CECT 7484, L. plantarum CECT 7485, and P. acidilactici* CECT 7483 (3 × 10^9^ CFUs) - Oral - Once daily - grp II – oral capsule thrice daily | 6 weeks | Baseline & wk 6  [weekly phone calls] | - Response rate in QoL – (IBS-QoL score 0-100) - Abdominal pain (VAS, scale 0-10) - stool consistency BSS scale 1-7) - Changes from baseline | - All 3 grps, presented significant improvement in IBS-QoL scores (p<0.0001) with largest improvement in probiotic +antispasmodic - The IBS-QoL rate of response was 50% for patients in the group with probiotic alone, 68.4% in the group with probiotic plus antispasmodic, and 16.7% in the group with placebo after 6 weeks of treatment (p=0.005) - Response to abdominal pain was reported by 38.9% of patients treated with probiotic, 57.9% with probiotic plus antispasmodic, and 16.7% with placebo, p=0.035 - For stool consistency, a response to treatment was reported by 44.4% of patients treated with probiotic, 57.9% with probiotic plus antispasmodic, and 16.7% with placebo p=0.032. - Significant differences were seen only in probiotics & probiotics + antispasmodic | Some concern |
| Lorenzo Drago  [2021] | Italy | Functional  dyspepsia | 2676   - 1357 [PDS] and 1319 [EPS] - 767 [probiotics alone] - 1115 [probiotics + PPI] - 479 [probiotics + prokinetics] - 315 [probiotics +antacids] | Abivisor probiotic formulation   - *L. acticaseibacillus rhamnosus LR04 (DSM 16605), L. pentosus LPS01 (DSM 21980), L. plantarum LP01 (LMG P-21021), and L. delbrueckii subsp. delbruekiiLDD01 (DMS 22106)* - Oral - ≥5 ×10^9^ CFU/AFU per day, alone or with standard therapy | 30 days | At the start  of the study (T0) and at 15 days following  the end of probiotic treatment (T1) | - Evaluation of the presence of two specific clinical symptoms associated with PDS (postprandial filling and early satiety) and with EPS (epigastric pain and epigastric burning) at T0 &T1 - Evaluation of the best therapy between probiotic alone or in combination with other pharmaceutical approaches | - Both postprandial filling and early satiety showed a statistically significant improvement between the beginning and end of treatment in both probiotics alone or combined with PPIs, prokinetics or antacids in PDS group. - Epigastric pain and Epigastric burning significantly decreased after treatment with Probiotics alone or combined with PPIs, prokinetics or antacid in EPS group. - All four pharmacological combinations prescribed to patients with EPS improved the perception of all minor symptoms at T1 - Probiotics alone treatment has beneficial effects in reducing PDS symptoms, while patients with EPS showed no between t/t differences | High risk |
| Åsa Håkansson  [2019] | Sweden | Children with active naïve celiac disease autoimmunity | 78   - 40 [probiotics] - 38 [placebo] - 3-7 yrs old | - *L. plantarum HEAL9 and L. paracasei 8700:2* - Oral - Once daily | 6 months | Baseline, at 3-months, at 6-months | - Changes in autoantibodies and regulatory T-cells assessed as change in peripheral immune response as B   cells, NK and NKT cells, subpopulations of regulatory T cells and changes in serum levels of tTG autoantibodies | - Comparing probiotic vs placebo, significant differences in changes in cell populations expressing CD3-CD56+ & CD3+CD56 was observed (p=0.008) - Significant differences were observed in naïve Th cells, memory and effector Th cells after six months of probiotics - Consistent changes in the peripheral immune response involved in regulating T-cells population were seen in placebo grp only - Peripheral changes in NKT cells over time was observed in placebo grp only - No difference in median levels of tTG autoantibodies between both grps over time - Although it cannot be proven that probiotics can prevent celiac disease, daily oral administration of L. Plantarum HEAL9 & L.Paracasei 8700:2 may modulate peripheral immune response in children with CDA | Low risk |
| Ahmet Baştürk  [2017] | Turkey | Children with functional constipation according to ROME III diagnostic criteria | 146   - 72 [intervention] - 74 [placebo] - 4-18 yrs old | Synbiotic –   - *L. casei, L. rhamnosus, L. plantarum, B.* - *lacti*s and prebiotics at a dose of 1996.57 mg 4x10^9^ CFUs - oral - 1 sachet daily | 4-weeks | Baseline & at the end of one month | - Complete benefit by resolution   of all complaints of the patient with the 4-wk synbiotic  t/t [BSS scale]   - frequency & complaints [weekly number of defecations, stool consistency, weekly number of fecal incontinence,abdominal pain, painful defecation, rectal bleeding] - incidence of side effects [vomiting and diarrhea] | - After 4-wks of probiotics significant improvement in symptoms such as abdominal pain, weekly number of defections, pediatric Bristol stool scale was observed in Synbiotic grp versus placebo (p<0.05) - Between grp significant difference in complete treatment benefit (p<0.001) - Complete treatment benefits were achieved in 66.7% in probiotic grp & 28.3% in placebo - Use of Synbiotic has beneficial effects in children with functional constipation | Some concer |
| Bong Ki Cha  [2012] | Korea | Diarrhea dominant – IBS | 50   - 25 [intervention] - 25 [placebo] - 18-65yrs old | Duolac7   - *L. acidophillus (KCTC 11906BP), L. plantarum (KCTC11867BP), L. rhamnosus (KCTC 11868BP), B. breve (KCTC 11858BP), B. lactis (KCTC 11903BP), B. longum (KCTC 11860BP), and Strep. thermophilus (KCTC 11870BP)* - Oral - One capsule twice daily | 8 weeks t/t duration and  2-wk  Follow-up | Weekly assessment of symptoms for 8-weeks  Fecal samples were collected at baseline & end of t/t  [phone calls to assessment of AR] | - Relief of overall IBS symptoms [10-point VAS scale for each symptom] - Proportion of subjects reporting AR of their IBS symptoms at least 50% of the weeks during t/t & post-t/t periods - Effect of intervention on D-IBS related symptoms, stool frequency (BSS scale), consistency & IBS- QoL | - Consistently higher proportion of AR was seen in probiotic grp compared to placebo throughout the 10-wk period (p<0.05) - Proportion of responders was significantly higher on probiotic grp vs placebo (48% vs 12%, p=0.01) - Significant improvements in stool consistency, IBS-QoL was seen in probiotic grp compared to placebo - Comparison of denaturing gradient gel electrophoresis profiles of fecal microbial composition showed that concordance between bacterial composition before and after t/t was significantly higher in probiotics grp compared to placebo [69.5% vs. 56.5%, p=0.005]. - Duolac7 can be used in combination with therapeutic agents for treating D-IBS. | Some concern |

| Stefano Guandalini  [2010] | Italy and India | Children affected by IBS | 118   - 59 [intervention] - 59 [placebo] - 4-18 yrs old | VSL#3   - *B. breve, B longum, B infantis, L. acidophilus,* - *L plantarum, L casei, L bulgaris, and Streptococcus* - Thermophilus - Oral - One sachet/day for 4-11yrs old & 1 sachet twice daily for 12-18 yrs old | 16 weeks  [Randomized to t/t or placebo for 6 weeks, wahout period for 2 weeks, crossover t/t for 6-wks] | Follow-up visits on days 1,15,29, 43, 57, 71, 85, 99, 113 | - Improvement in subject’s global assessment of relief (questionnaire -SGARC modified for children) - improvement in symptoms - abdominal pain/discomfort, stool pattern, bloating/gassiness, & family assessment of impact of their childs IBS on family life | - Relief of symptoms (SGARC) changes from baseline were statistically significant at wk 2 (p<0.05) and remained such at wk 4 (p<0.01) & wk 6 (p<0.001), whereas in placebo they were significant at wk 4 (p<0.05) & wk 6 (p<0.05) - Magnitude of change on scores before and after t/t was statistically significant in probiotics grp (end of treatment arm – start of treatment arm) [p<0.05] - VSL#3 was superior the subjective assessment of relief of symptoms as well as 3 or 4 secondary endpoints such as abdominal pain/discomfort (p<0.05), bloating/gassiness (p<0.05), family assessment of child’s life disruption (p<0.01). - A non-significant trend was seen in stool pattern. | Some concern |
| --- | --- | --- | --- | --- | --- | --- | --- | --- | --- |
| Solveig C Ligaarden  [2010] | Norway | IBS | 19   - 10 [1^st^ intervention period] - 9 [2^nd^ intervention period] - 18-75yrs old | - *L.plantarum* MF1298 (freeze-dried)   1×10^10^ CFU   - Oral - Once daily | 1-wk run-in period followed by two 3-wk intervention period separated by 4-wk washout period | t/t preferences at the last visit, satisfactory relief of symptoms (Y/N) at the end of run-in period & wash-out period, weekly during t/t period | - Treatment preference - number of weeks with satisfactory relief of symptoms and IBS sum score (BSS scale for stool freq & consistency) | - 81% (13 participants) preferred placebo to L.plantarum MF1298 (95% CI 57%, 93%, p=0.012) - Number of weeks with satisfactory symptom relief was significantly higher in placebo compared to intervention [1.44 vs 0.5, p=0.006] - IBS sum score for diarrhea was significantly higher for intervention compared to placebo - L.plantarum was not detected in fecal samples at run-in, wash-out periods or in placebo & was detected in all fecal samples at the end of active t/t period. - Compliance was 95% - L. Plantarum MF1298 may be an unfavorable strain & this should stimulate basic research on the molecular basis of probiotic properties. | Some concern |
| K Niedzielin  [2001] | Poland | IBS | 40   - 20[intervention] - 20 [placebo] - 27-63 yrs old | - *L. plantarum* 299V   5×10^7^ CFU/mL   - Oral - 200ml twice daily | 4 weeks | At baseline and end of study  [Weekly assessment of symptoms] | - abdominal pain relief - change in overall scores applied by assessing IBS symptoms | - All patients treated with LP299v had resolution of abdominal pain symptoms when compared to placebo (p=0.0012) - Trend towards normalization of stool frequency in constipated patients treated with LP299v compared to placebo (6/10 vs 2/11 patients respectively) p=0.17 - All IBS symptoms (pain, constipation, diarrhea, flatulence) complete resolution in 45% pts, partial resolution in 50% & no resolution in 5% in probiotics grp. Whereas in placebo grp 15% reported complete resolution & 85% no improvement (p<0.0001) - No treatment related side effects | Some concern |
| Heidi Maria Staudache  [2017] | Unitedkingdom | IBS – diarrhea predominant, mixed subtype and unsubtyped IBS | 104 [4 grps, 2-by-2 factorial]   - 27 [sham diet/placebo] - 26 [sham diet/probiotic] - 24 [low FODMAPdiet/placebo] - 27 [low FODMAP diet/probiotics] - 18-65yrs old | Multi-strain preparation   - S. *thermophilus DSM 24731, B. breve DSM 24732, B. longum DSM 24736, B. infantis DSM 24737, L. acidophilus DSM 24735, L. plantarum DSM 24730, L. paracasei DSM 24733, L. delbrueckii subsp. bulgaricus DSM 24734* - Oral - 2 sachets (11.95 log10 bacteria)/day | 4 weeks | At baseline and after 4-weeks,  (Incidence of 15 GI symptoms & overall symptoms measured daily)  (weekly telephone calls on occurrence of AEs) | - Impact of diet & supplements on symptoms & microbiota - Adequate relief of symptoms & stool *bifidobacterium* species abundance at 4-weeks - Individual GI symptoms (IBS-SSS & GSRS), stool output, HRQoL, microbiota diversity and nutrient intake | - Significantly higher proportions of patients on the low FODMAP diet had adequate symptom relief than in the sham diet (61% vs 39%, p=.042) with odds of symptom relief 2.43 (1.03<->5.75) in per protocol analysis - Mean total IBS severity symptom score was significantly lower for pts on low FODMAP diet than sham diet - Significant differences were observed in absolute abundance of Bifidobacterium species measured using qPCR between the four randomized groups. (mean difference -0.67, p=0.02) - No significant differences in global microbiota alpha diversity and beta diversity (Bray Curtis dissimilarity index) for low FODMAP diet compared to sham diet or probiotics compared to placebo | Some concern |
| Nikos Viazis  [2022] | Greece | *H. pylori* positive chronic gastritis or peptic ulcer disease | 741   - 371 [eradication regimen +probiotics] - 370 [eradication regimen +placebo] - >18 yrs old | *Lactolevure* (4 probiotic strains)   - *L. acidophilus, L. plantarum, B. lactis, and S. boulardii* - Oral - Twice daily | 15 days  (in addition to 10-day eradication therapy) | H. pylori eradication tested 6-wks after completion of eradication therapy | - Rate of reduced adverse events with probiotics and recommended eradication regimen - Test if adding probiotics increased the eradication rate | - H. pylori was successfully eradicated in 92% (303 subjects) in probiotics grp compared to 86.8% (291 subjects) in placebo (p=0.028) - The odds of eradication were higher in probiotic compared to placebo (OR=1.76, 95% CI 1.06-2.94) - Treatment satisfaction reported as mean, SD and assessed by TSQM questionnaire was 85.6 (11.9) in Group A and 75.2 (12.6) in group B (p<0.001). - Grade 3 severity of symptoms was reported in 1.2% in probiotic grp vs 14.6% in placebo, whereas grade 1& 2 severity of symptoms was reported in 15.8% in probiotic grp vs 38.2% in placebo [p<0.00001] - The ratio of adverse events to no adverse events was 14 times less in probiotic grp compared to placebo. | Some concern |
| Mañé, J  [2011] | Spain | GI disorders such as acute infections & antibiotics associated with diarrhea in adults, ulcerative colitis, pouchitis, & IBS | 47   - 13[low probiotic dose] - 19 [ high probiotic dose] - 15 [placebo] - Institutionalized elderly >65yrs old | 2 new probiotic strains   - *L. plantarum-*-CECT 7315 and 7316 - Oral - Single daily dose | 24 weeks | Baseline, end of t/t (wk 12), follow up (wk 24) | - Effect of probiotic mixtures on blood immunological parameters - Cytokine levels of IL-1-10, TGF-β1 - T-cell numbers, changes of NK and dendritic cell proportions, B-cells in the blood | - Significant decrease in plasma TGF-β1 after treatment with both probiotic doses at the end of follow-up period while no change was observed in placebo. - High probiotic dose resulted in increase in percentages of activated T-suppressor (CD8+CD25+) and NK (CD56+ CD16+) cells while low probiotic doses increased activated T-helper lymphocytes (CD4+CD25+), B lymphocytes (CD19+), antigen presenting cells (HLA-DR+) - Incidence of infections during treatment showed a significantly lower trend in high probiotic dose. - Significant trend of mortality was greater in placebo grp compared to both probiotic doses grps | Some concern |
| Haiyan Xu  [2021] | China | IBS | 45   - 24[intervention] - 21 [placebo] - Mean age - 37±15.1 yrs old | 3 different probiotic strains   - *L. casei, L. plantarum P-8, and B. animalis subsp. lactis V9* - Oral - Single daily dose | 28 days | Serum samples at baseline (day 0) and end of t/t (day 28),  Fecal samples – days (0, 7, 28) | - Clinical improvement of IBS (IBS-SSS) symptoms - Changes in fecal microbiota structure and composition - Changes in serum cytokine levels – IL 6,8, TNF-α | - The IBS-symptom severity score (p<0.01), serum levels of IL-6 (p<0.01) & TNF-α (p<0.001) were significantly lower in probiotic grp than control at day 28. - Adjunctive probiotics resulted in significant reduction in pathogenic bacterial genera that worsen IBS symptoms such as Bacteroides (p<0.01), Escherichia (p<0.05), Citrobacter (p<0.05). Significant reduction in beneficial bacteria such as Bifidobacterium (p<0.05), Eubacterium (p<0.05), Dorea (p<0.01), Butyricicoccus (p<0.05) - Clinical improvement of IBS symptoms was associated with gut microbial modulation (changes in fecal microbial composition) - Initial fecal microbial composition influenced clinical outcomes | Some concern |
| Sören Nobaek  [2000] | Sweden | IBS | 52   - 25 [intervention] - 27 [placebo] - Age <18 yrs old | - 5 x 10^7^ cfu/ml of *L. plantarum* (DSM 9843) - Oral - Single dose (400ml) daily | 4 weeks | GI functions recorder 2 wks before administration (wk 1+2) and during treatment (wk 3-6)  [questionnaire 12 months after study period]  Fecal samples before & after study period | - GI parameters -number of defecations, fecal consistency, presence/absence of abundant gas - VAS scale for overall GI function, defecation function, abdominal pain and flatulence - Changes in fecal microbial composition - CRP, TSH & Hb levels | - Significant and rapid reduction in flatulence was observed in the experimental grp compared to placebo (number of days with abundant gas production, test group 6.5 (before), 3.1 (after) vs 7.4 (before) and 5.6 (after) for the placebo group). But at the end of the study period significant decrease was also seen in the control grp - Significant improvement in the function of defecation assessed by VAS in the experimental grp compared to control group. - Abdominal pain reduced whereas no significant difference was observed in bloating both groups - Subjects in the probiotic’s grp maintained an overall GI function when compared to placebo grp - No significant changes in Enterobacteriaceae in either grp before or after study period [enterococcus increased in placebo grp and remained unchanged in test grp]. | Some concern |
| Beril Turan  [2021] | Turkey | IBS (newly or previously diagnosed according to ROME IV criteria & whose IBS therapy was not modified for the past 4 weeks) | 85   - 43 [low FODMAP + probiotic] - 42 [low FODMAP + placebo]   Age 18-65 yrs old | - Probiotic product *S. thermophilus* (5.4x10^8^ cfu), *B. lactis* (5.4 × 10^8^ cfu), *L. acidophilus* (4.5 × 10^8^ cfu), *L. plantarum* (4 × 10^8^ cfu) and *B. breve* (4 × 10^8^ cfu). - Oral - Single dose (2g) daily | 21 days | Baseline and end of treatment (day 21)  [VAS, BSS, IBS-SSS] | - Clinical improvement – decrease of at least 50 points in IBS-SSS score, decrease of more than 10mm in pain severity according to VAS, change in stool characterization to type 3 and type 4 according to Bristol stool chart | - Mean VAS scores, IBS-SSS total scores, & IBS-SSS sub-parameter scores of probiotic grp and placebo grp significantly reduced after treatment (p<0.001) - IBS-SSS scores for 86.04% (37 subjects) in probiotics grp and 85.71% (36 subjects) in placebo decreased by more than 50 points. - Both the groups were similar in terms of differences in VAS and IBS-SSS scores before and after treatment - Changes in stool (BSS) after treatment showed significant change in both groups - Rate of adherence to FODMAP restricted diet was 92% (90% in probiotics grp vs 94% in placebo grp) with no significant differences - Low FODMAP diet significantly relieved IBS symptoms in all IBS subtypes in the initial phase although adding probiotic to the diet did not provide additional pain relief. | Some concern |
| MinAh Jung  [2022] | Korea | Functional diarrhea | 22   - 10[intervention] - 12 [placebo] - Mean age   51.8 ± 14.9 [intervention]  50.2 ± 9.8 [placebo] | - *L. plantarum* CJLP243 - Oral - Single dose (2g powder) daily | 2 months | Baseline and end of study | - Adequate relief of FD symptoms (type 6 or 7 BSS decrease by more than 30% after 2 months compared to baseline - Effect on fecal microbiota and safety of *L. plantarum* CJLP243 - WBCs, Hb, ALT, BUN, creatinine | - The probiotic grp showed appropriate treatment response in 90% (9/10) while placebo grp showed 41.7% (5/12) with significant difference between the two groups - % of subjects with adequate FD relief (decrease in loose stool frequency) in the probiotic grp significantly increased after 2 months compared to baseline. - Probiotic grp showed significant reduction in log-transformed FC values compared with pre-treatment grp, whereas placebo grp showed no difference before and after treatment. - Levels of *Leuconostoc* genus organism in the gut microbiome increased significantly in probiotic grp at the end of treatment when compared to baseline levels. | Some concern |
| Maria Elisabetta Baldassarre  [2018] | Italy | Infantile colic in breast-fed infants | 53   - 27[intervention] - 26 [placebo]   Age 30-90 days old | Multi-strain probiotic mixture   - 4 LB strains (*L. paracasei DSM 24733, L. plantarum DSM 24730, L. acidophilus DSM 24735, and L. delbrueckii subsp. bulgaricus DSM 24734), 3 strains of bifidobacteria (B. longum DSM 24736, B. breve DSM 24732, and B. infantis DSM 24737*), 1 strain of *S. thermophilus* DSM 24731 - Oral - 10 drops of formulation [5 billion CFUs] once daily | 21 days | 7,14 and 21 days after beginning of administration of study product | - rate of responders and non-responders in improvement in colic symptoms, success rate defined as reduction of the daily average crying time ≥50% expressed in minutes - average crying time per day, parental quality of life, other GI events, anthroprometrical evaluations, side effects, amount of lactobacilli & bifidobacteria in stool samples in infants, metabolomics evaluation of feces by means of H-NMR | - higher and statistically significant rate of treatment success was found in probiotic grp at days 14 (p=0.04) and 21 (p<0.0001) - total average crying time was significantly less in probiotic compared to placebo throughout the 21 days (p<0.05) - parents of infants in the probiotic grp reported improved family QoL compared to placebo at days 14 [7.1 ± 1.2 - vs. 7.7 ± 0.9 (p = 0.02)] & 21 [6.7 ± 1.6 vs. 58.3 ± 1.0 (p = 0.001)]. - No differences were observed in bowel movements, stool consistency, fecal microbiota composition or anthroprometric data in probiotic vs placebo grp - Probiotics mixture administration appears safe and reduces inconsolable crying in exclusively breastfed infants | Some concern |
| Shin DY  [2020] | South korea | Rotaviral enteritis | 50   - 15[intervention] - 8 [placebo] - 27[retrospective data -medical records of patients who were hospitalized for rotaviral enteritis] - Children   mean age -36mo (probiotic), 14mo (placebo), 40mo (grp III) | - *L. plantarum*product LRCC5310 - oral | 1 week | Days 3-4, days 5-7 | - diarrhea, number of defecation & vomiting - Vesikari score – severity of enteritis - Secondary outcomes – virus multiplication - WBC absolute values, neutrophil count and CRP | - Statistically significant improvement was noted in the number of diarrheas, number of defecation events on day 3 and total diarrhea period in probiotics grp compared to placebo. (P = .033, P = .003, and P = .012, respectively) - improvement of Vesikari score in probiotics grp was greater than that in the other 2 groups (P = .076, P = .061, and P = .036, respectively) - 22.5% genotype 9 and 20% genotype 8 rotavirus strains were reported - Virus reduction effect was greater in probiotic grp compared to other grps - No significant side effects noted | High risk |
| Cheryl Stevenson  [2014] | South Africa | C-IBS constipation predominant -IBS &  D-IBS diarrhea predominant -IBS | 65   - 25[intervention] - 40 [placebo] - Mean age   48.15 ± 13.48 [intervention]  47.27 ± 12.15 [placebo] | - *L. plantarum* 299v   5 × 10^9^ cfu   - oral - 2capsules daily for 8wks | 12 weeks  [8wks followed by 2 weeks wash-out phase] | V1 (-2wk, beginning of run-in period)  V2 -baseline  V3 – V6 at 2,4,8 & 10 wks | - Decrease in abdominal pain at 8 weeks - Total QoL-IBS scores and individual symptom scores at the end of study compared to baseline - Severity of abdominal pain using Francis Severity Score questionnaire and changes in QoL assessed through questionnaire | - No statistically significant differences in abdominal pain relief [p=0.8] and QoL-IBS scores [p=0.687] was observed between two groups - Overall, 8-wk LP-299v probiotic therapy did not provide relief of symptoms [particularly abdominal pain and bloating] for pts with moderate IBS symptoms over placebo - Both groups had a significant improvement in abdominal pain scores during the study period indicating a large placebo effect [avg 251.55 to 197.9, p<0.0001] | High risk |
| S. Sen  [2002] | UK | IBS (untreated pts with IBS symptoms) | 12  Cross-over trial   - 12[intervention] - 12 [placebo] - 18-65yrs age | - *L. plantarum* 299v - oral - 5x10⁷ CFU/ml | 4 weeks | At the end of 4-wk placebo or probiotic therapy and after crossover for further 4-wks | - hydrogen & methane gas production - pain severity, stool urgency, abdominal distension, wind, stool and pain frequency - validated composite score and fermentation by 24-hr indirect calorimetry in a 1.4-m3 canopy followed by breath hydrogen determination for 3 hr after 20 ml of lactulose | - No significant differences between placebo and probiotic groups in median symptom score [8.5 vs 8], median maximum rate of gas production [0.55ml/min vs 0.92ml/min] & median hydrogen production [189.7ml/24hr vs 208.2ml/24hr] - breath hydrogen excretion was significantly reduced by the probiotic therapy compared to placebo [median at 120 min, 6 ppm vs 17ppm, p=0.019] - LP-299v treatment did not appear to alter colonic fermentation or improve symptoms in patients with IBS. | Some concern s |
| Valentina D’Onofrio  [2021] | Rome, Italy | Long-term home enteral nutrition (LTHEN) | 20   - 10[intervention] - 10 [placebo] - [average age: 75.2 ± 4.3 years] | Synbiotic product Syngut –   - *L. acidophilus 10^9^ billion* CFUs, 3.33x10^6^ *CFUs* of each - *B. lactis, L. plantarum, L. lactis* - Oral - One sachet/day | 4 months | Baseline T0 and end of treatment (after 4 months) T1 | - constipation and stool consistency - gut microbiota composition and its functional activity - biomarkers – methanobrevibacter, short chain fatty acids | - At the end of 4 months (T1), the intervention grp showed significant reduction in ‘composite scoring system’ CSS-value when compared to time point T0 - Significant reduction of constipation and increase of stool consistency in intervention grps were observed at T1 compared to control grp - biodiversity increased at T1 in 5/11 pts in intervention grp - methanobrevibacter was identified biomarker correlating to richness increase - Synbiotic intake was associated with increase in short chain FAs and reduction of harmful molecules | Some concern |
| Ahmet Sami Yazar  [2016] | Turkey | Infectious diarrhea | 110   - 55[intervention]Synbiotic grp - Zinc grp - 55 [Control grp] - Children age 6-120 months | Synbiotic preparation   - *L. casei, L. plantarum, L. rhamnosus, B. lactis* and prebiotics - Oral | 5 days | Day 3 and day 5 | - Duration of diarrhea (hours) - % of children with diarrhea during each day of intervention - Stool consistency [BSS] | - Duration of diarrhea was significantly reduced in the synbiotic grp and zinc grps compared to control grp (p<0.001) - At 72^nd^ & 96^th^ hours, % of children with diarrhea was lower in zinc grp compared to synbiotic (p<0.05 for both grps) - No significant difference in duration of diarrhea between synbiotic and zinc grps (p>0.05) - Zinc and synbiotic supplementation can reduce the duration of diarrhea with better clinical outcomes at 72^nd^ & 95^th^ hours and both therapies can be used in children with acute diarrhea | Some concern |
| Liu, Zhen-min  [2015] | China | Constipation | 120   - 60[intervention grp] - 60 [placebo grp] - Age 30-60yrs | Two strains   - *L. bulgaricus* and two strains of *S. thermophilus* - Oral (pasteurized yogurt) | 7 weeks | Follow-up every week | - Improvement in constipation symptoms, defecation frequency per week - Analyses of fecal pH & short chain fatty acids, microbiological analyses of feces - Biomarkers - short-chain fatty acids (Butyric acid, Propionic acid, Acetic acid) | - Significant increase in their defecation frequency following one week of the intervention - Constipation symptoms such as straining, lumpy or hard stool, and sensations of incomplete evacuation and anorectal blockage were all ameliorated - The numbers of fecal *Bifidobacteria* and lactobacilli, and the short-chain fatty acid concentrations increased significantly in the treatment groups. - Pasteurized yoghurt, with inactive LAB was found to be effective in improving constipation. The subjects who ingested pasteurized yoghurt showed a significant amelioration in their defecation frequency and constipation symptoms. Meanwhile, the numbers of fecal *Bifidobacteria* and lactobacilli, and the short-chain fatty acid concentrations increased. | Some concern |
| Vicente Lorenzo-Zúñiga  [2014] | Spain | Rome-III criteria for IBS with diarrhea | 84   - 29 [placebo grp] - 27 [low-dose intervention] - 28 [high-dose intervention] - Age 20-70 yrs | 1.31 probiotic combination -  3 strains (1:1:1)   - *L. plantarum (CECT7484 and CECT7485) and one Pediococcus acidilactici* (CECT7483). - High-dose - 1-3 × 10^10^ cfus/capsule - Low-dose - 3-6 × 10⁹ cfus/capsule - Oral - Once daily for both doses | 6 wks (42 days) | Dose related effect pre and post t/t  At baseline, mid-point (21d), end of trial (42d)  Symptom relief score- weekly avg of last 4 wks of t/t | - Improvement in health related QoL (IBS-QoL scale 1-100) - visceral sensitivity index scale to assess anxiety, symptom relief (5-point scale) | - IBS-QoL increased with treatment in all grps larger increment in pts treated with I.31 than placebo (p=0.008) - At 3 wks mean score increments significantly differed b/w grps -high dose probiotic grp (18±2), low dose probiotic (17±3) & placebo (12±2)[p=.017]. - At 6 wks both high [18±3] & low [22±4] achieved greater increments compared to placebo [p=0.023]. - Visceral sensitivity index, VSI measuring gut specific anxiety showed significantly greater improvement after 6 wks of t/t in patients with probiotic doses [high-10±2, low 14±2] vs placebo [7±1] [p<.05] acc. to IBS-QoL score - Symptom relief showed no significant changes between groups. - The combination of 3 different probiotic bacteria (I.31) was superior to placebo in improving IBS-QoL in patients with IBS and diarrhea. | Some concern |
| Marlene Wult  [2007] | Sweden | Pts having ongoing antibiotic associated diarrhea CDAD within last 2 months & +ve C. diff toxin assay within last 6 months of inclusion | 19   - 10[intervention] - 9 [placebo] - Age >18 yrs | - *L. plantarum*299v - Oral - 5 x 10¹⁰ CFU/day | 3 months | Pre and post t/t,  Toxin assays- Days 11-13, 37-41 & on telephone at day 70.  Fecal samples – 1, 11-31, 37-41 | - Clinical recurrence until day 70 - conc. Of SCFAs, lactate & succinate in fecal samples, identification of LP-299v in fecal samples of both grps | - Non-significant decrease in total organic acids. The fecal conc. of organic acids reduced significantly in placebo after intake of metronidazole and returned initial levels at day 36-41 (p=0.028). - Non-significant trend towards higher butyrate, total SCFAs and OAS in pt receiving LP-299v without recurrence in comparison to placebo with recurrence - Within placebo grp, individual SCFAs also decreased with antibiotic t/t [acetate p=0.05, propionate p=0.011, butyrate p=0.012] - In both grps, conc. Of succinate decreased significantly (p=0028) after cessation of antibiotics, and was unchanged during the initial intake of metronidazole - Significant differences in butyrate levels in feces between the two t/t grps during metronidazole t/t [p=0.047] - LP-299v reduces -ve influence of antibiotic therapy on colonic fermentation | Some concern |
| Bo Yang  [2021] | China | Pts with diarrhea ROME IV criterion, frequency of defecation ≥3 times/day | 55   - 28[intervention] - 27 [placebo] - Age 18-65yrs | - *L. plantarum*   CCFM1143  3.52 x 10⁹ CFU/day   - Oral - Once daily | 4 weeks | Pre- & post t/t  Baseline and 4^th^ week follow-up visit | - Abdominal symptoms, IBS-SSS, IBS-QoL, defecation frequency & stool status (BSS) - Neurobiological factors, motilin & cytokines in serum samples, SCFAs and gut microbiota in fecal samples | - After 4-wk of intervention, LP grp showed significant reduction in defecation frequency, Bristol scores indicating partial chronic diarrhea symptom relief - Alteration in immune response post t/t – significant increase in IL-6 & MTL level decreases in placebo grp. - Significant increase in acetic acid & propionic acid in intervention grp - Changes in gut microbiome – significant reduction in abundance of *Bacteroides* in LP grp, relative abundance of *Escherichia shigella* reduced significantly in LP grp compared to placebo. - LP reduced abundance of *Bacteroides* & *Eggerthella* while increasing *Akkermansia*, *Anaerostipes* & *Terrisporobacter* | High risk |
| Yang Liu  [2021] | China | Pt diagnosed with IBS-D symptoms based on ROME III criteria | 65   - 25 [intervention] - 20 [placebo] - 20 [oligosaccharides] - Age >18 yrs | - *L. plantarum*   CCFM8610   - Oral - Daily dose 2g | 12 weeks  [run in period 2 wks + intervention 8 wks + observation period 2 wks] | Pre- & post t/t  IBS-QoL scores measured at 3,7, & 11 wk  Fecal samples – wk 11 | - IBS-SSS (symptom severity scale), IBS-QoL at wk 3,7, 11 & changes in gut microbiota in fecal samples | - After 8-wk intervention, IBS-SSS score changed significantly. Total IBS-SSS scores decreased in LP grp. - Significant improvement in bowel habit satisfaction, bloating symptoms, life interferences scores was seen with LP supplementation whereas place grp showed bowel satisfaction in first 4 wks - Significant improvement in overall IBS symptom score (severe to moderate) in LP grp compared to both placebo & oligosaccharides - *Ruminococcus* and *Parabacteroides* significantly increased, relative abundance of butyric acid producing species changed in LP group. | Low risk |
| P. D. Kusumo  [2019] | Indonesia | Women with functional constipation | 36   - 15[intervention] - 21 [placebo] - Age18-60yrs | - *Lactiplantibacillus plantarum*IS 10506 - Oral - 10¹⁰ CFU/ml | 3 weeks | 3 time points  Baseline/interval/endline | - SCFA profile (acetate, propionate, butyric acid) | - After 21-day probiotic supplementation, SCFAs had a negative correlation with age & positive correlation with BMI - Probiotic supplementation had significant effect in change in percentage, change of delta SCFA (acetate, propionate & butyrate) - Probiotic supplementation significantly influenced acetate titer (p=0,032) marginally significant for propionate and butyrate (p=0.063 and p=0.068, respectively) - The respondent with increasing SCFA’s metabolite is higher in probiotic group compared to the respondents in placebo group - Acetate is the highest SCFA titer found in feces samples of women with functional constipation. | Some concern |
| R. Rosania  [2012] | Italy | Adult dyspeptic pts defined by Rome III specific criteria positive for H. pylori infection | 80   - 40[intervention] - 40 [placebo] - Age 24-76yrs - Mean age 52 | Mixture of 8 different probiotics   - *Streptococcus thermophilus, L. acidophilus,*   *B. longum, L. plantarum,*  *Bifidobacteriuym brevis, Lactobacillus paracasei,*  *Bifidobacterium infantis, Lactobaccilus delbrueckii*  *bulgaricus* subspecies: 1800 billions of alive bacteria   - Oral - Twice daily | 4 weeks | UBT was performed at baseline and end of 4wk therapy  [pts who received conventional therapy only underwent UBT 30 dys later] | - H. pylori status | - The eradication rate appeared to be independent by the baseline UBT delta value which did not differ between the two groups (39.5±19.3 versus 33.5±10.5) - probiotics showed a significant reduction of delta values (39.5±19.3 versus 12.5±8.7; p<0.005), whilst those treated with placebo demonstrated unchanged UBT results (33.5±15.5 versus 32.6±13.5) - non-significant difference was noted in the eradication of bacteria b/w 2 grps when conventional triple therapy was administered - probiotic supplementation has the ability to reduce bacterial intra-gastric load and it did not impact the efficacy of a subsequent conventional triple therapy - An adequate supplement with probiotic may help eradicate H.pylori | Some concern |
| M. Besselink  [2004] | Netherland | adult patients with first episode of predicted acute pancreatitis | 188   - 94[intervention] - 94 [placebo] - Age ≥18 yrs | Ecologic® 641 [6  strains of viable and freeze-dried bacteria]   - 4 lactobacillus strains *- L. acidophilus, L. casei, L. salivarius, L. lactis* - *2 bifidobacterium strains - B. bifidum, B. lactis* - Oral - Once daily dose of 10¹⁰ bacteria | 3 months | baseline & one follow-up visit at 3 months after discharge | - total no. of infectious complication, bacterial infection, infected, pancreatic necrosis, pneumonia, UTI reduced by probiotics - mortality, necrosectomy, use of antibiotics total hosp. stay, intensive care stay, side effects & abdominal complains SOFA scores, bacterial resistance | - The PROPATRIA study is aimed to show a reduction in infectious complications due to early enteral use of multispecies probiotics in severe acute pancreatitis | Some concern |
| A. McNicholl  [2018] | Spain | Adults naïve pts diagnosed with H. pylori infection within 12 mnths | 209   - 103[intervention] - 106[placebo] - Age 18-70 yrs | - Probiotic formula combining 2 strains *- L. plantarum & pedicoccus acidillatici*   1 x 10⁹CFU of each strain per capsule   - oral - one cap/day | 4-8 wks | 4 follow-up visits  screening (10-30 days before baseline visit), baseline, end of treatment/efficacy (10-15 days after baseline), follow-up (6±2wks after t/t completion | - mean total score of modified De Boer scale at the end of treatment - mean final score of individual items of De Boer scale, proportions of pts with occurrence of worsening of symptoms included in De Boer scale, frequency of spontaneity of reported adverse events | - Female gender (p<0.001) & quadruple therapy (p=0.007) were significant predictor of side effects independently - No significant differences were observed regarding side effects at the end of treatment between grps [p=0.7380] - No differences in compliance were observed between 2 grps. - Eradication rates were similar between the 2 grps no relevant differences in cure rates between triple and quadruple concominant therapy - Probiotic supplementation neither decreased side effects nor improved compliance with therapy or eradication rates. | Some concern |
| J Y Yoon  [2018] | Korea | pts diagnosed with IBS-C [iritable bowel syndrome-constipation or FC functional constipation] | 101   - 88 [intervention] - 83 [placebo] - Age 18-75 yrs | - probiotics- *S.* *thermophilus* (3 x 10⁸cfu/g*), L. plantarum* (1 x 10⁸cfu/g) - Oral (26g chocolate with probiotics) | 8 wk | 3 phases –  baseline period (1 wk)  treatment period (4wks)  wash out/ follow up (4 wks)  fecal samples - 0,4,8 wks | - stool consistency - BSFS scores, clinical symptoms - GIS, HR QoL, GSRS, CSBM - Other outcomes - gut microbiota in feces, conc. of IL-10,12, TNFa in serum | - Stool consistency measured by BSFS was significantly improved in the probiotic grp compared to placebo at 4- & 8-weeks [p=0.002] - the HRQoL was significantly better in the probiotic grp compard to placebo at 4 (p=0.044) & 8 weeks (p=0.049) - the relative abundance of L. Plantarum among fecal microbes was significantly greater in the probiotic grp cpmpared to placebo at 4 wks (p=0.029) - no significant differences in CSBM scores b/w two grps the levels of gut microbiota and serum cytokines (IL-10, IL-12 & TNFα did not differ significantly b/w 2 grps | Some concern |
| A. Saggioro  [2004] | Italy | Patients with IBS | 70   - 50[intervention]   24 [product A]  26 [product B]   - 20 [placebo] - Age 26-64yrs | 2 composite of probiotic products –   - Product A *- L. plantarum* + *B. breve* - Product B - *L. plantarum + L. acidophilus* - Oral (5 x 10⁹ CFU/ml of each in both product) | 4 wk | Baseline & end of study | - abdominal pain score [range 0-3], IBS symptom score [range 0-3] - severity of abdominal pain at different locations - severity of IBS symptoms (constipation, diarrhoea, bloating, flatulence, cephalea, nausea & dyspepsia) | - Pain scores in grp A & B decreased significantly by 42% & 49% at 14 & 28 days, whereas in placebo by 25% & 29.5% respectively - overall symptom score in grp A & B decreased from 49.3% & 56% at 14 & 28 days and in placebo by 8% & 14.4% respectively. - Short-term therapy with *L. plantarum* LP 01 and *B. breve* BR 03 or *L. plantarum* LP 01 and Lactobacillus acidophilus LA 02 may be considered a promising approach for IBS therapy | High risk |
| B. Mullish  [2021] | London | overweight & obese adults BMI-[25-34.9kg/m²] | 220   - 110 [intervention] - 110 [placebo] - Age 30-65 yrs | Lab4p probiotic –   - *L. acidophilus CUL60* - *L. acidophilus CUL21* - *L. plantarum CUL66* - *B. bifidum CUL20* - *B. animalis subsp. Lactis CUL34* - Oral (50 billion CFU) | 6 months | Baseline & end of study | - Onset of URTI symptoms, gut microbiota in feces - Other outcomes - anthroprometric measurements - BMI, body wt, waist circumference, waist-to-ht ratio, all favoring the probiotic arm | - Significant between group differences (27%) were observed in terms of self-reported URTI symptoms, favoring Lab4P grp [IRR=0.73, p<0.001] - significant interactions with symptom incidence were observed in regression analysis between treatment & age (p=0.007) & BMI (p=0.039) on stratification of the cohort - probiotics showed greater impact in >45yrs vs younger obese significantly p<0.001. - non-significant change in gut microbiota in stool samples of probiotic grp vs placebo | Low risk |
| Attilah Olah  [2007] | Hungary | patients with severe pancreatitis | 62   - 33[intervention] - 29 [placebo] - Age 19-81 yrs | Active synbiotic 2000[4 LAB +4 fibers]   - *Pediacoccus pentosaceus 5-33* - *Leuconostoc mesenteroides 77:1* - *L. paracasei ssp. F19* - *L. plantarum* 2362   10¹⁰ of each LAB strain, 40 billion per dose   - Enteral feeding dose gradually increased to 30kcal/kg | 7-10 days | Pre- & post t/t  on admission (baseline), 2-3rd day of t/t, after 7-10th day of t/t | - occurrence of bacterial infections assessed on CT-scans or USG fine needle aspiration or laprotomy samples – direct pancreatic tissue samples - routine lab studies, procalcitonin levels | - Significant differences were observed b/w 2 grps relative to total incidence of systemic responses syndrome (SIRS) & MOF [8 vs 14] - Lower rates of late organ failure were detected in the probiotic vs control grp [3% vs 17.2%] - non-significant differences were observed b/w the 2 grps relative to incidence of multiorgan failure (MOF), septic complications & mortality, was lower in probiotic grp vs control - Early nasojejunal feeding with synbiotics 2000 may prevent organ dysfunctions in the late phase of severe acute pancreatitis. The infection of pancreatic necrosis may be associated with early phase of organ failure | Some concern |
| B.S. Radomanska  [2021] | Poland | adults with IBS-D according to ROME III criterion | 48   - 25[intervention] - 23 [placebo] - Age 18-70 yrs | multi-strain probiotic – NordBiotic   - 4 B*ifidobacterium (breve, longum, bifidum, lactis)* - *5 Lactobacillus (rhamnosus, paracasei acidophilus, casei, plantarum)* - *S. thermophilus*   2.50 x 10⁹ cfu   - Oral - One capsule twice daily | 8 weeks | baseline, at 4 weeks of intervention, at 8 weeks of intervention | - changes in symptom severity & improvement (IBS-SSS & IBS- GIS scores) - changes in stool consistency (BFFS), no. of bowel movements per day, severity of pain, flatulence, fecal urgency, feeling of incomplete evacuation of stools, effects of intervention | - Significant improvement in IBS symptom severity (total IBS-SSS scores) in the probiotic grp compared to placebo (p=0.005), score relative to severity of pain (p=0.015), QoL (p=0.003) - no significant difference in occurrence of adverse events in both the groups - the probiotic preparation was safe, well-tolerated, and induced improvements in global IBS symptoms, symptom related severity of pain & QoL - multi-strain probiotic preparation containing *Lactobacillus*, *Bifidobacterium* & *S. thermopilus* is beneficial for adult IBS-D patients | Some concern |
| A. Tursi  [2010] | Italy | patient diagnosed with ulcerative colitis by previous colonoscopy consistent with histological & clinical diagnosis | 131   - 65[intervention] - 66 [placebo] - Age >18yrs | VSL#3 contains 900 billion viable/sachet lyophilized bacteria - 4LB+3B +1strep strains   - *L. plantarum, L. paracasei, L. acidophilus, L. delbrueckii ssp. Bulgaricus, B. longum, B. breve, B. infantis, S. thermophilus* - Oral - dose 3600 billion bacteria/day - 2 sachets/day | 8 weeks | Baseline & end of study | - evaluation of decrease in UCDAI by 50% or more at the end of 8 weeks - activity of relapsing UC remission UCDAI≤2 improvement in endoscopic scores, change in subjective symptoms, lack of beneficial effects of t/t | - Significantly higher number of patients had >50% decrease in UCDAI scores in the VSL#3 grp compared to placebo (PP p=0.010, ITT p=0.031) - Significant improvement of 3 points or more in the UCDAI scores (PP p=0.017, ITT p=0.046), rectal bleeding (PP p=0.014, ITT p=0.036) in the VSL#3 grp - 8 patients (11.2%) in the VSL#3 grp vs 9 (12.3%) reported mild side effects - non-significant improvement was seen relative to stool frequency, physicians rate of disease activity, endoscopic scores - can be considered as a safe effective adjunct for t/t of mild to moderate UC relapse and can help avoids or delays administration of steroids, immunosuppressants & other biologics | Some concern |
| C M Shin  [2021] | South Korea | patients with lower GI symptoms with abnormal bowel movements | 104   - 54[intervention] - 50 [placebo] - Age 18-80 yrs | ID-JPL934 probiotic preparation   - *L. johnsonii IDCC9203* - *L. plantarum IDCC3501* - *B. lactis* IDCC4301 - Oral capsules containing 1x10¹⁰ CFU of 3 live bacterial strains 1:1:1 ratio | 8 weeks | Baseline, 2-, 6- & 8-wk | - improvements in overall abdominal symptoms (BSFS) at wk 8 - change in stool form, consistency, frequency, & abnormal bowel, movement symptoms, quantitative change in fecal probiotic levels, changes in fecal microbiota | - Significantly higher overall symptom relief was observed in the probiotic grp compared to placebo p=0.016. - Significantly lower GI symptoms such as abdominal pain & bloating scores in probiotic grp compared to placebo p<0.05 - qPCR results showed significant increase in levels of *L. johnsonii* and *B. lactis* in feces post t/t (p<0.05) - No significant difference was observed in the overall fecal microbiome in both grps | Low risk |
| N. Bortoli  [2007] | Italy | patients diagnosed with H. pylori infection | 206   - 105 [standard triple therapy] - 101 [standard triple therapy] - Average Age 54.6±6.4 yrs | 100mg bLF capsule Dicofam   - *L. plantarum 5x 10⁹* - *L. reuterii 2x10⁹* - *L. casei ssp. rhamnosus 2x10⁹* - *B. infantis & B. longum 2x10⁹* - *L. salivarius 1x10⁹* - *L. acidophilus 1x10⁹* - *Strep. thermophilus 5x10⁹* - *L. sporongenes* 1x10⁹ - Preprobiotic - inuline - Oral - Probinul 5g/dose q.d. | 8 weeks | Baseline & end of intervention | - successful eradication – defined as negative C¹³ UBT aftr 8 wks | - Significant association of number of pts with high eradication rates with probiotic therapy ITT OR= 2.97, PP OR= 3.66 p<0.05 - 175/206 subjects showed negative C¹³ UBT - Significantly higher number of patients in standard therapy grp reported side effects from their t/t compared to standard + probiotics - Adding lactoferrin and probiotics to standard triple therapy significantly improves the efficacy of standard therapy (esomeprazole+ clarithromycin+ amoxycillin) for H. pylori infection - Results suggest beneficial synergistic action of bLf+Pbs for *H. pylori* eradication rate & reduction of side effects of antibiotics both during & after t/t adjuvant bovin lactoferrin + probiotics to classical standard triple therapy is valid more effective alternative to the standard H. pylori eradication protocol | Some concern |
| Irina A. Kirpich  [2008] | Russia | alcohol-related psychosis | 90   - 66 [intervention] - 24 [placebo] - Age >18 yrs | - *Bifidobacterium bifidum and Lactiplantibacillus plantarum*8PA3 - Oral - Once daily for 5 consecutive days | 7 days | Day 1 & day 7 | - Alteration in bowel flora - Serum biochemical tests - Plasma ALT, AST, LDH, and GGT activities, and total bilirubin | - Acoholic patients had significantly reduced numbers of *Bifidobacteria* (6.3 vs. 7.5 log CFU/g), lactobacilli (3.15 vs. 4.59 log CFU/g), and enterococci (4.43 vs. 5.5 log CFU/g) compared to placebo. - in the probiotic arm, *Bifidobacteria* (7.9 vs. 6.5 log CFU/g, p<0.05), lactobacilli (4.2 vs. 3.3 log CFU/g, p<0.05), and enterococci (5.3 vs. 4.65 log CFU/g, p<0.05) significantly increased from day 1 to day 7 - after probiotic therapy, alcoholic patients had significantly increased numbers of both *Bifidobacteria* (7.9 vs. 6.81 log CFU/g) and lactobacilli (4.2 vs. 3.2 log CFU/g) compared to standard therapy - The standard therapy group also had a significant reduction in AST from baseline to the end of therapy (28% reduction, 106.8 vs. 76.43 U/L) - probiotics had significantly lower AST and ALT activity at the end of treatment than those treated   with standard therapy alone (AST: 54.67 vs. 76.43 U/L; ALT 36.69 vs. 51.26 U/L) despite same values at initiation | Some concern |
| E. Lonnermark  [2010] | Sweden | Patients treated with infection who had started on antibiotics no more than 48 hrs with expected t/t period of 7-14 days | 163   - 83 [intervention] - 80 [placebo] - Age >16yrs | - *L. plantarum*299v - Oral - 200ml/day | 3 weeks | Pre- & post-treatment, baseline measurements after administration of 1^st^ dose & 7-10 days after termination of antibiotics | - proportion of pts developing diarrhea (at least 3 loose or watery stools /day) for at least 2 consecutive days - proportion of subjects +ve for c. diff toxin in feces after antibiotic t/t - risk of experiencing loose/watery stools, hard stools, abdominal pain, nausea, vomiting. Flatulence or blood in stools | - overall risk of developing loose or watery stools [OR=0.69, [0.52,0.92] p=0.012], nausea [OR=0.51, [0.30,0.85] p=0.0097] was significantly lower among those receiving LP299v - diarrhea >3 loose stools/24h for 2 consecutive days did not change significantly with t/t [OR=1.4, p=0.86] - no significant differences in carriage of c. difficile toxin b/w grps - daily intake of LP299v could have preventive effect on mild GI symptoms (reduce the incidence of loose stools & nausea) during antibiotic t/t with no significant t/t effect on diarrhea | Some concern |
| Guoxia Liu  [2020] | China | patients scored moderate level on Cohen's perceived stress scale (PSS-10) | 111   - 56 [intervention] - 55 [placebo] - Age 18-60 yrs | - *L. plantarum*DR7 isolated from fresh cow's milk | 12 weeks | Pre- & post-t/t  Pt completed questionnaire at baseline 4,8,12wks  Fecal samples at baseline & wk 12 | - occurrence of direct GI symptom - gut microbiota in fecal samples | - LP grp prevented an increase in frequency of defecation over 12 wk period compared to placebo (p=.044), modulating stress induced bowel movement - alpha diversity of gut microbiota was higher in LP grp after 12 wk (p=0.005) - beta diversity differed b/w grps at class & order (p<.001) - administration of DR7 changed levels of phyla of Bacteroides and Firmicutes and the classes of Deltaproteobacteria and Actinobacteria - correlation analysis revealed classes of Deltaproteobacteria and Actinobacteria. - Bacteroidetes, Bacteroidia, and Bacteroidales correlated with DBH & Bacteroidia and Bacteroidales correlated with TPH2 - administration of DR7 modulated stress-induced bowel movement by decreasing the frequency of defecation as compared to the placebo after 12 weeks. | Some concern |
| Irit Chermesh  [2007] | Israel | patient suffering from CD undergoing resection | 9   - 7 [intervention] - 2 [placebo] - Mean age 35.7±12.2yrs at surgery | Synbiotic 2000 is a mixture of prebiotic & probiotics includes 4 LAB+4 fibers   - 4 LAB- 1010 *Pediacoccus, pentoseceus, 1010 L. raffinolactis, 1010 L. paracasei susp paracasei 19, and 1010 L. plantarum* 2362 - Oral - Once daily | 24 months post-surgery | Pre- & post t/t  T/t began when oral fluid intake was resumed post-surgery  follow-up visits at 0,1,2,3mnths  then every 3-mnths until 24-mnths  post-surgery | - endoscopic and clinical examination relapse rate 3mnths post-surgery & at completion(24-mnths) - clinical parameters - wt, no. of bowel movements, abdominal pain (0-4scale) - labs – CBC, electrolytes, urea, creatinine, alanine & apartate aminotransferase, total protein, albumin, ESR, CRP | - After 3 months improvements in terms of wt gain, reduction in ESR, normalization of Hb levels & rise in albumin. Statistically significant improvement was seen for the rise of Hb & albumin levels at 1-yr post-surgery - No significant differences were observed in either clinical or endoscopic relapse examination rates b/w the 2 groups - No significant differences were observed in clinical & lab parameters - Synbiotic 2000 t/t has no effect on postoperative recurrence of pts with CD, larger sample studies with higher doses of probiotic cocktail should be assessed | Some concern |
| H-L Qin  [2008] | China | Acute pancreatitis | 74   - 38 [placebo] - 36 [intervention] - Age 25-75yrs | - *Lactobacillus plantarum* - Enteral feeding (nasojejunal tube) | 8 days | The intestinal permeability was assessed on the day of admission and on days 5 and 8. The rate of organ failure, septic complications and death cases were evaluated at the 8 day. | - development of infective complication. - non-infective complication, mortality and duration of hospital stay. - systemic inflammatory response syndrome (SIRS) and development of multiple organ dysfunction syndrome (MODS) and pulmonary complications | - 38.9% patients in the EIN group were colonized with multiple organisms compared to 73.7% in the PN group (P<0.01), and 30.6% patients in the EIN grew potentially pathogenic organisms compared to 50% patients in PN group (P<0.05). - The fecal bacterial DNA fingerprint profiles were less, the amount of *Lactobacteria* and *Bifidobacterial* decreased, and the number of *Enterococci* increased in PN group as compared with EIN group, P<0.05 - The lactulose/rhamnose ratio in EIN group were lower than that in PN group at days 5 and 8, P<0.05. The patients with LP got a better clinical outcome as compared with the patients with PN. - EIN (ecoimmunonutrition) enteral feeding can attenuate disease severity, improve the intestinal permeability and clinical outcomes. | Some concern |

**Table S6. Effect of *Lactiplantibacillus plantarum* in treating Gynecology**

| Author [Year] | Country | Disease/ health condition | Participants | Intervention | Intervention Duration | Follow ups | Outcome measurement | Study Findings | Quality Assessment |
| --- | --- | --- | --- | --- | --- | --- | --- | --- | --- |
| Per-Anders Jansson  [2019] | Sweden | lumbar spine bone loss in postmenopausal women | 232   - 116 [Control] - 116 [intervention]   Age [mean 59 years] | - intervention: *L. paracasei* DSM 13434, *L. plantarum* DSM 15312, and *L.*   *plantarum* DSM 15313   - Oral route - 1 capsule/ day | 12 months | month 1,3,6, and 12 | - DXA scan to assess LS-BMD | - *Lactobacillus* treatment reduced the LS-BMD loss compared with placebo (p=0·031) | Low risk |
| Marina Morato-Martínez  [2020] | Spain | Bone Loss in High-Risk Menopausal Women | 65   - 32 [control] - 33 [intervention]   Age 50 -60 years | - Intervention: *L. plantarum* 3547 + bioactive bone nutrients - Oral route | 24 weeks | Week 24 | - HDL-cholesterol - LDL-cholesterol triglycerides - Eelctrochemoluminesscence immunoassay kit (ECLIA) to determine CTX for bone resorption, and P1NP1 for bone formation. | - The intervention group showed a significantly increased bone mass compared to the Control group (p<0.05) - Increase levels of N-terminal propeptide of type I collagen (P1NP) bone formation marker in intervention group compared to control group (p<0.05) - Decreased carbo-terminal telopeptide of type I collagen (CTx) bone resorption marker compared to the Control group - Total cholesterol and LDL did not show significant differences between control and intervention. | Some concern |
| Anna Tomusiak [ 2015] | Poland | vaginal health status | 112   - 51 (Control) - 61 (Intervention)   Age 18–40-year-old | - *L. fermentum 57A* - *L. plantarum57B,* - *L. gasseri 57C.* - vaginal medicinal product in   gelatin capsules | 19 days with extension of 7 days due to menstruation. | four times during the study. | - Vaginal pH - Vaginal swab - Nugent score | Administration of the intervention contributed to a significant decrease (between visits) in both vaginal pH (P<0.05) and Nugent score (P<0.05), and a significant increase in the abundance of Lactobacillus between visit I and visits III and IV (P<0.05). | Low risk |

C-terminal telopeptide of type 1 collagen (CTX), procollagen type 1 N propeptide (P1NP), Bone mineral density (BMD)

**Table S7. Effect of *Lactiplantibacillus plantarum* in treating Hematologic Disease**

| Author [Year] | Country | Disease/health condition | Participants | Intervention | Intervention Duration | Follow ups | Outcome measurement | Study Findings | Quality Assessment |
| --- | --- | --- | --- | --- | --- | --- | --- | --- | --- |
| Axling, U.  [2020] | Sweden | Iron deficiency | 39   - 21 (control) - 18 (intervention)   Mean age is 22 years old (only female) | - *L. plantarum* 299v +   20 mg of iron (ferrous-fumarate)   - Oral capsule | 12 weeks | Week 4, 8 and 12 | - profile of Mod Score (POMS) - serum ferritin - plasma iron, total iron-binding capacity (TIBC) - reticulocytes, mean reticulocyte hemoglobin content (Ret-Hb) - Physical perfoemanc - hCRP | - The Profile of Mood States (POMS) questionnaire showed increased vigor with probiotic vs. iron alone after 12 weeks (3.5 vs. 0.1, p = 0.015). - Intake of probiotic with iron for four weeks increased ferritin levels more than iron alone (13.6 vs. 8.2 µg/L), but the difference between the groups was not significant (p = 0.056). - The mean reticulocyte hemoglobin content increased after intake of probiotic compared to control (1.5 vs. 0.82 pg) after 12 weeks, but the difference between the group was not significant (p = 0.083) - There were no significant changes over time or between groups in hemoglobin, plasma transferrin, plasma transferrin saturation, EVF, blood reticulocytes, sTfR, hepcidin, and hCRP levels. | Low risk |
| G. rosen  [2019] | U.S.A | Iron deficiency | 52   - 25 (control) - 27 (intervention)   Age 5-18 years | - *L. plantarum* 299v + Iron - Orally capsule | 8 weeks | Week 8 | - serum ferritin - C-reactive protein CRP - Reticulocytes - hemoglobin | - in separate linear regression models, significant association was seen between factors such as weight (p=0.0408) and iron dose (p=0.0226) with changes in ferritin levels - significant increase in ferritin levels in all patients in both groups p<0.001 - no significant differences in the increase in serum ferritin in children taking probiotic compared to controls. - non-significant association between probiotic use and increase in serum ferritin after controlling for factors such as child weight and dosing. | Some concern |

**Table S8. Effect of *Lactiplantibacillus plantarum* in treating Allergy**

| Author [Year] | Country | Disease/health condition | Participants | Intervention | Intervention Duration | Follow ups | Outcome measurement | Study Findings | Quality Assessment |
| --- | --- | --- | --- | --- | --- | --- | --- | --- | --- |
| Y. Nagata  [2010] | Japan | Allergy | 53   - Autumn groups   10 (control)  10 (intervention)   - Spring groups   17 (control)  16 (intervention)  Age 18-27 years F | - *L. plantarum* No. 14 - Oral consumption | 6 weeks | Week 6 and 7 | - nasal & ocular scores (0-4) - medication score (0-3) - symptom-medication score by Jap. Soc of Allergology - Th1 & Th2 in blood- flow cytometry | - Spring study - mean ocular SMS scores for the LP14 grp was lower than placebo during 1st week of intake period (p=0.003) in particular itchy eye symptom was lower in LP14 grp (p=0.014) - The Th1 % cells increased significantly in the intake period in LP14 (p=0.013, post-intake eosinophil counts increased significantly in placebo (p=0.018), score sensation after defecation in the LP14 grp after 3 weeks of intake was significantly higher in the placebo (p=0.047) - Autumn study - significant decline in the sensation scores after defecation was reported in Lp14 grp during post-intake period (p=0.04) | High risk |

**Table S9. Effect of *Lactiplantibacillus plantarum*in treating Neonates**

| Author [Year] | Country | Disease/health condition | Participants | Intervention | Intervention Duration | Follow ups | Outcome measurement | Study Findings | Quality Assessment |
| --- | --- | --- | --- | --- | --- | --- | --- | --- | --- |
| Ozge Serce Pehlevan  [2020} | Turkey | Very low birth neonates | 50   - 25 (control) - 25 (intervention)   Age 18-80 years 2 | - *820 million L. rhamnosus [Korean collection for type culture (KCTC) no 12202BP], 410 million L. plantarum [KCTC 10782BP, L. casei (KCTC 12398BP)], B. lactis (KCTC 11904BP), 383 mg fructooligosaccharide, 100 mg galactooligosaccharide, and 2 mg bovine lactoferrin* - Oral feeding with milk twice a day | 30 days | Week 2 and 4 for serum levels.  The serum cytokine levels were determined.  at postnatal 0 ± 2, 14 ± 2, and 28 ± 2 days. | solid-phase sandwich enzyme-linked immunosorbent assay | - The IL10 levels decreased significantly throughout the study period in the probiotic group (p=0.011). - The decrease in IL-5 levels from day 14 to day 28 in the probiotic group was significant (p=0.042). - In the control group, the serum levels of IL-5 increased significantly within the first 14 days (p=0.019) but decreased on day 28 (p=0.011). | Some concern |

**Table S10. Effect of *Lactiplantibacillus plantarum* in treating Pulmonology disease**

| Author [Year] | Country | Disease/health condition | Participants | Intervention | Intervention Duration | Follow ups | Outcome measurement | Study Findings | Quality Assessment |
| --- | --- | --- | --- | --- | --- | --- | --- | --- | --- |
| C. Koning  [2010] | Netherlands | Chronic Obstructive Pulmonary disease  COPD | 30   - 13 (control) - 17 (intervention)   Age18-80 years | - *B. bifidum,*   *B. lactis,*  *E. faecium, L. acidophilus, L. paracasei, L. plantarum, L. rhamnosus, L. salivarius*   - oral consumption | 63 days | Day 7,14 and 63 | - quantitative PCR - DGGE based similarity index - during & after | - DGGE analysis showed no difference in band richness between the probiotic and the placebo groups or over time within both the groups was found. - No differences in the 16S rRNA gene copy numbers of total bacteria, lactobacilli or bifidobacterial were observed between the probiotic and the placebo groups. | High risk |

**Table S11. Effect of *Lactiplantibacillus plantarum*in treating Nephrology disease**

| Author [Year] | Country | Disease/health condition | Participants | Intervention | Intervention Duration | Follow ups | Outcome measurement | Study Findings | Quality Assessment |
| --- | --- | --- | --- | --- | --- | --- | --- | --- | --- |
| Fortes PM  [2020] | Brazil | Nephrotic disease | 4   - 2 (control) - 2 (intervention)   Age 2-17 years | - *L. plantarum,* strain Lp-G18 - Oral capsule | 12 weeks | Every 45 days | - enzyme-linked immunosorbent assay (ELISA) | In the probiotic group there was a tendency to reduce TNF-α levels and increase IL-10 levels when compared to controls. Regarding the lipid profile, there was a decrease in serum triglyceride (6.0 mg / dL) and total cholesterol (41.5 mg / dL) levels in the probiotic group when compared to baseline levels, while in the control group there was an increase in serum triglyceride (49.5 mg / dL) and total cholesterol (8.0 mg / dL) levels, respectively | Some concern |
| I. K. Wang  [2015] | China | Nephrotic disease | 39   - 18 (control) - 21 (intervention)   Older than 18 years of age | - *L. plantarum A87, B. bifidum, B. catenulatum, B. longum.* - Oral capsule | 6 months | Month 6 | - serum endotoxins - immunoenzymatic ELISA test | - in probiotic grp serum levels of TNF-α, IL-5, IL-6 and endotoxins significantly reduce after 6 months of treatment. - Serum IL-10 levels increased significantly in the probiotic group, residual renal function significantly declined in the placebo grp after 6 months and was preserved in probiotic group. - no significant changes in the levels of serum cytokines & endotoxins in the placebo grp after 6 months of treatment | Some concern |

**Table S12. Effect of *Lactiplantibacillus plantarum* in treating Mental health**

| Author [Year] | Country | Disease/health condition | Participants | Intervention | Intervention Duration | Follow ups | Outcome measurement | Study Findings | Quality Assessment |
| --- | --- | --- | --- | --- | --- | --- | --- | --- | --- |
| Chang Chun  [2021] | Taiwan | Tourette Syndrome | 57   - 29 [control] - 28   [intervention]  Age 5-18 years | - Intervention: *L. plantarum* PS128 - Oral capsules   1 capsule twice daily | From 1 August 2017 through to 31 Jan 2019 | Month 1 and 2 | - Yale Global Tic Severity Scale (YGTSS) - ADHD-SNAP-IV Scale - ADHD-Conners’ Continuous Performance test II (CPT-2) - OCD-Obsessive-Compulsive Inventory-Revised (OCI-R) - Migrain Disability Assessment (MIDAS) - Children’s Depression Inventory   (CDI-TW) | - No significant improvement in YGTSS between Placebo and Intervention. And no superior effects of intervention over placebo over time. - SNAP-IV scores significantly reduced from parent's evaluation in probiotic grp vs placebo in terms of total score (p=0.021), inattention score (p=0.005), hyperactivity (p=0.027) - CDI paired t-test showed reduced scores in Placebo group (p-value= 0.001).   Neither group showed significant improvement in OCI-R and MIDAS | Some concern |
| Yun-Ha Hwang  [2019] | Korea | Mild cognitive impairment (MCI) | 100   - 50 [control] - 50 [intervention]   Age 55-85 years | - *L. plantarum* C29-fermented soybean (DW2009) - Oral route, 1 capsule Twice daily | 12 weeks | Week 6 and week 12 | - The computerized neurocognitive function tests **(CNT)** - the verbal learning test **(VLT)** - auditory continuous performance test **(ACPT)** - digit span test **(DST)** - brain-derived neurotrophic factor **(BDNF)** | - probiotic group showed greater improvements in the combined cognitive functions (z=2.36, p for interaction = 0.02), especially in the attention domain (z=2.34, p for interaction =0.02) compared to placebo. - Cognitive improvement was associated with increased serum BDNF levels in probiotic group (t=2.83, p=0.007) - There was no significant association between change in serum BDNF levels and cognitive performance in the placebo. | Some concern |
| Eva Román  [2019] | Spain | Cognitive dysfunction | 35   - 18 [control] - 17 [intervention] | - *Intervention: strains, namely, S. thermophilus, B. breve, B. longum, B. infantis, L. paracasei ,L. acidophilus , L. delbrueckii subsp bulgaricus , and L. plantarum* - Oral rout - 450 billion bacteria twice daily | 12 weeks | Week 6 and 12 | - Psychometric Hepatic Encephalopathy Score **(PHES)** to assess cognitive function - Time Up and Go scale **(TUG)** to assess risk of falling - **Gait speed score** to assess risk of falling | - The intervention group showed an improvement in the PHES (P = 0.006), TUG time (P = 0.015) and gait speed (P = 0.02). - Lower incident of falls was observed during follow-up among the intervention group compared to placebo. | Low risk |
| Parracho  [2010] | Belgium | Autistic spectrum disorder | 17   - 9 [group I] - 8 [group II]   Age 3- 16years | Intervention: L. plantarum WCFS1, Oral Capsules, dosage, frequency  Placebo: None  Group I: 3 weeks placebo + 3 weeks Intervention  Group II: 3 weeks Intervention + 3 weeks placebo | 3 weeks | - Week 3 and 12 | - Total Behavior Problem Score (TBP-S) - **GI symptoms** | - The TBPS and GI symptoms were not significantly different between the intervention and control periods. - Intervention resulted in significantly higher Lab158 counts, and significantly lower Erec482 counts, compared to placebo in microbiologic analysis (p<0.05). | High risk |
| Xue-Jun Kong  [2021] | USA | Autistic spectrum disorder | 35   - 17 [control] - 18 [intervention]   Age 3-25 years | *Intervention: L. plantarum* PS128, 2 capsules/day (check frequency) + oxytocin  Control:  Placebo pill + oxytocin | 28 weeks | Week 16 and 28 | - GI severity index **(GSI)** - Social Responsiveness Scale **(SRS)** - Aberrant Behavior Checklist **(ABC)** - Clinical Global Impression **(CGI)** | - More improvement in ABC, SRS, and CGI among the intervention, compared to the control group (p < 0.05) - No significant difference regarding GSI between the intervention and control group (p>0.05). | Low risk |
| Liu, Yen-Wenn  [2019] | Taiwan | Autism spectrum disorder  (ASD) | 71   - 35 [control] - 36 [intervention]   Age 7-15 years | - *Intervention: L. plantarum* PS128 - Oral rout   The probiotic capsules weighed 425 ± 25 mg and contained 3 ×10^10^ CFU/capsule of PS128 | 4 weeks | Week 4 | - changes in the Autism Behavior Checklist-Taiwan version **(ABC-T)** questionnaire - the Social Responsiveness Scale **(SRS)** scores - the Child Behavior Checklist **(CBCL)** questionnaire - Clinical Global Impression-Severity **CGI-S** and **CGI-I** questionnaires - Nolan and Pelham **(SNAP)-IV-**Taiwan version | - The CGI-I scores for both intervention and placebo were equivalent to minimally improved   P = 0.94   - There was no difference between the placebo and the intervention groups in the total ABC-T-score and subscale scores both on baseline and week 4, except for body and object use in the intervention group showed a trend to reduce the scores compared to placebo p = 0.04. - exploratory analysis showed nominal reduction of SRS-total score of intervention group between baseline and week 4 (P = 0.04), the children in the placebo control group did not show improvements over time - (CBCL), four-week intervention showed nominally reduced the scores for anxiety (P = 0.02), and rule-breaking behaviors (P = 0.02). Children in the placebo group showed a reduction in scores for problems related to externalization (P = 0.02) where intervention group did not. - (SNAP)-IV, intervention group showed improvement by reduced total scores (P = 0.01), hyperactivity and impulsivity (P = 0.04), and opposition and defiance (ODD; P = 0.045) where placebo group did not exhibit these changes | Low risk |
| Elisa Santocchi  [2020] | Italy | Autism spectrum disorder  (ASD) | 63   - 32 [control] - 31 [intervention]   Age 18- 72 months | - Intervention: S. thermophilus, B. breve, B. longum, B. infantis, Lactobacillus acidophilus, *L.* plantarum, *L.* para-casei, *L.* delbrueckii subsp. bulgaricus. - Each packet contained 450 billion of bacteria - Oral rout - 1^st^ month: 2 packets/ day; Then 1 packet/ day for 5 months | 6 months | Month 6 | - Total ADOS Calibrated Severity Score **(ADOS-CSS)** to ass autism severity - Social-Affect **(SA)** ADOS-CSS - Restricted Repetitive Behaviors **(RRB)** ADOS-CSS - Vineland Adaptive Behavior Scales-Second Edition **(VABS-II): CBCL, PSI, Linguistic level** | - No significant differences were observed between intervention and placebo group in Total ADOS-CSS, VABS-II, CBCL, PSI or Linguistic level scores. | Some concern |
| Yu-Ting Ho  [2021] | Taiwan | insomnia | 40   - 19 [control] - 21 [intervention]   Age 20-40 years | - *Intervention: L. plantarum* PS128 - two capsules of either PS128 or Placebo after dinner for 30 days | 30 days | Day 15 and 30 | - Hear Rate variability **(HRV)** - electroencephalogram index **(EEG)** - relaxation level, fatigue level, and sleep quality **(VAS) scale** - The Pittsburgh Sleep Quality Index **(PSQI)** - Insomnia Severity Index **(ISI)** - Epworth Sleepiness Scale **(ESS)** - the Beck Depression Inventory-II score **(BDI-II)** - the Beck Anxiety Inventory **(BAI)** - the State-Trait Anxiety Index **(STAI)** | - No difference was observed between the placebo and Probiotic groups regarding PSQI, ISI, ESS, and BAI scores and HRV values. - More improvement in BDI-II among the intervention, compared to the placebo group. - On EEG the Intervention group showed significant decrease in awakenings compared to placebo. - The VAS fatigue level before sleep indicated that the control group felt significantly more tired on day 30 than on day 0, and that there was a significant difference between the two groups. | Some concern |
| Rajesh Venkataraman  [2021] | India | Anxiety and Depression | 74   - 38 [control] - 36 [intervention]   Age 18-24 years | - Intervention: *B. coagulans Unique IS2, L. rhamnosus UBLR58, B. lactis UBBLa70, L. plantarum UBLP40, B. breve UBBr01, B. infantis UBBI01* - Oral capsules twice a day | 28 days | Day 28 | - stress scale **(PSS)** - depression anxiety stress scale **(DASS)** - state-trait anxiety inventory **(STAI)** questionnaire. | - Compared to placebo, the intervention group showed significant reduction in PSS, DASS, and STAI scores from baseline, p valueα < 0.001. | Some concern |
| G. Onning  [2020] | Sweden | Stress | 63   - 31 [control] - 32 [intervention]   Age 19 – 35 years | - Intervention:   *L. plantarum LPHEAL9*   - Oral capsules - 1 capsule / day | 4 weeks | Week 4 | - Shirom-Melamed burnout questionnaire score **(SMBQ)** - Sielberger state and trait anxiety inventory scale **(STAI-S)** - Cortisol levels and inflammatory markers - Heart rate **(HR)** - High frequency heart rate variability **(HF-HRV)** | - After 4 weeks of intervention, the level of lactobacilli in saliva was significantly higher in intervention group compared to the placebo (p < 0.0001). - No significant difference between the groups were found in both (SMBQ)   And (STAI-S)   - No significant difference found in the cortisol levels between groups. - no differences were observed in fractalkine levels & group condition interaction. - No significant differences between the groups (intervention and placebo) were found for HR and HF-HRV. | Some concern |
| A. Reiter  [2020] | Austria | Major Depressive Disorder (MDD) | 61   - 33 [control] - 28 [intervention]   Age 18-75 years | - Intervention: *L. casei W56, L. acidophilus W22, L. paracasei W20, B. lactis W51, L salivarius W24, L. lactis W19, Bifidobacterium lactis W52, L. plantarum W62, B.bifidum W23* - Oral route - Once daily | 4 weeks | Week 4 | - gene expression - quantitative real time PCR analysis - interleukin-6 (IL-6) - nuclear factor kappa B Subunit 1 **(NFKB1)** - Tumor necrosis factor (TNF) - **HAMD** - Hamilton depression scale - **BDI-II** - Beck depression inventory -II score | - IL-6 gene expression levels increased in placebo and decreased in intervention group. - At univariate level, for IL-6, the interaction effect (time*group) was significant (p=0.022) - Both intervention and placebo showed significant improvement in psychological parameters over time (p<0.001; BDI, p<0.001; HAMD= 47.85,P<0.001) - No significant effect was found in TNF, nor NFKB1 on universal level. | High risk |
| P. Gualtieri [2020] | Italy | anxiety disorders | 142   - 71 [control] - 71 [intervention]   Age 18-65 years | - *Streptococcus thermophiles, Bi. animalis, B. bifidum, Streptococcus thermophiles, L. bulgaricus, L. lactis subsp. Lactis, L. acidophilus, L. plantarum, L.reuter* - Oral Suspension   Once daily | 12 weeks | Week 12 | - Hamilton Anxiety Rating Scale **(HAM-A)** - Body Uneasiness Test **(BUT) self-reported questionnaire** - **Symptom Checklist** 90-Revised **(SCL90R).** - Interleukin 1 Beta Genotyping. | - The percentage of anxious patients was reduced more in the intervention (Δ% = −10:64%) than in the Placebo (Δ% = −5:10%). - HAM-A total score was reduced more the intervention than in the Placebo (β = −0.33; p < 0.01; OR = 0.68 (0.40; 1.15). - BUT and SCL-90 questionnaire results did not show significant changes after probiotic treatment compared to placebo. - Intervention was not effective in reducing anxiety risk in non-carriers. - Probiotic treatment determined a significant reduction of anxiety risk in A carriers (β = −0.32; p = 0.02; OR = 0:.3 (0.56; 0.94)) - no statistically significant difference (p ≥ 0:05) for total BUT score, BUT GSI score, total SCL-90R score, and SCL90R GSI score. | High risk |
| H.X. Chong  [2019] | Malaysia | stress and anxiety | 111   - 56 [control] - 55   [intervention]  Age 18 – 60 years | - Intervention:   *L. plantarumDR7*   - Each aluminum sachet consisted of 2 g | 12 weeks | Week 4, 8 and 12 | - psychological distress using the Perceived Stress Scale **(PSS-10**) - Depression, Anxiety and Stress Scale **(DASS-42)** - CogState brief batter **(CBB)** to assess memory and cognitive functions - Plasma cortisol level - Pro inflammatory cytokines **(IFN-γ and TNF-α)** - Anti-inflammatory (interlukin 10) | - The intervention reduced symptoms of stress (P=0.024), anxiety (P=0.001), and total psychological scores (P=0.022) as early as 8 weeks among stressed adults compared to the placebo group as assessed by the DASS-42 questionnaire. - Plasma cortisol level was reduced among intervention subjects as compared to the placebo, accompanied by reduced plasma pro-inflammatory cytokines, such as interferon-γ and transforming growth factor-α and increased plasma anti-inflammatory cytokines, such as interleukin 10 (P<0.05). - The intervention improved cognitive and memory functions in normal adults (>30 years old), such as basic attention, emotional cognition, and associate learning (P<0.05), as compared to the placebo and young adults (<30 years old). | Low risk |
| Alberto Saccarello  [2020] | Italy | Depression | 87   - 44 [control] - 43   [intervention]  Age 18-60 years | - Intervention: *L. plantarum HEAL9 +*   Oral tablet  SAdenosylmethionine  (SAMe 200 mg) | 6 weeks | day 14 ± 2 and week 6 | - ZUNG SELF-RATING DEPRESSION SCALE (Z-SDS) - Zung Self-Rating Anxiety Scale (Z-SAS) - Insomnia Severity Index **(ISI)** - Birmingham IBS symptom questionnaire (B-IBS) - European Quality of Life 5 Dimensions 3 Level Version (EQ-5D-3L) | - The combination of SAMe and probiotic showed a greater reduction in the Z-SDS total score (P = .0165) and the core depression subdomain (P = .0247) compared to placebo. - A significant reduction in favor of the combination was shown at treatment week 2 for the Z-SDS total score (P = .0330), the cognitive and anxiety subdomains (P = .0133 and P = .0459, respectively), and the anxiety questionnaire (P = .0345). | Some concern |
| Leszek Rudzki  [2019] | Poland | Depression | 60   - 30 [control] - 30   [intervention]  Mean age for placebo 39.13, for intervention 38.90 years | - Intervention:   *L. Plantarum 299v (LP299v) +* SSRI   - Placebo + SSRI | 8 weeks | Week 8 | - Pro inflammatory cytokines **(TNF-α)** - Plasma cortisol level - Kynurenines level - psychological distress using the Perceived Stress Scale (PSS-10) - Hamilton depression scale **(HAM-D 17)** - Symptoms checklist-90 (**SCL-90)** | - No significant changes in treatment x time effect in outcome measures such as HAM-D 17, SCLE-90 and PSS-10. - The intervention group showed an improvement in some affective, cognitive characteristics such as APT and CVLT total recall of trials 1-5 compared to placebo. - significant increase in 3HKYN:KYN ratio in the intervention group compared with the placebo group. - no significant changes of TNF-α, IL-6 and IL-1b and cortisol concentrations in neither probiotic nor placebo groups. | Some concern |
| F. Martami  [2019] | Iran | Migraine | 40   - 18 [control] - 22   [intervention]  Age  18-60 years | - Intervention: *B. subtilis, B. bifidum, B. breves, B. infantis, B. longum, L. acidophilus, L. delbrueckii ssp. bulgaricus, L.casei, L. plantarum, L. rhamnosus, L. helveticus, L.salivarius, L. lactis ssp. Lactis, S.*   *thermophilus*   - 2 Oral capsules per day | - 10 weeks for episodic Migraine (EM)  - 8 weeks for Chronic migraine (CM) | Week 10 | - Migraine severity - **VAS scale.** - C-reactive protein (CRP) - Tumor Necrosis Factor alpha   **(TNF-α)** | - In EM the mean frequency of migraine attacks significantly reduced in the probiotic group compared to the placebo group (mean change: -2.64 vs. 0.06; respectively, p<0.001). - Migraine severity significantly reduced (mean decrease: -2.14 in the probiotic group and 0.11 in the placebo group; p<0.001). - The intervention group showed significant reduction in abortive drug usage per week (mean change: -0.72; p<0.001) compared to baseline, while there were no significant changes within the placebo group. - In CM the mean frequency of migraine attacks significantly reduced in probiotic compared to placebo (mean change: -9.67 vs. – 0.22; p≤ 0.001). - In contrast to the placebo, probiotic supplementation significantly decreased the severity (mean changes: -2.69; p≤0.001), duration (mean changes: -0.59; p≤0.034) of attacks and the number of abortive drugs taken per day (mean changes: -1.02; p<0.001), in chronic migraine patients. - No significant differences in the serum levels of inflammatory markers in CM and EM at the end of the study | Some concern |

polymerase chain reaction (PCR), autism spectrum disorder (ASD), the visual analogue scale **(VAS)**

**Table S13. Effect of *Lactiplantibacillus plantarum* in treating Obesity**

| Author [Year] | Country | Disease/health condition | Participants | Intervention | Intervention Duration | Follow-ups | Outcome measurement | Study Findings | Quality Assessment |
| --- | --- | --- | --- | --- | --- | --- | --- | --- | --- |
| Juscelino Tovar  [2012] | Sweden | overweight | 88   - 44 [control] - 44 [intervention]   Age 50 and 73years | - Active diet including (*L. plantarum*Heal19/DSM1531 - Each diet was consumed for 4 weeks with a 4 weeks washout period. | 8 weeks | four clinical visits, one before and one after each intervention period | - Chemiluminescent immunometric assay - Sensitive enzyme-linked immunosorbent assay - ELISA - Enzymatic colorimetric assay | - Intervention showed significant differences in total serum cholesterol (-26 ± 1% vs baseline; P < 0.0001), LDL-cholesterol (-34 ± 1%; P < 0.0001), triglycerides (-19 ± 3%; p=0.0056), LDL/HDL (-27 ± 2%; P < 0.0001), apoB/apoA1 (-10 ± 2%; P < 0.0001), HbA1c (-2 ± 0.4%; p=0.0013), hs-CRP (-29 ± 9%; p=0.0497) and systolic blood pressure (-8 ± 1%¸ P = 0.0123). - the Framingham cardiovascular risk estimate was 30 ± 4% (p<.0001) lower and the Reynolds cardiovascular risk score, which considers CRP values, decreased by 35 ± 3% (p< 0.0001). | Some concern |
| Yusuke Tanaka  [2020] | Japan | (BMI) between 23 and 30 | 96   - 47 [control] - 49 [intervention]   Age 20 to 75 years | - Heat-killed *L. plantarumL-137 (HK L-137)* - One tablet /day containing HK L-137 (10 mg) | 12  weeks | every 4 weeks | - Enzyme-linked immune   sorbent assay ELISA | - Intervention showed significant decreases in aspartate aminotransferase (AST) and alanine aminotransferase (ALT) compared to the control group (p=0.02 and p=0.02, respectively) - Significant decrease in the leukocyte count (p=0.08) in control group. - The pulse rate, serum calcium level, and platelet count were significantly higher in the HK L-137 group than in the control group, while total bilirubin and blood urea nitrogen were significantly lower - Significant decrease of TC (total cholesterol) and LDL-C(low-density lipoprotein cholesterol) in the intervention group at 12 weeks. - There was no significant difference in BMI between the control group and the HK L-137 group during the study period. | Some concern |
| Minkyung Kim  [2017] | South Korea | non-diabetic and overweight individuals | 95   - 46 [control] - 49 [intervention]   Age 20 to 75 years | - *L. curvatus* HY7601 and *(L. plantarum*) KY1032 - 2 g of probiotic powder twice a day | Over 12  weeks | weeks 0, 6 and 12 | - Ultra-performance liquid chromatography - Mass spectrometry - computed   tomography (CT)   - Dual-energy X-ray absorptiometry (DEXA) | - Intervention showed significantly increased the levels of octenoylcarnitine (C8:1), tetradecenoylcarnitine (C14:1), decanoylcarnitine (C10) and dodecenoylcarnitine (C12:1) compared to control group - In the probiotic group, the changes in the body weight, body fat percentage, body fat mass and L1 subcutaneous fat area were negatively associated with changes in the levels of C8:1, C14:1, C10 and C12:1 acylcarnitines. - No significant differences were observed in the metabolites at the baseline between the placebo and probiotic groups. | Low risk |
| Sudha, M. R.  [2019] | India | Obesity | 70   - 35 [control] - 35 [intervention]   Age 30‐65 years | - (UB0316: *L. salivarius UBLS-22, L. casei UBLC-42, L. plantarum, UBLP-40, L. acidophilus UBLA-34, B. breve UBBr-01, B.* coagulans Unique IS2 5×10^9^ cfu each and fructo-oligosaccharide, 100 mg) - 2capsules/day | 12 weeks | baseline assessments, visit 1 (week 4), visit 2 (week 8), and visit 3 (week 12) | - BMI was calculated electronically using an HBF-362 - Karada scan (Omron, Hoofddorp, the Netherlands) body fat monitor - Manually on readings of weight and height. | - Intervention significantly reduced BMI (95% CI: ‐0.64, ‐0.27; P=0.0001), body weight (95% CI: ‐1.16, ‐0.50; P<0.0001), and WHR (waist-to-hip ratio (95% CI: ‐0.06, ‐0.01; p=0.007) from the baseline, compared to placebo. - Fat, blood lipid and sugar profile changes were non‐significant | Some concern |
| [Hemalatha Rajkumar](https://pubmed.ncbi.nlm.nih.gov/?term=Rajkumar+H&cauthor_id=24795503)  [2014] | India | Obesity | 60   - Control 2 groups each one 15 - Intervention 2 groups each one 15 (probiotic VSL#3, or both omega-3 and probiotic)   Age 40-60 years | - probiotic (VSL#3) and omega-3 fatty acid - One capsule of each intervention /day | 6  weeks | Baseline and after 6 weeks | Blood and fecal samples to measure total cholesterol, triglyceride, LDL, and VLDL, HDL, insulin sensitivity, the composition of gut microbiota, Inflammatory markers | - Intervention determined a significant reduction in total cholesterol, triglyceride, LDL, and VLDL and had increased HDL (P<0.05) value. - improved insulin sensitivity (P<0.01) - Decreased hsCRP - Addition of omega-3 fatty acid with VSL#3 had more pronounced effect on HDL, insulin sensitivity, and hsCRP. | Some concern |
| Rahayu, E. S. [2021] | Indonesia | Obesity | 60   - 30 [Control] - 30 [Intervention]   Age 35 - 56 years | - *L. plantarum Dad-13* - *1 g of skimmed* *milk powder* *containing the probiotic L. plantarum of 2 × 10^9^ CFU in sachet packing* | 90 days | day 10 + 1(the end of the baseline period and the start of the ingestion period), and the ingestion period (from day 11 to day 101) | - GM analysis, - SCFA (Short-chain fatty acids) analysis using gas chromatography - The measurement of body weight and height - The lipid profiles - The measurement of fecal pH using a pH meter | - Intervention determined a significant decrease in body weight and BMI (p<0.05) - There were no significant changes in lipid profile, SCFAs (e.g., butyrate, propionate, acetic acid) | Some concern |
| Eun-Ji Song  [2020] | Republic of Korea | Obesity | 43   - 22 [Control] - 21 [Intervention]   Age 20 – 60 years | - *B. breve CBT BR3, L.* *plantarum* CBT LP3 - Two capsules per day after breakfast | 12 weeks | every 3 weeks. | - Total energy consumption and nutrient intake were calculated using CAN-Pro 4.0 nutrient analysis software (The Korean Nutrition Society, Republic of Korea) - Body weight and height were measured with an automatic scale (G-tech, Uijeongbu, Republic of Korea) - Body composition measurements were determined using InBody 3.0 (Biospace, Seoul, Republic of Korea) - a bioelectrical impedance analysis device to measure fat percentage, fat mass, and lean body mass. | - Intervention determined a significant decrease in waist circumferences (mean difference of 1.88 cm, p = 0.049) - Internal fat area and the ratio of visceral to the subcutaneous fat area (V/S ratio) significantly decreased in both groups - Internal fat area decreased by an average of 6.47 cm2 in the probiotic group (p<0.001) and 4.98 cm2 in the placebo group (p=0.021). - The V/S ratio dropped by 0.022 in the probiotic group (p<0.001) and 0.015 in the placebo group (p=0.013). - The pulse rate of the probiotic group significantly decreased (mean difference of 3.4, p= 0.009). | Some concern |
| [Minji Sohn](https://pubmed.ncbi.nlm.nih.gov/?term=Sohn+M&cauthor_id=35126309)  [2022] | South Korea | Obesity | 71   - 36 [Control] - 35 [Intervention]   Age 20 – 65years | - *L.* *plantarum K50* - Capsules   (4 × 109 CFU/day) | 12 weeks | from the baseline to 12 weeks | - Dual-energy X-ray absorptiometry - Computed tomography (CT) scan | - Intervention determined a significant decrease in total cholesterol levels, triglyceride levels, and leptin levels compared to the placebo group - No significant changes in body weight, fat mass, and abdominal fat area in both groups | Some concern |
| [Takayuki Toshimitsu](https://pubmed.ncbi.nlm.nih.gov/?term=Toshimitsu+T&cauthor_id=33718754)  [2021] | Japan | Overweight | 92   - 46 Control - 46 Intervention   Age  20 – 64 years | - *L. plantarum* OLL2712   yogurt containing >5 × 10^9^ heat-treated OLL2712 cells   - 112 g of yogurt once daily | 12 weeks | (baseline, 4,8,12 weeks) | - Abdominal fat areas were analyzed at the level of the L4 vertebrae using CT - Body weight (to the nearest 0.1 kg) and body fat percentage (to the nearest 0.1%) were measured using a multifrequency bioelectrical impedance device - Height was measured to the nearest 0.1 cm by utilizing a compact stadiometer - Serum ketone bodies were measured using an enzyme cycling method - Serum high-sensitivity C-reactive protein (hs-CRP) was measured using the nephelometry method - Serum proinflammatory cytokines were measured using a multiplex human cytokine bead array system (Bio-Rad) - Serum adiponectin was measured using the adiponectin enzyme-linked immunosorbent assay kit (Alpco) | - Abdominal fat area increased significantly from 0-12 wk in placebo vs OLL2712 grp (dfference=8.5cm², p=0.04). - Body wt, BMI significantly increased from 0-12 wk in placebo vs OLL2712 grp - Body fat % significantly increased from 0-12 wks in both groups - Waist circumferences significantly reduced in both grps from 0-12 wk - waist to hip ratio significantly reduced from 0-12 wk in OLL2712 grp but not in placebo. - Serum HDL cholesterol significantly increased from 0-12 wk in OLL2712 grp - inflammatory markers - IL-6 significantly decreased from 0-4-12 wk in the OLL2712 grp vs placebo | Low risk |

**Table S14. Effect of *Lactiplantibacillus plantarum*in treating Oncological Disease and Surgical condition**

| Author [Year] | Country | Disease/ health condition | Participants | Intervention | Intervention Duration | Follow ups | Outcome measurement | Study Findings | Quality Assessment |
| --- | --- | --- | --- | --- | --- | --- | --- | --- | --- |
| J. Bengtsson  [2016] | Sweden | pouch function | 32   - 16 (control) - 16 (intervention) | - *L. plantarum 299 and Bifidobacterium infantis* Cure 21 - Orally/ twice a day | 21 days | Day 21 | - pouch functional score (PFS) - pouchitis disease activity index (PDAI) - levels of four fecal biomarkers of inflammation [MPO] - eosinophilic cationic protein [ECP] | - There was no difference in change in the PFS from before to after treatment between the groups (median difference: 1.00, 95% C.I. 3.00 to 0.00, p ¼ 0.119). - intervention had no effect on PDAI (median difference: 0.00, 95% C.I. 0.00–1.00, p ¼ 0.786), or on faecal biomarkers. - Significant correlations were observed between PDAI and each of the faecal biomarkers at study start. - There were no correlations between PFS or PDAI symptom subscore and the biomarkers. - PDAI endoscopic and histologic subscores correlated significantly to each of the biomarkers. | Some concern |
| CE McNaught  [2002] | United Kingdom | Surgery | 129   - 65 (control) - 64 (intervention) | - *L. plantarum 299v* - 500mL oral preparation daily from the time of study entry till a day prior to surgery | Study period2 weeks, intervention for at least 7 days | Postoperative, day 1 and 7 | - C-reactive protein (CRP) - Septic morbidity | - There was no significant difference between the two groups in terms of serum CRP measurements. - There was no significant difference in septic morbidity | High risk |
| N.P.WoodcockC.  [2004] | United Kingdom | surgery | 22   - 11 (control) - 11 (intervention) | - *L. plantarum 299v* - 500mL oral preparation daily from the time of study entry till a day prior to surgery | Study period 3 weeks, intervention for at least 5 days | 5 days from the time of operation | serum IgA, IgM, and plasma cells | - There was a significantly higher concentration of IgM at the mucosal surface in the control group (P=0.02) - No significant difference in probiotic and control groups in terms of number of plasma cell, IgA positive cellsor IgM positive cells in lamina propria of small bowel mucosa. | Some concern |
| Nada Rayes  [2007] | Germany | surgery | 80   - 40 (control) - 40 (intervention)   Age 58 ±2 years | - *Pediacoccus pentosaceus, Leuconostoc mesenteroides, L. plsntsrum, L. paracasei* + fibers - orally 20 g per day | 1 day before and 8 days after surgery | Days 1, 4 and 8 | - postoperative bacterial infection - length of hospital stays. - days in ICU   duration of antibiotic therapy | - The incidence of postoperative bacterial infections was significantly lower with probiotic (12.5%) than with control (40%) - The mean total length of hospital stays and the stay in ICU were shorter in probiotic group than in control group. - The duration of antibiotic therapy was significantly shorter in the patient’s receiving probiotic compared to control group. | Some concern |
| Peter Mangell  [2012] | Sweden | surgery | 64   - 32 (control) - 32 (intervention)   Age 46 – 80 years | - *L. plantarum*Lp299v - Orally or nasogastric tube daily | 13 days (8 days prior to surgery and 5 days after) | Day 6, week 6 and 6^th^ month | - Viable bacterial counts in rectal swab - mucosal biopsies - PCR analysis - Postoperative complication | - viable counts of lactobacilli & *Enterobacteriaceae* increased significantly at 6 weeks post-operative on rectal swabs but not mucosal swabs in probiotic group. - viable counts of all examined bacteria were significantly lower in both probiotic and placebo groups when comparing post & preoperative samples. - no significant differences b/w pts receiving probiotic and placebo in   the incidence of bacterial translocation & postoperative complications | Some concern |
| Kaźmierczak  [2020] | Poland | Cancer | 35   - 14 (control) - 21 (intervention)   Age ≥18 years old | - Sanprobi IBS® containing 1010 CFU *of L. plantarum* 299v - Oral capsule/1 at the morning and 1 at night | 4 weeks | Week 4 | - serum concentration of albumin, total protein, and total lymphocyte count (TLC). - The nutritional status evaluated by means of anthropometric and laboratory parameter Nutritional Risk Screening 2002 tool (NRS 2002 tool). The tolerance of EN was assessed with the author’s own questionnaire. - the World Health Organization Quality of Life-BREF (WHOQOL-BREF) questionnaire | - intervention group showed an increase in the serum albumin concentration compared to control (p=0.032) - changes in the frequency of vomiting and flatulence were significantly reduced at week 4 compared to baseline in intervention group (p=0.0117). - no statistical differences regarding the improvement of quality of life between the two groups were observed | Some concern |
| Chaofei Xia  [2021] | China | Cancer | 70   - 34 (control) - 36 (intervention)   Age 18 - 70 years old | - *L. plantarum, B. animalis ,L.rhamnosus and L.acidophilus* - Oral route | 7 weeks | Day 7, 12 and 21 | - acid tolerance test - anti-oxidative test - antimicrobial test - adherence assay test - DNA magnetics and extract kit - incidence of severe Oral mucositis (grade 3 or higher) | - intervention group showed significantly lower Oral mucositis. - The values of the incidence of 0, 1, 2, 3, and 4 grades of OM in the placebo group and in the intervention, group were reported to be 0, 14.7, 38.2, 32.4, and 14.7% and 13.9, 36.1, 25, 22.2, and 2.8%, respectively. - The intervention group showed a decrease in the reduction rate of CD3+ T cells (75.5% vs. 81%, p<0.01), CD4+ T cells (64.53% vs. 79.53%, p<0.01), and CD8+ T cells (75.59 vs. 62.36%, p<0.01) compared to the placebo group. | Low risk |
| Katerina Kotzampassi  [2015] | Greece | Cancer | 164   - 80 (control) - 84 (intervention) - Age 18 years and older | - *L. cidophilic LA-*5 1.75 x 10^9^ cfu, *L. plantarum* 0.5 x 10^9^ cfu, *B.  lactis BB*-12 1.75 x 10^9^ cfu and *Saccharomyces boulardii* 1.5 x 10^9^ cfu per capsule - Oral capsule/twice a day or nasogastric tube if the patient is intubated. | 14 days | Day 30 | - Post operative complication - tumour necrosis factor-alpha (TNFa) - interleukin (IL)-6 and IL-10 - enzyme immunoassay - baseline POSSUM (Physiological and Operative Severity Score for the Enumeration of Mortality   and Morbidity) | - The overall complication rate was 48.8 % in the   placebo group and 28.6 % in the probiotics group (p 0.010).   - Major benefit was found in the reduction of the rate of postoperative pneumonia (2.4 vs. 11.3 %, p 0.029), of surgical site infections (7.1 vs. 20.0 %, p 0.020) and of anastomotic leakage (1.2 vs. 8.8 %,   p 0.031).   - The time until development of first major complication was shorter in the placebo arm than in the probiotics arm - Gene expression of SOCS3 was positively related   with gene expression of TNF and of circulating IL-6 in the probiotic group but not in the placebo group. | Some concern |
| Yoon  [2021] | Korea | Cancer | 36   - 19 (control) - 17 (intervention) - Age 18 years and older | - *L. plantarum*CJLP243 - One   Sachets daily orally | 21 days | weeks 1 and 3 | - the Memorial Sloan Kettering Cancer Centre (MSKCC) - Bowel Function Index (BFI) - low anterior resection syndrome score (LARS) - European Organization for Research and Treatment of Cancer (EORTC) Quality of Life Questionnaire | - In the MSKCC BFI, the postoperative dietary scale score at week 1 was significantly higher in the probiotic group (13.1 +/- 3.8 vs. 9.0 +/- 3.0, p < 0.001). - Total scores for the MSKCC questionnaire (56.2 +/- 12.0 vs. 55.0 +/- 10.7, p=0.356) and low anterior resection syndrome scores (33.3 +/- 7.6 vs. 36.0 +/- 5.3, p=0.257) were not significantly different between the probiotic and placebo groups - No significant differences between the probiotic and placebo groups were observed postoperatively at week 1 in term of QLQ-C30 scores, including global health scale (52.0 vs. 57.5; p=0.389) and symptom scale (40.3 vs. 39.8; p=0.936) | Some concern |
| A. R Padella  [2021] | Spain | Cancer | 69   - 35 (control) - 34 (intervention) - Age 18 years and older | - *L. acidophillus DSM 24735,*   *L. plantarum DSM 24730,*  *L. paracasei DSM 24733,*  *L. delbruckeii subsp. Bulgaricus DSM24734,*  *B. breve DSM 24732,*  *B. longum DSM 24736,*  *B. infantis DSM 24737,*  *S. thermophillus* DSM24731   - Nasogastric tube | 20 days prior surgery every 2nd day | During hospitalization | - Postoperative ileus (measured using I-FEED scale) - Duration of hospital stay (measured as days of admission) | - The incidence of postoperative ileus was similar in both probiotic and control group, presence in 10/34(29.4%) in the probiotic and 11/35(31.4%) in the control group, P=0.192. - There were no significant differences regarding the need for nasogastric tube, the time required to begin tolerating a diet, restoration of bowel function, and the duration of hospital stay. | Some concern |
| G. Theodoropoulus  [2016] | Greece | Cancer | 67   - 33 (control) - 34 (intervention) - Age 18 – 80 years | - *Leuconostoc mesenteroides 77:1,*   *Pediacoccus pentosaceus 5-33,*  *L. paracasei ssp. paracasei F19,*  *L. plantarum 2362*   - orally/12g in 250ml water once daily | 15 days | Month 1, 3 & 6 | - Validated questionnaire – GIQLI. - Validated instrument - EORTC QLQ-C30 – functional. - bowel disorders (30 questions) | - patients in the probiotic group had significantly improved GIQLI global scores compared to the placebo grp (p=0.01) at 1, 3 and 6 months. - probiotic group exhibited significantly improved EORTC-QLQ -C30 diarrhea scores from baseline to 3 months (p=0.04), 6 months (p=0.003) - no significant differences were observed in EORTC-QLQ | Some concern |
| Z.H lui  [2013] | China | Cancer | 138   - 68 (control) - 70 (intervention) - Age 25 – 75 years | - *L. plantarum, B. longum, L. acidophilus* - Orally, 2g/day | 16 days (  6 days preoperative and 10 days postoperative) | Morning of the surgery, day 3, 10 and day 30 postoperative | - postoperative intestinal permeability (lactulose/mannitol test) - serum zonulin concentration (ELISA kit) - duration of postoperative infection rate - postoperative pyrexia - cumulative duration of antibiotic therapy - the incidence of postoperative infection complication | - significantly lower infection rate was seen in probiotic group compared to control p<0.05 - probiotic therapy significantly reduced serum zonulin concentrations (p<0.001), duration of postoperative pyrexia, duration of antibiotic therapy. | Some concern |

**Table S15. Effect of *Lactiplantibacillus plantarum*in treating Infectious disease**

| Author [Year] | Country | Disease/health condition | Participants | Intervention | Intervention Duration | Follow ups | Outcome measurement | Study Findings | Quality Assessment |
| --- | --- | --- | --- | --- | --- | --- | --- | --- | --- |
| Athiyyah [2019] | Indonesia | HIV | 21   - 11 [control] - 10 [intervention] - Age 2-18 years | - *L. plantarum IS*-10506 (GenBank accession number DQ860148) / Orally   + ARV Therapy   - Control: Placebo + ARV Therapy | 6 Weeks | Week 6 | - Blood lipopolysaccharide (LPS) level. - Immunological analyses: serum CD4+ T cell count, serum CD8+ T cell count, CD4+/CD8+ T cell ratio. | - The blood LPS level decreased significantly in the intervention group (p = 0.001). - No significant difference in absolute CD4+ T cell count, percent CD4+ cells, absolute CD8+ T cell count, CD4+/CD8+ T cell ratio, or fecal sIgA. - No serious adverse events were reported. | Some concern |
| M. Schunter  [2012] | U.S.A | HIV | 27   - 13 [control] - 14 [intervention] | - Intervention: *Pediococcus pentosaceus 5–33:3, Leuconostoc mesenteroides 32– 77:1, L. paracasei subsp paracasei 19, L. plantarum* 2362. - Orally | 4 weeks | Day 28 | - C-reactive protein (CRP) - Changes in T-cell count, monocyte phenotypes, CD4+ T-cells, soluble CD14 | - modest changes were seen in T-cell activation phenotype of peripheral blood lymphocytes in response to intervention therapy. - plasma levels of CRP & soluble CD-14 was unaltered with intervention | Low risk |
| Pedro Gutiérrez-Castrellón  [2022] | Mexico | Covid 19 | 293   - 146 [control] - 147 [intervention]   Age 18 - 60 years | - AB21© probiotic formula   (*L. plantarum* stains KABP022,  KABP023 and KABP033 plus *Pediococcus acidilactici*  strain KABP021)   - One Oral Capsule Daily | 30 Days | Day 15 and Day 30 | - daily report form (eDRF) - WHO Clinical Progression Scale - chest pulmonary X-ray rated according to Brixia score. - RT-qPCR | - The intervention group achieved complete symptomatic and viral clearance on day 30 by 78 (53.1%), compared to control group 41 (28.1%), significant difference at the corrected threshold of p=0.01. - Patients in the Intervention group show significantly less Covid symptoms reporting compared to the control. - The intervention was associated to lower nasopharyngeal viral load on days 15 and 30 compared to placebo. - The intervention was associated to lower radiographic scoring both on days 15 and 30. - Compared to placebo, intervention found to be associated to higher serum titers of SARS- CoV2-binding IgG and IgM on days 15 and 30. Median time to overall symptom resolution (Symptomatic clearance) was 5 days shorter in probiotic than placebo group | Low risk |
| Angurana SK [2018] | India | Sepsis | 100   - 50 [control] - 50 [intervention]   Age 3 months –12 years | - VSL#3 *L. paracasei DSM 24734, L. plantarum DSM 24730, L. acidophilus DSM 24735, L. delbrueckii subsp. bulgaricus DSM 24734, Bifidobacterium longum DSM 24736, B. infantis DSM 24737, B. breve DSM 24732, S. thermophilus* DSM 24731) - One sachet twice a day for 7 days orally or through nasogastric/orogastric tube depending on clinical status of patients. | 7 days | Day 1 and 7 | - enzyme-linked immunosorbent assay (ELISA) - Sequential Organ Failure Assessment (SOFA) - C-reactive protein (CRP) | - intervention group had lower levels of proinflammatory cytokines (“IL-6, p=0.001; IL-12p70, p=0.001; IL-17, p=0.0.01; and TNF-α, p = 0.01”) and higher levels of anti-inflammatory cytokines (“IL-10, p =0.02 and TGFβ1, p = 0.01”) as compared to placebo group. - The SOFA score on day 7 was significantly less in probiotic group than in placebo group (median [IQR] 1 [1–2] vs 3 [1–5], respectively; p=0.001). - In probiotic group, there was a statistically nonsignificant trend toward lower incidence of HCAIs (14% vs 20%; p=0.42) and duration of ICU stay (6.5 d [4–11 d] vs 9 d [6–11 d]; p=0.42) when compared with placebo group. - The probiotic group had significantly lower C-reactive protein (CRP) on day 7 than placebo group (p=0.02). - There was a nonsignificant trend toward lower incidence of healthcare-associated infections (14% vs 20%) and duration of ICU stay (6.5 vs 9 d) in probiotic group. - The probiotic group had lower incidence of feed intolerance. (14% vs 22%; p=0.21) - Mortality was similar in two groups. | Some concern |
| MariaTsilika [2022] | Greece | Sepsis | 112   - 53 [control] - 59 [intervention]   Age 18 - 80 years | - acidophilus LA-5 1.75 × 10^9^ CFU*, L. Plantarum* 0.5 × 10^9^ CFU, *Bifidobacterium lactis BB*-12 1.75 × 10^9^ CFU *Saccharomyces boulardii* 1.5 × 10^9^ CFU per capsule - two sachets, twice daily, for 15 days. - nasogastric or gastrostomy tube | 15 Days | Everyday | - chest X-ray or chest computed tomography. - blood culture - the catheter tip culture with the same antibiogram | - Administration of probiotics reduced the incidence of VAP [11.9% vs 28.3%, hazard ratio (HR) 0.34, 95% confidence interval (CI) 0.13–0.92; p=0.034] and sepsis [6.8% vs 24.5%, odds ratio 0.22, 95% CI 0.07–0.74: P=0.016]. | Some concern |
| McNaught, C. E [2005] | United Kingdom | Sepsis | 103   - 51 [control] - 52 [intervention] | - Proviva 5×10^7^CFU/mL of *L. plantarum* 299v. - Orally or via nasogastric - 500ml target volume per day - Both control and intervention received Conventional therapies included antibiotics, inotropes and adjuvant enteral or parenteral nutritional support | Administration of intervention continued until patient is discharged. | day 4, and 8 and weekly thereafter | - EndoCAb measured using (ELISA) for Endotoxin exposure. - C-reactive protein (CRP) - Serum interleukin-6 (IL-6) - Septic morbidity and mortality (APACHE II) score | - There was an increase in EndoCAb levels in intervention group between day 1 to 8 (p=0.001) and day 1 and 15 (p=0.038), suggesting a reduction in endotoxin exposure contrary to the control group which shows no rise in EndoCAb over the assessment period P > 0.05 - There were no significant differences between two groups in term of CRP - IL-6 levels were significantly lower in the intervention group compared to controls   P = 0.04   - There was no significant difference in the incidence of septic morbidity between intervention and control p > 0.05 - The mortality rate in both groups was 35%. | Some concern |
| Maria C  [2009] | Argentina | Infection/ burn wounds | 29   - 15 [control] - 14 [intervention]   Age 18 - 55 years | - *L. plantarum* - Topical (1ml/cm^2) - spread on a gauze pad and applied to the burn. | 10 days | Day 10 | - Graft evolution is assessed clinically, determination of apparition of active granulation tissue, which is recognized as bright red tissue in the bed of the wounds. | - In delayed third‐degree burns, values were 0·83 for L.  plantarum and 0·71 for SD‐Ag (control), (relative rate: +16·90%) with respect to the decrease in the bacterial load (<105 bacteria/g of tissue) and providing a granulating tissue wound bed, 0·90 in graft taking for both treatments (relative rate: 0%) and 0·75 for L.  plantarum and 0·64 for SD‐Ag (relative rate: + 17·19%) in healing. | High risk |
| Katerina Kotzampassi  [2006] | Sweden | Sepsis | 65   - 30 [control] - 35 [intervention]   Older than 18 years of age | - Intervention*: Pediococcus pentosaceus 5–33:3, Leuconostoc mesenteroides 32–77:1, L. paracasei ssp. paracasei 19; and L. plantarum* 2,362; and inulin, oat bran, pectin, and resistant starch as prebiotics - Orally, 12 g (1 sachet) per day for a 15-day | The study lasted for 12 months (intervention for 15 days) | Day 4, 7, and 15 | - C-reactive protein (CRP) - proinflammatory cytokines tumor necrosis factor-α, interleukin-6   (IL-6)   - the development of SIRS and MODS - mortality, length of stay in ICU. - Number of days under mechanical ventilation. | - The infection rate in the intervention group was 63% in comparison with 90% in the placebo group (P = 0.01). - Septic complication in the intervention group was 49% and 77% in the placebo ( P = 0.02) - Severe sepsis developed in a total of 19 patients 6 in the intervention group and 13 patients in the placebo group. - Mortality was reported to be 5 (14.3%) from the intervention group and 9 (30%) from the placebo group after MODS development. - Number of days the patients needed mechanical ventilation in Intervention group was a median of 15, and median of 26 in placebo - ICU stay was a median of 25 days for intervention and 43 days for placebo - High statistically significant differences between groups in CRP P = 0.05, with intervention having the lowest. | Some concern |

SIRS: systematic inflammatory response syndrome, MODS: multiple organ dysfunction syndrome

**Table S16. Effect of *Lactiplantibacillus plantarum* in treating hepatology disease**

| Author [Year] | Country | Disease/health condition | Participants | Intervention | Intervention Duration | Follow ups | Outcome measurement | Study Findings | Quality Assessment |
| --- | --- | --- | --- | --- | --- | --- | --- | --- | --- |
| Alisi  [2014] | Italy | Liver Disease | 40   - 20 (control) - 20 (intervention)   Mean age is 10 years old | - *S. thermophilus, bifidobacteria [B. breve, B. infantis, B. longum], L. acidophilus, L. plantarum, L. paracasei, and L. delbrueckii subsp. Bulgaricus* - 1 sachet/ day/ orally in children younger than 10 years, and 2 sachets in children older than 10 years | 4 months | Month 4 | - homoeostatic model assessment (HOMA), (ALT) - (ELISA) - Liver ultrasound - Liver histology | - The probability that children supplemented with probiotic had none, light, moderate or severe FL at the end of the study was 21%, 70%, 9% and 0% respectively with corresponding values of 0%, 7%, 76% and 17% for the placebo group (p<0.001). - No between-group differences were detected in triglycerides, ALT and  HOMA. | Low risk |
| v. w. s. wong  [2013] | Hong kong | Liver Disease | 20   - 10 (control) - 10 (intervention)   Age 18-70 years | - *L. plantarum, L. delbruekii ssp bulgaricus, L. acidophilus, L. rhmnosus, B. bifidum* - Orally formula twice a day | 6 months | Month 6 | - IHTG- proton magnetic resonance spectroscopy - liver stiffness by transient elastogrophy Fibroscan machine - changes in ALT and AST | - IHTG decreased in the probiotic group (p = 0.034). in contrast, IHTG remained static in the control group (p=0.55) - Probiotic group had greater reduction in AST level compared to the control group. - There was no significant effect of probiotic treatment on the liver stiffness and ALT | Some concern |

Alanine transaminase (ALT), aspartate aminotransferase (AST)
